# New aspects in deriving health-based guidance values for bromate in swimming pool water

**DOI:** 10.1007/s00204-022-03255-9

**Published:** 2022-04-06

**Authors:** C. Röhl, M. Batke, G. Damm, A. Freyberger, T. Gebel, U. Gundert-Remy, J. G. Hengstler, A. Mangerich, A. Matthiessen, F. Partosch, T. Schupp, K. M. Wollin, H. Foth

**Affiliations:** 1 Institute of Toxicology and Pharmacology for Natural Scientists, Christiana Albertina University Kiel, Kiel, Germany; 2 Department of Environmental Health Protection, State Agency for social Services (LAsD) Schleswig-Holstein, Neumünster, Germany; 3 University Emden/Leer, Emden, Germany; 4 Department of Hepatobiliary Surgery and Visceral Transplantation, University Hospital, Leipzig University, Leipzig, Germany; 5 Research and Development, Pharmaceuticals, RED-PCD-TOX-P&PC Clinical Pathology, Bayer AG, Wuppertal, Germany; 6 Federal Institute for Occupational Safety and Health (BAuA), Dortmund, Germany; 7 Institute for Clinical Pharmacology and Toxicology, Universitätsmedizin Berlin, Charité Berlin, Germany; 8 Leibniz Research Centre for Working Environment and Human Factors (IfADo), TU Dortmund University, Dortmund, Germany; 9 Molecular Toxicology, Department of Biology, University of Konstanz, Konstanz, Germany; 10 Central Unit for Environmental Hygiene, University Hospital Schleswig-Holstein (UKSH), Kiel, Germany; 11 Department of Toxicology, Fraunhofer-Institute for Toxicology and Experimental Medicine (ITEM), Hannover, Germany; 12 Department of Chemical Engineering, University of Applied Science Muenster, Steinfurt, Germany; 13 Formerly Public Health Agency of Lower Saxony, Hannover, Germany; 14 Institute of Environmental Toxicology, University of Halle, Halle/Saale, Germany

**Keywords:** Bromate, Swimming pool water, Mode of action, Exposure, Disinfection, Risk assessment

## Abstract

Bromate, classified as a EU CLP 1B carcinogen, is a typical by-product of the disinfection of drinking and swimming pool water. The aim of this study was (a) to provide data on the occurrence of bromate in pool water, (b) to re-evaluate the carcinogenic MOA of bromate in the light of existing data, (c) to assess the possible exposure to bromate via swimming pool water and (d) to inform the derivation of cancer risk-related bromate concentrations in swimming pool water. Measurements from monitoring analysis of 229 samples showed bromate concentrations in seawater pools up to 34 mg/L. A comprehensive non-systematic literature search was done and the quality of the studies on genotoxicity and carcinogenicity was assessed by Klimisch criteria (Klimisch et al., Regul Toxicol Pharmacol 25:1–5, 1997) and SciRAP tool (Beronius et al., J Appl Toxicol, 38:1460–1470, 2018) respectively. Benchmark dose (BMD) modeling was performed using the modeling average mode in BMDS 3.1 and PROAST 66.40, 67 and 69 (human cancer BMDL_10_; EFSA 2017). For exposure assessment, data from a wide range of sources were evaluated for their reliability. Different target groups (infants/toddlers, children and adults) and exposure scenarios (recreational, sport-active swimmers, top athletes) were considered for oral, inhalation and dermal exposure. Exposure was calculated according to the frequency of swimming events and duration in water. For illustration, cancer risk-related bromate concentrations in pool water were calculated for different target groups, taking into account their exposure using the hBMDL_10_ and a cancer risk of 1 in 100,000. Convincing evidence was obtained from a multitude of studies that bromate induces oxidative DNA damage and acts as a clastogen in vitro and in vivo. Since statistical modeling of the available genotoxicity data is compatible with both linear as well as non-linear dose–response relationships, bromate should be conservatively considered to be a non-threshold carcinogen. BMD modeling with model averaging for renal cancer studies (Kurokawa et al., J Natl. Cancer Inst, 1983 and 1986a; DeAngelo et al., Toxicol Pathol 26:587–594, 1998) resulted in a median hBMDL_10_ of 0.65 mg bromate/kg body weight (bw) per day. Evaluation of different age and activity groups revealed that top athletes had the highest exposure, followed by sport-active children, sport-active adults, infants and toddlers, children and adults. The predominant route of exposure was oral (73–98%) by swallowing water, followed by the dermal route (2–27%), while the inhalation route was insignificant (< 0.5%). Accepting the same risk level for all population groups resulted in different guidance values due to the large variation in exposure. For example, for an additional risk of 1 in 100,000, the bromate concentrations would range between 0.011 for top athletes, 0.015 for sport-active children and 2.1 mg/L for adults. In conclusion, the present study shows that health risks due to bromate exposure by swimming pool water cannot be excluded and that large differences in risk exist depending on the individual swimming habits and water concentrations.

**Supplementary Information:**

The online version contains supplementary material available at 10.1007/s00204-022-03255-9.

## Introduction

Bromate anion is a disinfection by-product (DBP) in drinking and swimming pool water that occurs as a result of ozonation or chlorination of bromide-containing water or using ozone-bromide treatment for disinfection. Since the (European) Directive 2006/7/EC on the management of bathing water quality does not apply to swimming pools, requirements for the quality of swimming pool water are defined within the framework of the legislation of EU member states. In Germany, the legal basis for monitoring the quality of swimming and bathing pool water is the Infection Protection Act (IfSG 2000, para. 37). Swimming pool water disinfectants are product-type 2 usages as laid down in ‘Regulation (EU) No 528/2012 concerning the making available on the market and use of biocidal products’. Insofar, the DBP bromate is also covered by Regulation (EU) No 528/2012 (ECHA 2017a). Biocidal products require an authorization before they can be placed on the market, and the active substances contained in that biocidal product must be previously approved. The comprehensive requirements for human health risk assessment in the context of the approval and authorization of biocides are described in ECHA (2017b) and ECHA (2018). ECHA’s first tier approach for the human toxicological risk assessment for DBPs from oxidative acting biocidal products in PT2 consists of simply comparing measured DBP concentration of selected DBPs to existing limits for swimming- and/or drinking-water for these DBPs. In principle, the use of drinking-water limits should be viewed as first tier approach which can be refined if needed with a more specific swimming-water limit (ECHA 2017a).

Various thresholds for bromate in pool water (Anses 2012; DIN 2012; RIVM 2014; UBA 2014; EDI 2016; ECHA 2017a) have been proposed, ranging from 8.7 to 2000 µg/L. Often, drinking water is used as fill-up water for swimming pools. For risk evaluation, it is important to know the maximum background concentration of bromate in the fill-up water. Drinking water guidance values for bromate were set at 10 µg/L (World Health Organization 2017; Health Canada 2018; US Environmental Protection Agency, Office of Water 2018; EU 2020). The U.S. EPA’s MCLG (Maximum Contaminant Level Goal, a non-enforceable health benchmark goal) has set the level goal of bromate at zero. The Directive (EU) 2020/2184 indicates that, where possible without compromising disinfection, EU member states should strive for lower bromate values in the future. The drinking water values are of particular interest when cancer estimates have been taken into account. If sea water is utilized as fill-up water, its natural bromide content determines the background concentration before disinfection. In natural sea water, bromate may be present below 1 µg/L (Lim and Shin 2012). The current bathing water guidance values differ by several orders of magnitude, depending partly on the evaluation of the carcinogenic mode of action (MOA), as well as on the exposure assessment. According to CLP regulation EU/1272/2008, potassium bromate is classified as carcinogen in category 1B (presumed to have carcinogenic potential for humans, classification is largely based on animal evidence). Bromate’s mutagenicity has not been classified in the CLP regulation.

Current discussions on the approval of the ozone-bromine method for the disinfection of bathing water, the in comparison to other countries relatively high bromate limit value of 2 mg/L bathing water in the recently implemented baths hygiene regulation of Schleswig–Holstein, Germany, and recurrent findings of even higher bromate concentrations in German seawater pools were the starting-point for the present study.

The aim of this study was (a) to provide data on the occurrence of bromate in pool water, (b) to re-evaluate the carcinogenic MOA of bromate in the light of existing data, (c) to assess the possible exposure to bromate via swimming pool water and (d) to inform the derivation of cancer risk-related bromate concentrations in swimming pool water. For the development of these risk-based guidance values, all relevant exposure routes, different target groups and exposure scenarios were considered.

## Physical–chemical properties, formation, occurrence of bromate and guidance values in swimming pool water

As the dissolved bromate anion is the chemical entity that leads to the relevant health effects, different salts, e.g. sodium vs. potassium bromate, are not evaluated separately.

### Physical–chemical data

Bromate is an anion that is associated with a cation, forming salts with characteristic physico-chemical properties (molecular weight bromate = 127.9 g/mol, e.g. Potassium Bromate = 167.01 g/mol). As strong electrolytes with a water solubility of more than 10 g/L at 20 °C, bromate salts dissociate in aqueous media. Therefore, the mode of toxic action of the bromate ion is expected to be independent of the counter-ion (ECHA 2010, 2019). The Henry coefficient has not been determined for potassium bromate or sodium bromate; however, an estimate (based on data for sodium oxide and an assumption of 99% dissociation) indicates a Henry-coefficient of 2.53 × 10^–13^ Pa × m^3^/mol (**S1.1**). This low vapor pressure indicates that exposure to bromate by inhalation may occur only in the presence of bromate-loaded aerosols. Further physical data (**S1.2)** and analytical methods (**S1.3**) are summarized in the supplemental materials.

### Formation of bromate by water disinfection in the presence of bromide ions

Water may contain a natural background of bromide ions in the range of 10 – 40,000 µg/L (Table 1). In pool water, bromate may be formed during disinfection if bromide ions are present.

**Table 1:** Bromate concentrations in relation to bromide and the disinfection method (ranges or means)

Water quality	Disinfection method	Bromide [µg/L]	Bromate [µg/L]	References
Drinking water	O_3_, H_2_O_2_	10 – 40	5 – 25	Arvai et al. (2012)
Drinking water	O_3_	45 – 50	< 2 – 40	Gunten (2003)
Drinking water	O_3_	220 – 3,330	5 – 141	Gunten and Hoigne (1994)
Drinking water	NaOCl	200	2 – 4	Fang et al. (2014)
Drinking water	Cl_2_	56	10 – 50	Huang et al. (2008)
Drinking water	Cl_2_	160 – 800	~ 128 – 770	Liu et al. (2012)
Seawater pool^a^	O_3_	26,600	750	Shi et al. (2013)
Seawater pool^a^	Cl_2_	18,500	810	Shi et al. (2013)
Freshwater pool	O_3_	20,000 – 40,000	< 100 – 100	Hoffmann (2015)
Pool water, n.o.s.^c^	O_3_	400 – 1600	80 and 511	Michalski and Mathews (2007)
Pool water, n.o.s.^c^	O_3_ + Bromide	No data	990 (median), 2000 (peak)	Donzé et al. (2012)
Pool water, n.o.s.^c^	O_3_	No data	< 5	Donzé et al. (2012)
Pool water, n.o.s.^c^	Chlorine	No data	< 5	Donzé et al. (2012)
Pool water, n.o.s.^c^	Hypochlorites^b^	400 – 1600	< 6	Michalski and Mathews (2007)
Pool water, n.o.s.^c^	NaOCl	No data	1400 (peak)	Donzé et al. (2012)
Pool water, n.o.s.^c^	NaOCl	No data	1400 (peak)	Donzé et al. (2012)
Pool water, n.o.s.^c^	Hypochlorites^b^	No data	< 2 – 48	Righi et al. (2014)

^a^Lab tests, simulating a seawater pool

^b^Sodium hypochlorite, calcium hypochlorite or trichloro isocyanuric acid

^c^Not otherwise specified

During disinfection processes chlorine, hypochlorites and ozone may oxidize bromide (Br–) to bromine (Br_2_) and further to bromate (Huang et al. 2008; Liu et al. 2012; Shi et al. 2013; Fang et al. 2014). Bromide ions (Br–) react rapidly to hypobromite (OBr–) in the presence of ozone (1) and may subsequently and unintentionally disproportionate to bromate (BrO_3_^−^) at elevated pH-values, or may also be oxidized to bromate by ozone (2).^1^

1 $${\mathrm{O}}_{3}+ {\mathrm{Br}}^{-}\to {\mathrm{O}}_{2}+{\mathrm{OBr}}^{-},$$

2 $$3 {\mathrm{OBr}}^{-}\to {\mathrm{BrO}}_{3}^{-}+2 {\mathrm{Br}}^{-}; 2 {\mathrm{O}}_{3}+{\mathrm{OBr}}^{-}\to 2 {\mathrm{O}}_{2}+{\mathrm{BrO}}_{3}^{-}$$

The yield of bromate generated during ozonation of water is dependent on several factors such as pH, total dissolved organic carbon (TOC), ammonia, bromide and temperature. An empirical relation between the yield of bromate and the aforementioned parameters has been published (Haag and Hoigne 1983; Siddiqui and Amy 1993; Song et al. 1996). A detailed description of the reactions is available in Supplemental Information S2 and S3. The influence of several parameters on bromate formation may explain why bromate/bromide ratios differ widely between samples taken from waters treated with the same disinfection method (Table 1). For the ozone-bromide treatment of pool waters, 20–40 mg/L bromide is added (Brugger 2014; Hansen et al. 2016) to achieve an ozone-bromide system with acceptable biocidal activity.

Increased bromate concentrations in pool water also can result from an impurity of the disinfection solution. For example, sodium bromate can increase over time in sodium hypochlorite solution (Javel water) used for disinfection, if the chloride solutions for the synthesis of hypochlorites contain bromide ions, these hypochlorite disinfectants may reach bromate concentrations of 2.5–38 mg/kg, and peak concentrations of 77 mg/kg (Binetti and Attias 2007). A further source of bromate is the electrolytic generation of chlorine in pool water, if bromide containing sea water is used instead of pure sodium chloride brine (World Health Organization 2017).

### Occurrence of bromate in marine and fresh pool water

Surveillance data for bromate in pool water (not published) reveal the impact of different water sources on the bromate concentrations (Table 2).

**Table 2:** Bromate concentrations measured in fresh water and seawater pools tested between 2017 and 2020 in northern Germany^1^

Concentration [mg/L]	Number of analyzed samples and			
	Fresh water		Seawater	
	Samples	Pools	Samples	Pools
< 0.02	156	23		
0.02–0.05	32	14		
0.05–0.1	15	7		
0.1–0.2 (seawater < 0.2)	15	8	55	9
0.2–0.5	7	3	6	3
0.5–1.0	1	1	15	6
1.0–2.0	2	1	21	7
2.0–5.0	1	1	40	5
5.0–10			39	4
10–20			14	3
20–30			5	2
> 30			2	2
Sum	229	31 (1)	197	11 (1)

Routine analyses of samples from different swimming pools were performed according to DIN EN ISO 15061 with ion-chromatography and suppressed conductivity detection between January 2017 and May 2020. To avoid analytical problems from the high content of sodium chloride, seawater samples were diluted prior to analysis with distilled water by 1:7 to 1:10 depending on the electrical conductivity of the sample. With regard to the higher matrix effects of swimming pool water compared to drinking water, the lower limit of quantitation (LLOQ) was set to 20 µg/L for fresh water pools and 200 µg/L for seawater pools. Standards have been issued for the analysis of bromate in water samples, including pre-analysis treatment of samples to avoid false-positive and false-negative results. Detection limits are 0.5 µg/L for standard methods. The number of samples per pool range between 1 and 19 within the examined period

A total of 229 samples taken between 2017 and May 2020 from 31 fresh water facilities and 197 samples from 11 seawater facilities in northern Germany, partially including several pools, were analyzed. Fresh water pools had low concentrations of bromate, with 89% of pools containing less than 100 µg/L and a maximum of 1200 µg/L. Higher concentrations were detected in seawater pools; with 51% of the analyzed samples containing higher than 2000 µg/L. It should be considered that these concentrations were obtained from pools with disinfected seawater and that concentrations in native seawater are much lower. Bromate was not detected in untreated seawater using detection limits of 60 µg/L and 2 µg/L (Chen et al. 2006; Zakaria et al. 2011). Background concentrations of 0.1–0.6 µg/L bromate in five sea water samples from the south sea at Korea were reported (Lim and Shin 2012). Although high concentrations of bromate were detected in some seawater pools, there are also other seawater pools with bromate concentrations below the lower limit of quantitation (LLOQ) of 200 µg/Lover the entire period examined. Moreover, individual seawater pools with disinfection may exhibit a large variation over time (Table 3); rapid increases in bromate concentration within two months have been found (Table 4).

**Table 3:** Seawater pool with varying concentrations of bromate over time

Seawater pool no. 74	
Sampling date	Bromate [mg/L]
01.02.2017	11
17.08.2017	17
12.02.2018	11
13.08.2018	34
11.02.2019	7.1
19.08.2019	22
11.02.2020	5.8

**Table 4:** Seawater pool with steeply increasing concentrations of bromate after a complete water exchange

Seawater pool no. 20945	
Sampling date	Bromate [mg/L]
16.01.2018	2.5
19.02.2018	6.8
20.03.2018	14.6

An overview of bromate concentrations in pool and drinking water reported in the literature is given in Table 1. Bromate concentrations in drinking water ranged from < 2 to 770 µg/L, whereas levels reported for pool water ranged from < 2 to 2000 µg/L. In Germany, drinking water analysis does not include bromate on a regular basis. When bromate was analyzed, the drinking water limit for bromate was exceeded in less than 1% of the samples between 2014 and 2016 (Bundesministerium für Gesundheit and Umweltbundesamt 2018).

### Pool water standards and guidance values for bromate

Pool water standards are set to ensure a certain level of antimicrobial activity for the protection of swimmers against infections. If ozonation is used for pool water disinfection, bromide ions may have been added to achieve the legally required disinfection capacity defined by sanitation standards (ozone-bromide treatment). Existing sanitation standards for pool water are given in Table 5.

**Table 5:** Pool water specification according to sanitation guidelines/standards

Parameter	WHO^a^	USA^b^	UK^c^	Germany^d^	Switzerland^e^	The Netherlands^f^	Europe^g^	France^h^
pH	7.2–7.8	7.2 – 7.8	7.2 – 7.4	6.5 – 7.8	6.8 – 7.8			6.9 – 8.2
pH (bromine ozone methods)^2^				6.8 – 7.2	6.8 – 7.2			
Chlorine (if used as active disinfectant), min – max [mg/L]	0.5–3.0	1–3	0.5 – 3.0	0.3 – 1.0	0.2	0.5 – 1.5 (indoor) or 3.0 (outdoor)		0.4 – 1.4
Active (bound) bromine (if used as precursor for active disinfectant) [mg/L]	4–6	3–4	–		0.5			1.0–2.0
Bromide (if used as precursor of active disinfectant) [mg/L]		–	–		50			
Bromate [µg/L]		–	–	**2000**	**200**	**100**	**100**	**2480** (infants, 6 month–2 years), **372** (children, 2–15 years), **17** (sport-active children), **372** (occasional adult swimmer), **9.9** (sport-active adult); **8.7** (competitive athlete), **212** (lifeguard)

^a^World Health Organisation (2006)

^b^U.S Department of Health and Human Services, Centers for Disease Control and Prevention (2016)

^c^PWTAG (2019)

^d^DIN 19,643–1:2012–11;Schleswig–Holstein (2018)

^e^Schweizerische Eidgenossenschaft (2016)

^f^RIVM (2014)

^g^ECHA (2017)

^h^Anses (2012), separate maximum bromate concentrations were calculated for different user groups due to different average daily uptakes of bathing water

An overview of guideline values for bromate in pool water and drinking water, focusing the toxicological data on which their derivation is based, is given in Table 6. Carcinogenicity has been assessed as the critical effect and key endpoint of concern, whereby linear extrapolation using a non-threshold approach has been used in most cases.

**Table 6:** Basic information used in the derivation of bromate pool water and drinking water guideline values

	Bromate value [µg/L]	Pivotal study	Species	Critical effect, target organ	Dose descriptor (oral uptake)	Extra carcinogenic risk	Reference
*Pool water*							
Anses	8.7–2480	DeAngelo et al. (1998)	Rat	Renal tumors	0.0143 µg/kg bw/d (body dose), 0.7 [mg/kg bw/d]^−1^ (Slope factor)	1: 100,000	Anses (2012)
Germany	2000^a^	n.a	n.a	n.a	n.a	n.a	DIN 19,643–1:2012–11 2012; UBA (2014)
The Netherlands	100	n.a	Rat	Renal tumors	0.05 µg/kg bw/d	1: 100,000	RIVM (2014)
Switzerland	200	n.a	n.a	n.a	n.a	n.a	EDI (2016)
ECHA	100^b^	n.a	Rat	Renal tumors	0.05 µg/kg bw/d	1: 100,000	
*Drinking water*							
U.S. EPA, IRIS	0.5	DeAngelo et al. (1998)	Rat	Kidney renal tubule tumors, mesotheliomas, and thyroid follicular cell tumors	0.7 [mg/kg bw/d]^−1^ (Oral Slope Factor), 2 × 10^–5^ [µg/L]^−1^ (Drinking Water Unit Risk)	1: 100,000	US EPA (2001, 2021)^c^
OEHHA	0.1 (Public Health Goal) 10 (MCL) 50	DeAngelo et al. (1998)	Rat	Mesothelioma, kidney tumors, and thyroid tumors Renal pelvis urothelial hyperplasia	0.21 mg/kg bw/d 0.011 mg/kg bw/d (ADD, acceptable daily dose)	1:1,000,000 1:10,000 (Cancer Risk at MCL) –	OEHHA (2009)
WHO	10 (Provisional Guideline Value) 2	DeAngelo et al. (1998)	Rat		0.19 mg/kg bw/d	1:100,000	World Health Organization 2017^f^
U.S. EPA, Office of Water	zero (MCLG^d^) 10 (MCL^d^) 140 (DWEL^d^) 5	DeAngelo et al. (1998)	Rat		4 µg/kg bw/d (RfD)	– 1:10,000	US Environmental Protection Agency, Office of Water (2018)^e^
Health Canada	10 (MAC) ^g^ 4 (carcinogenic effects) 40 (non-cancer effects), (HBV, health-based value)	DeAngelo et al. (1998)		Tumors of the testicular mesothelium Urothelial hyperplasia	2.6 × 10^−3^ mg/L^−1^ (Cancer Slope Factor) 49.7 mg/L (BMDL_10_), TDI = 0.001 mg/kg bw/d	1:100,000	Health Canada (2019)^h^
European Union	10						EU (2020)^i^

^a^The detailed rationale can be seen in UBA (2009) (unpublished report)

^b^ECHA (2017) has adopted RIVM (2014) guideline value

^c^The current IRIS data is based on EPA´s report first published in 2001

^d^MCLG (Maximum Contaminant Level Goal); MCL (Maximum Contaminant Level); DWEL (Drinking Water Equivalent Level) (DWEL obtained from the RfD of 0.004 mg/kg bw/d)

^e^The toxicological data used is based on US EPA (2001)

^f^The detailed rationale can be seen in WHO (2005)

^g^Maximum Acceptable Concentration. The MAC takes into consideration limitations in analytical methodology and treatment technologies

^h^The corresponding Guideline Technical Document is Health Canada (2018)

^i^The European Union has adopted WHO´s (2017) guideline value

## Toxicokinetics

The kinetics of bromate has been studied in rats following oral administration. The only data on dermal absorption are from unpublished studies in guinea pigs, summarized by the Cosmetic Ingredient Expert Review Panel (Anderson 1994).

### Absorption

#### Oral uptake

Following administration of bromate (0.625–100 mg/kg bw) by gavage to rats, the maximum plasma concentration was reached within 15 min (Fujii et al. 1984), indicating fast absorption. Comparison of AUCs following intravenous and oral administration, the latter by gavage, enabled a calculation of the bioavailability. When doses between 0.077 and 15.3 mg/kg bw (only oral administration) were investigated, a linear relationship between AUC and dose was observed between 0.077 and 1.9 mg/kg bw for the intravenous and oral routes; however, at higher doses, the AUC increases exceeded dose proportionality, suggesting saturation of clearance processes (Bull et al. 2012). The percentage of oral absorption was calculated to vary between 19.5 and 24.6%.

Investigations using real and synthetic gastric juice indicate some pre-absorptive breakdown in the stomach (Keith et al. 2006; Cotruvo et al. 2010) which may reduce the amount of absorbable bromate. However, as the rate of breakdown was very slow, it was assumed to be irrelevant under the typical physiological stomach retention time.

Human data on absorption are scarce. In a case report, acute bromate poisoning of a 2-year-old male (13 kg) was described. He ingested 1 to 2 oz (29.5–59 mL) of a permanent wave neutralizer containing 10–12 g/100 mL bromate (dose: between 3 and 7 g; 230 and 540 mg/kg bw). The peak concentration (approximately 160 µg/mL serum) was reached 12 h after ingestion. The total amount of bromide recovered from dialysate and the urine over 6–48 h after ingestion was 1,850 mg (140 mg/kg bw). The boy was admitted to the pediatric intensive care unit, where a urinary catheter was inserted after a decrease of urinary output. He was released from hospital a few days later. Follow-up examinations revealed normal hearing, renal function and urinalysis findings (Lichtenberg et al. 1989).

#### Inhalation uptake

Data on the uptake of bromate by the inhalation route are not available.

#### Dermal uptake

Primary data for the dermal absorption of bromate are not available. An unpublished ex vivo dermal absorption study with guinea pig skin is summarized by the Cosmetic Ingredient Expert Review Panel (Anderson 1994). A Kp value (Kp = permeability coefficient) of 4.29 × 10^–6^ cm/min can be calculated from the reported data to approximate the dermal uptake of bromate for swimmers (S4). This value is in a typical range for anions; Kp values were 5.5 × 10^–5^ cm/min for bromide, 1.1 × 10^–4^ cm/min for phosphate in rabbits in vivo, 7.3 × 10^–7^ cm/min for bromide in male volunteers (Tregear 1966), and 3.8 × 10^–5^ cm/min for bromide on porcine skin in vitro (Paweloszek et al. 2016).

No studies could be identified for long-term exposure of the whole body in water. However, Gattu and Maibach (2010, 2011) reviewed studies in which the influence of physical or chemical stress and skin disease on dermal absorption of bromate was investigated. They concluded that even in the worst case, the increase in dermal absorption was modest and did not exceed a factor of ten. Likewise, Felter et al. (2017) investigated the influence of diaper rash on skin absorption and reported a 1.45-fold increase compared to healthy skin for substances with low absorption of less than 10% of the applied dose. Considering that human skin is generally less permeable to chemicals than guinea pig skin due to fewer hair follicles, it was assumed that the higher permeability of macerated or injured skin is covered by the use of a permeability coefficient derived from a guinea pig skin absorption model. Therefore, no further uncertainty factor was used for the assessment of dermal bromate exposure.

### Distribution

Fujii et al. (1984) reported that in rats after oral dosing with potassium bromate, no bromate was found in any organ or blood 24 h after dosing, although it was found in large amounts in urine. However, bromide concentrations were increased in all organs, particularly in kidney, and in urine. This is consistent with reduction of bromate to bromide in body tissues by glutathione and other thiols (Tanaka et al. 1984). When the bromate dose was increased above 5 mg/kg bw by gavage, the concentration of ^18^O-labeled bromate in the tissue of the kidneys increased strongly. This may be explained by saturation of the renal elimination of bromate and metabolites at such high concentrations (Delker et al. 2006).

Fisher and Bull (2006) hypothesized that the organ-specificity of bromate-induced adverse effects is the consequence of effective transport of bromate by the sodium iodide symporter (NIS) into the cells of the respective organs. NIS is an energy-dependent transporter which shuttles iodide from the blood stream into cells. In addition, bromate and its stable metabolite bromide are substrates of NIS (Eskandari et al. 1997). However, except for the thyroid, there is only limited evidence for the presence of an active NIS in target tissues.

### Metabolism

Bromate is a strong oxidizer, which is effectively reduced to bromide by thiol compounds such as GSH and cysteine (Cys). Rat liver and kidney homogenate, as well as red blood cells, showed the highest activity for degradation of bromate under physiological conditions (Tanaka et al. 1984). Furthermore, bromide is yielded stoichiometrically by GSH-mediated degradation of bromate, which corresponds well with the fact that in vivo the bromide concentration increases in the kidney, pancreas, stomach, red blood cells and plasma and urine of rats 24 h after oral administration of bromate (Fujii et al. 1984). Further information on metabolism was derived from a newer in vitro study in which bromate was added to fresh rat blood. Initially, bromate at concentrations of less than 320 µM was rapidly reduced to bromide. Thereafter, a lower rate of transformation was observed. This slower rate was also measured in samples with higher initial bromate concentrations (Bull et al. 2012). It was proposed that degradation of bromate in vivo occurs to a high degree in plasma. In addition, gastric transformation may take place when bromate is administered orally. In gastric juice, bromate has a half-life of about 20–30 min (Keith et al. 2006).

### Elimination and volume of distribution

The average terminal half-life of orally administered bromate was 37 min, according to Bull et al. (2012). Using AUC data and the data from Bull et al. (2012) after intravenous administration, the clearance of bromate value was calculated to be 0.76 ± 0.34 L/kg/h (mean ± SD) and the resulting volume of distribution (according to volume of distribution = clearance/(0.693/half-life)) was calculated to be 47.3 L for a 70 kg person, indicating that bromate is distributed into the body water. From a study with continuous oral administration via drinking water, the daily renal excretion of bromate was 9.1 ± 1.6% (mean ± SD) of the dose and the sum of bromate and bromide excretion was 75.2 ± 8.7% (mean ± SD) of the dose (Bull et al. 2012).

### PBPK models

Health Canada employed the results of a PBPK model in its risk assessment (Health Canada 2018). However, besides a preliminary structure of the model for bromate and its metabolite bromide (Fisher and Bull 2006), no further publication was identified in the literature. In addition, the risk assessment document did not contain further details of the model. As explained in a second document (Environment Canada and Health Canada 2010), the model results included some uncertainties, as concentrations in human biological fluids were unavailable for model validation, and differences between rats and humans in bromate kinetics may exist.

In an attempt to estimate human equivalent concentrations in drinking water, Campbell et al. (2019) developed a PBPK model using published experimental results from rat studies (Bull et al. 2012) to calibrate the model. The authors constructed a rat model that was extrapolated to human using species-specific physiological parameters and interspecies scaling factors (flows: body weight^3/4^, first order rate constants: body weight^−1/4^). The human model was used to simulate the concentrations of potassium bromate in drinking water in humans, assuming a water consumption of 2 L/d, which would lead to an equivalent internal dose, defined as average steady state plasma concentration, compared to the internal dose in three different carcinogenicity studies in rats (Kurokawa et al. 1986a, b; DeAngelo et al. 1998). Using this procedure, human equivalent drinking water concentrations (HEC) could be established. The HEC factors ranged from 1.92 to 3.47. As the upper limit of this range is close to the standard human adjustment factor of 4, we used this standard value when converting the rat BMDL to a human BMDL.

## Health effects

### Effects in humans

Systematic studies of the toxicity of bromate in humans are lacking and effects of long-term exposure remain unknown. In the literature, cases of accidental and suicidal intoxications have been published. The target organs of acute toxicity are the kidney and the inner ear. Acute doses which are reported to result in kidney toxicity and ototoxicity are above 100 mg/kg bw, according to a case series in Kurokawa et al. (1990) and Mack (1988). Ototoxicity has been reviewed by Campbell (2006). Further case reports and information are given by Dunsky (1947), Gradus et al. (1984), Kuwahara et al. (1984), and Quick et al. (1975).

### Animal studies

The quality of the main animal key studies was evaluated using SciRAP (Science in Risk Assessment and Policy, http://www.scirap.org/), which is a web-based reporting and evaluation tool (Table 9). Several criteria for the evaluation of reliability and relevance of the studies were used, whereby the more precisely the studies were described the higher the scores (given percentage) for reporting and methodological quality (see supplementary SciRAP files) and the lower the total tier (given in Tables 7, 8, 9). The evaluation comprises criteria on the reporting quality, methodological quality und relevance of each study. Each criterion can be marked as fulfilled, partially fulfilled, not fulfilled or not applicable, corresponding to the different color codes in the table. Accordingly, the study by Dodd et al. (2013) is described in the highest detail, whereas Guo et al. (2001) lacked some information.

**Table 7:** Subacute, subchronic and chronic animal studies (for carcinogenicity see Table 8). For detailed information on the study quality (= tier) see Table 9

Species	Administration	Duration	Dosages of potassium bromate	Observations	NOAEL [mg/kg bw/d]	LOAEL [mg/kg bw/d]	Tier [SciRAP]	References
*Subacute and subchronic studies*								
F344 rats, m, f, (10/sex/dose)	Drinking water	13 weeks	13.35/13.95, 26.7/27.9, 53.4/55.8, 111.25/116.25, 222.5/232.5, 445/465, 890/930 mg/kg bw/d for males and females, respectively	All animals died within 7 days ≥ 222.5/232.5 mg/kg bw/d. Sign. decreased bw gain in males at 111.25 and 222.5 mg/kg bw/d and regenerative changes in male kidneys at 111.25 mg/kg bw/d. changes in clinical chemistry in m and f at 111.25/116.25 mg/kg bw/d Supplemental study for 12 weeks (only males) Transient droplets in renal tubules being of eosinophilic bodies at 53.4 mg/kg bw/d normalized after treatment	53.4/55.8	111.25/116.25		Unpublished data, cited by Kurokawa et al. (1990)
F344 rats, (10 m/dose)	Drinking water	2 or 13 weeks	0, 0.4, 1.6, 8.1, 16.5, 34.9 mg/kg bw/d	Increased kidney weight at 34.9 mg/kg after 2 and 13 weeks, hyaline droplets in renal tubules ≥ 16.5 mg/kg after 2 weeks and 34.9 mg/kg after 4 weeks	8.1	16.5	1	Dodd et al. (2013)
F344 rats, (5/sex/dose)	Drinking water	4 weeks	0, 1.88/1.82, 3.75/3.63, 7.5/7.26, 15.63/15.13, 31.25/30.25, 62.5/60.5 mg/kg bw/d for males and females, respectively	8-oxodG formation significantly increased in both sexes ≥ 31.25/30.25 mg/kg, α2u-Globulin accumulation in kidneys in males ≥ 15.63 mg/kg	7.5/7.26	15.63	2	Umemura et al. (2004)
Swiss Mice, (12 m/dose)	Drinking water	2 weeks	348 mg/kg bw/d	Oxidative stress and protein oxidative damages in the liver, degenerative changes like hepatic steatosis, leucocyte infiltration, hepatocyte vacuolization and necrosis	n/a	348	n/a	Ben Saad et al. (2016)
Swiss Webster mice, sex not stated (45 in total)	Gavage	6 weeks	100, 200 mg/kg bw/d	Hematological changes, impaired renal and hepatic histology ≥ 100 mg/kg	n/a	100	n/a	Altoom et al. (2018)
Albino mice, (10 m/dose)	Gavage	6 weeks	100, 200 mg/kg bw/d	Neurological symptoms, decreased neurotransmitter and glutathione levels, extensive damage in the histological sections of the medulla and cerebral cortex of brains ≥ 100 mg/kg	n/a	100	n/a	Ajarem et al. (2016)
B6C3F1 mice, (8 f/dose)	Drinking water	4 weeks	15.28, 38.2, 76.4, 114,6, 152.8 mg NaBrO_3_/kg bw/d	Increased spleen weight ≥ 15.28 mg/kg, dose-dependent increase in reticulocytes	n/a	15.28	2	Guo et al. (2001)
*Chronic studies*								
B63CF mice, (50 m/dose)	Drinking water	100 weeks	0, 8.2, 41.2, 82.4 mg/kg bw/d	Sign. decreased water consumption at 77.8 mg/kg	82.4	n/a	1	DeAngelo et al. (1998)
Rats, no further details	n/a	2 years	28.22/36.2 mg/kg bw/d	No effects	28.22/36.2	n/a	n/a	Ford et al. (1959) as cited in Joint FAO/WHO Expert Committee on Food Additives (1964)
Mice, no further details	n/a	8 generations	1.95/2.51 mg/kg bw/d for males and females	No effects	1.95/2.51	n/a	n/a	Ford et al. (1959) as cited in Joint FAO/WHO Expert Committee on Food Additives (1964)
F344/N rats, (50 m/dose)	Drinking water	100 weeks	0, 1, 5.2, 10.4, 20.8 mg/kg bw/d	Increased mortality ≥ 10.4 mg/kg, bw decreased, kidney and testes weight increased at 20.8 mg/kg, urothelial hyperplasia ≥ 5.2 mg/kg	1	5.2	1	DeAngelo et al. (1998)
Wistar rats, m, no numbers given	Drinking water	15 months	30 mg Bromate/kg bw/d	Bw gain inhibited, necrotic changes in kidneys, increased blood urea nitrogen, structural abnormalities of the cortical tubules	n/a	30	n/a	Nakano et al. (1989) as cited in ECHA

**Table 8:** Carcinogenicity studies. For detailed information on the study quality (= tier) see Table 9

Species	Administration	Duration	Dosages	Observations	Tier [SciRAP]	References
F344 Rats, m (370 in total)	Drinking water	100 weeks	0, 1, 5.2, 10.4 or 20.8 mg/kg bw/d	Tumors in kidney, tunica vaginalis at 20.8 mg/kg bw/d, in thyroid gland at ≥ 5.2 mg/kg bw/d	2	Wolf et al. (1998)
F344 Rats (50 m/dose)	Drinking water	100 weeks	0, 1, 5.2, 10.4, 20.8 mg/kg bw/d	Tumors in tunica vaginalis testis at ≥ 5.2 mg/kg bw/d, in kidneys at 20.8 mg/kg, thyroid and mesothelium at ≥ 10.4 mg/kg	1	DeAngelo et al. (1998)
C6C3F Mice (50 m/dose)	Drinking water	100 weeks	0, 8.2, 41.2, 82.4 mg/kg bw/d	Tumors in kidney in top dose group	1	DeAngelo et al. (1998)
F344 Rats, (50/sex/dose)	Drinking water	110 weeks	0, 12.5, 27.7 mg/kg bw/d in males, 0, 12.5, 25.5 mg/kg bw/d in females	Tumors in kidney (m, f) at ≥ 12.5 mg/kg bw/d, and in peritoneum (m) at 27.7 mg/kg bw/d	2	DeAngelo et al. (1998)
F344 Rats (148 m in total)	Drinking water	104 weeks	0, 0.9, 1.7, 3.3, 7.3, 16 or 43.4 mg/kg bw/d	Tumors in kidney (≥ 7.3 mg/kg bw/d) and in thyroid and peritoneum (43.4 mg/kg bw/d)	1	Kurokawa et al. (1983)
F344 Rats (50/sex/dose)	Drinking water	110 weeks	0, 12.5, 27.7 mg/kg bw/d in males and 0, 12.5, 25.5 mg/kg bw/d in females	Tumors in kidney (mM), peritoneum (m) each at ≥ 12.5 mg/kg bw/d	1	Kurokawa et al. (1986a)
B6C3F Mice (50/sex/dose)	Drinking water	104 weeks	0, 56.5, 119.8 mg/kg bw/d	No significant different tumor incidences from the control group	1	Kurokawa et al. (1986b)
F344 rats (232 m in total)	Drinking water	13, 26, 39, 52 or 104 weeks	0, 26 mg/kg bw/d	First renal adenomas found after 26 weeks, renal adenocarcinoma after 104 weeks	2	Kurokawa et al. (1986b)
F344 Rats (81 m)	Single intragastric administration	87 weeks	0, 300, 600 mg/kg bw/d	Tumors in kidney (600 mg/kg bw/d)	2	Kurokawa et al. (1987)
B6C3F, BDF, CDF Mice (27 m/group)	Single intragastric administration	88 weeks	0, 77 mg/kg bw/d	Tumors in small intestine (CDF) and liver (B6C3F) at 77 mg/kg bw/d	n/a	Oinuma, 1974 as cited in Kurokawa et al. (1990)

**Table 9:**
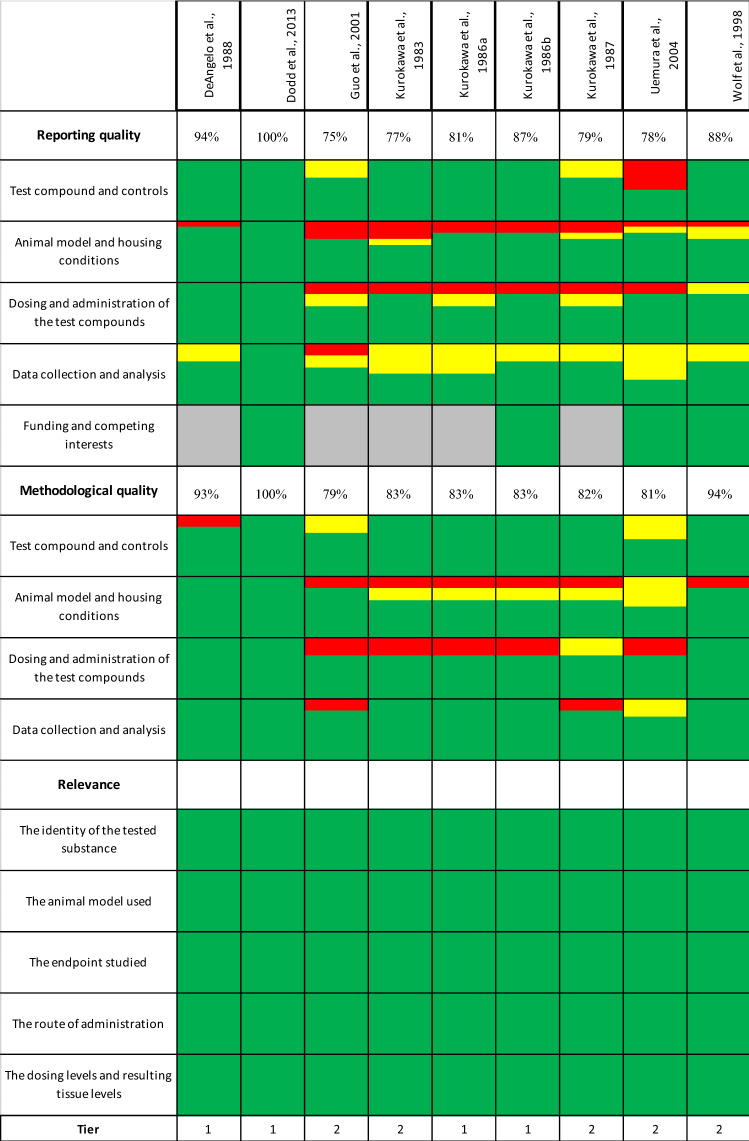
Quality of animal key studies (Tables 7 and 8) estimated by means of SciRAP

A detailed evaluation can be found in the supplementary data. Percentages for reporting and methodological quality indicate completeness of data. The lower the tier (last row) the better the study quality

#### Acute toxicity studies

Potassium bromate was given to F344 rats, B6C3F mice and Syrian golden hamsters (5/sex/group) as a single intragastric dose, with an observation period of 7 days. In all high dose groups (700–900 mg/kg bw), two thirds of the animals died within 3 h after administration, and the remaining animals died within 48 h. LD_50_ values were calculated to be 400/495 mg/kg for rats, 280/355 mg/kg for mice and 388/460 mg/kg for hamsters, each for male and female animals, respectively (Kurokawa et al. 1990). In further acute toxicity studies, potassium bromate induced oxidative stress and impaired the antioxidant capabilities in male Wistar rats. The animals were given a single oral dose of 100 mg/kg bw potassium bromate and they were sacrificed 12, 24, 48, 96 and 168 h after treatment. Some changes in blood parameters were observed, indicating the presence of oxidative stress. These effects peaked 48 h after administration, after which recovery took place (Ahmad and Mahmood 2012). In a further study, a single dose of 100 mg/kg bw in male Wistar rats elicited deleterious nephrotoxic effects, including increased reactive oxygen species (ROS) production and induced oxidative stress. After 48 h, the symptoms were reversible (Ahmad et al. 2012). Hassan et al. (2019) treated Swiss albino rats with a single dose of potassium bromate at the sub-lethal dose of 100 mg/kg bw. Extensive toxic effects were reported, such as altered liver function markers, influenced redox status and severe damage of liver tissues characterized by stained granules and vacuoles and a dilated central vein. However, the route of exposure was not stated.

To investigate the occurrence of oxidative stress after potassium bromate exposure, female F344 rats were exposed to 300 mg/kg by a single intragastric injection or to 80 mg/kg by a single intraperitoneal (i.p.) injection. The animals were sacrificed 48 h after administration and 8-oxodeoxyguanosine (8-oxodG) levels in the kidney were measured. The levels were significantly increased when compared to the control groups (Umemura et al. 2004). There were indications of bromate-induced ototoxicity with the cochlea being the primary site of lesion, which might cause irreversible sensorineural hearing loss with unknown incidences. Data from acute exposure studies show corresponding effects at high doses primarily in guinea pigs (Campbell 2006).

The results of these studies indicate that the administration of single sublethal doses of potassium bromate down to 100 mg/kg bw elicited deleterious nephrotoxic and hepatotoxic effects, as well as increased oxidative stress in rats, with some effects being transient. Male mice are the most susceptible sex/species for acute effects, with the lowest LD50 value of 280/355 mg/kg bw for male and female mice, respectively (Kurokawa et al. 1990).

#### Subacute and subchronic studies

An overview of the subacute, subchronic and chronic animal studies except carcinogenicity studies is given in Table 7.

Factors for converting concentrations of substances in drinking water into a daily dose for rats and mice for subacute, subchronic and chronic study durations were taken from EFSA Scientific Committee (2012).

There is no evidence for central nervous system malformations or brain weight changes in developmental studies (Crofton 2006). However, as in the acute toxicity studies, data from subacute exposure studies showed corresponding effects at high doses in guinea pigs, whereas ototoxicity was the most sensitive effect for which no lowest dose has been established, particularly for long-term low-dose exposure (Campbell 2006).

When exposed to potassium bromate, groups of 10 male and female F344 rats were given potassium bromate for 13 weeks at doses of 150, 300, 600, 1250, 2500, 5000 and 10,000 ppm (13.35/13.95, 26.7/27.9, 53.4/55.8, 111.25/116.25, 222.5/232.5, 445/465, 890/930 mg/kg bw/d for males and females, respectively) via drinking water. All animals given ≥ 2500 ppm died within 7 weeks, body weight gain was decreased in male rats given 600 and 1250 ppm, and significant changes in body weight and clinical chemistry were observed in rats of both sexes at 600 ppm, as well as extensive regenerative changes in the kidneys (Kurokawa et al. 1990). However, the primary literature is not available and the description of the study lacks detailed specifications. A NOAEL was not explicitly given but can be determined to be 300 ppm (26.7/27.9 mg/kg bw/d) from the data provided in the study (Kurokawa et al. 1990).

To further investigate renal effects, male F344 rats were orally exposed via drinking water to 600 ppm (53.4/55.8 mg/kg bw/d for males and females, respectively) potassium bromate for 12 weeks. Four weeks after the initial treatment, eosinophilic bodies were found in renal tubules. However, these deposits were transient and normalized after treatment (Kurokawa et al. 1990). In the following studies, precise information on dosages was not provided (Kurokawa et al. 1990); therefore, no information on doses per kg bodyweight can be given. Rats, dogs and monkeys were fed with bread made from flour treated with potassium bromate. Rats were exposed to flour with 14 and 100 ppm over a period of 10 months and three generations. Histology revealed no pathological alterations, no alterations in reproductive performance and no accumulation of bromide in the tissues. Rats treated with flour containing potassium bromate at levels of ~ 75 ppm for four weeks showed no abnormalities and reproductive performance was comparable with controls. Animals treated with flour containing potassium bromate at 200 ppm for 10 weeks also showed no adverse effects. Three dogs fed with bread made from flour containing up to 200 ppm for 16 days or 76 ppm for 12 weeks and showed no adverse effects.

In addition, three monkeys exposed for 8 weeks to a diet containing 84% bread made from flour containing 75 ppm potassium bromate showed no adverse effects (Ford et al. 1959 as cited in Joint FAO/WHO Expert Committee on Food Additives 1964; Kurokawa et al. 1990).

Male Swiss mice orally exposed to 2 g/L (348 mg/kg bw/d) potassium bromate via drinking water for 2 weeks exhibited oxidative stress and protein oxidative damage in the liver. Elevated plasma transaminase levels, such as AST and ALT, also indicated liver damage, which was accompanied by histological changes e.g. hepatic steatosis, leucocyte infiltration, hepatocyte vacuolization and necrosis (Ben Saad et al. 2016). Swiss Webster mice exposed to up to 100 and 200 mg/kg bw/d for 42 days via gavage showed hematological changes, impaired renal and hepatic histology such as congested central veins and vacuoles and decreased antioxidant capacities (Altoom et al. 2018). Neurotoxic symptoms were described in a study by Ajarem et al. (2016), in which male albino mice were treated via gavage for 42 days with 100 and 200 mg/kg bw/d potassium bromate. All treated animals showed neurobehavioral changes, as well as decreased neurotransmitter levels. They also had reduced brain levels of GSH, accompanied by extensive damage according to the histological sections of the medulla and cerebral cortex of the brains.

Male F344 rats were exposed to potassium bromate via drinking water at 0, 5, 20, 100, 200 or 400 mg/L (0, 0.4, 1.6, 8.1, 16.5 or 34.9 mg/kg bw/d) for 2 or 13 weeks. Increased kidney weights were observed in the highest dose group after exposure for 2 and 13 weeks. In the renal tubules, hyaline droplets were observed at 200 and 400 mg/L after 2 weeks and at 400 mg/L after 13 weeks. For a treatment period of 13 weeks, the NOAEL was determined to be 100 mg/L (8.1 mg/kg bw/d (Dodd et al. 2013)).

To evaluate the immunotoxic potential in female B6C3F1 mice, sodium bromate was administered in the drinking water at doses of 80, 200, 400, 600 and 800 mg/L (15.28, 38.2, 76.4, 114.6, 152.8 mg/kg bw/d) for 28 days. Minimal toxicological and immunotoxic effects were observed. All treated animals revealed significantly increased absolute and relative spleen weights. Some hematological parameters such as MCH or MCHC were slightly decreased in the highest dose group. A dose-related increase in reticulocytes was observed. No further parameters were affected. The number of T cells, B cells, NK cells and macrophages were not altered in any dose group. The suppressive effect of macrophages on the proliferation of B16F10 tumor cells was decreased after exposure to sodium bromate (Guo et al. 2001).

Male and female F344 rats were exposed to 0, 15, 30, 60, 125, 250 or 500 mg/L (0, 1.88/1.82, 3.75/3.63, 7.5/7.26, 15.63/15.13, 31.25/30.25, 62.5/60.5 mg/kg bw/d, respectively) potassium bromate in the drinking water for 4 weeks. In both sexes, the 8-oxodG formation was significantly increased at 250 mg/L and above. Accumulation of α2-macroglobulin in the kidneys of male rats was significantly increased at 125 mg/L and above. These results suggest that DNA oxidation may occur independently of lipid peroxidation and higher levels than 250 mg/L of potassium bromate in the drinking water might exert carcinogenic effects by oxidative stress. The NOAEL was set to 60 mg/L (7.5/7.26 mg/kg bw/d) (Umemura et al. 2004).

In conclusion, potassium bromate was shown to be toxic in repeated dose studies in rats and mice; main targets were kidney, liver, brain and clinical chemistry parameters. The lowest LOAEL of all subchronic and subacute studies was reported in the study of Umemura et al. (2004), with a value of 15.6/15.13 mg/kg bw/d and a corresponding NOAEL of 7.5/7.26 mg/kg bw/d for males and female rats, respectively. At the LOAEL, the kidney was affected, evident as an accumulation of α2u-globulin. These results were supported by the study of Dodd et al. (2013), in which the kidneys of treated animals were affected, with a LOAEL of 16.5 mg/kg bw/d and a NOAEL of 8.1 mg/kg bw/d. However, according to Umemura et al. (2004) and Dodd et al. (2013), male rats were more susceptible to bromate exposure than female rats and rats represented the more sensitive species compared to mice. Therefore, the studies showing the most sensitive results were performed in male rats. Both studies were well performed and, according to the SCiRAP evaluation, the study of Dodd et al. (2013), showed no deficits at all.

#### Chronic and long-term studies

Mice fed flour containing 15 ppm (1.95/2.51 mg/kg bw/d for males and females) potassium bromate showed no adverse effects over eight generations. Likewise, rats fed for 2 years with flour containing 627 ppm (28.22/36.2 mg/kg bw/d) potassium bromate also showed no significant abnormalities when compared to the controls. However, the description of the study lacks many details and specifications. For example, the sex of the mice is not stated and the dosing regimen and scope of investigation is unclear. Therefore, this study has limited relevance for use in an evaluation (DIN 19643-1:2012-11 2012; Ford et al. 1959 as cited in Joint FAO/WHO Expert Committee on Food Additives (1964)).

In another study, male Wistar rats were exposed to 0.4 g/L (30 mg/kg bw/d) potassium bromate via drinking water for up to 15 months. Body weight gain was markedly inhibited in the treated animals. After 7–11 weeks, histological examination of the kidneys revealed karyopyknotic foci (necrotic changes) in tubules of the inner medulla. At the end of the exposure period, hematology revealed increased blood urea nitrogen and structural abnormalities of the cortical tubules. The LOAEL was set to 30 mg/kg bw/d but no NOAEL was determined (Nakano et al. 1989; as cited in US EPA 2001)).

In a further study, male B6C3F mice were treated with 0, 0.08, 0.4 or 0.8 g/L (0, 8.2, 41.2, 82.4 mg/kg bw/d) for up to 100 weeks and male F344 rats were treated with 0, 0.02, 0.1, 0.2 or 0.4 g/L (0, 1, 5.2, 10.4, 20.8 mg/kg bw/d) via drinking water. In mice, there were no significant differences in survival rate, body weight gain, feed consumption or organ weight observed in any dose group. The only effect observed was significantly decreased water consumption in the top dose group. By contrast, rats exhibited an increased mortality rate at doses of 0.2 and 0.4 g/L. The final mean body weight was significantly decreased; kidney and thyroid weights were significantly increased in the top dose group. Treated rats developed foci of mineralization of the renal papilla, as well as eosinophilic droplets in the proximal tubule epithelium. A dose-dependent increase in urothelial hyperplasia was observed in the renal pelvis at 0.1 g/L and above. Therefore, the NOAEL for non-neoplastic observations in male rats was set at 0.02 g/L (1 mg/kg bw/d) (DeAngelo et al. 1998).

Taken together, long-term studies up to 100 weeks were performed in mice and rats. Since it has been reported that males are more susceptible to bromate-induced effects, only male animals were used in these experiments. A LOAEL was determined in male rats to be 5.2 mg/kg bw/d, with a corresponding NOAEL of 1 mg/kg bw/d (DeAngelo et al. 1998). Adverse effects included the urogenital system, with urothelial hyperplasia observed at the LOAEL, while the testes and kidneys were affected at higher dosages. According to the evaluation by SciRAP, this study was of a high quality. In the study of Narkano et al. (1989), as cited in US EPA (2001)), the urogenital system was also affected, with necrotic changes of the kidneys and structural abnormalities of the cortical tubules in male rats. However, only a single dose of 30 mg bromate/kg bw/d was reported for this study, which is higher than in the study of DeAngelo et al. In the latter study, male mice exhibited no effects up to the highest dose of 82.4 mg/kg bw/d. Therefore, rats clearly seem to be the more susceptible species for chronic effects.

In summary, animal studies for non-cancer effects indicate the urogenital system, especially the kidneys, to be the major target of adverse effects after acute, subchronic and chronic exposure of bromate. Rats appeared to be the more susceptible species, with a NOAEL of 1 mg/kg bw/d via drinking water in the study of (DeAngelo et al. 1998), although there are conflicting results for acute toxicity with LD50 values in mice being reported to be lower than in rats. According to literature data, males are more susceptible than females; therefore, most of the studies were performed in males.

#### Carcinogenicity studies

The carcinogenicity of bromate was evaluated in various animal studies, mostly in standard 2-year studies with rats. An overview of the following studies is given in Table 8.

Groups of 53 male and 53 female F344 rats were treated with potassium bromate via drinking water at 0, 250 or 500 ppm (0, 12.5, 27.7 mg/kg bw/d in males, 0, 12.5, 25.5 mg/kg bw/d in females) for 110 weeks. At the top dose, significant signs of general toxicity and increased mortality were observed. At 250 and 500 ppm, the incidence of renal cell tumors was significantly increased in both sexes. Mesotheliomas of the peritoneum were increased in male rats in a dose-dependent manner and were significant at 500 ppm (Kurokawa et al. 1983). According to the additional publication of this experiment, the top dose was decreased after week 60 to 400 ppm due to marked decreases in body weight gain (Kurokawa et al. 1986b). There was an increased mortality of male rats in the top dose group. In rats of both sexes, there were incidences of renal cell tumors and, in males, incidences of mesotheliomas of the peritoneum were significantly increased when compared to the control group. In one study with B6C3F mice (50 animals/sex/dose), doses of 500 and 1000 ppm (0, 56.5, 119.8 mg/kg bw/d) were administered via drinking water over 78 weeks (Kurokawa et al. 1986b). There was no significant difference in the incidence of tumors compared to the control group. The mice were sacrificed in week 104. In the top dose group, body weight gain was decreased but no increased mortality was observed in any dose group. Treatment of male mice was, however, discontinued due to highly aggressive behavior.

In 1986, the same authors performed a carcinogenicity study of potassium bromate in 148 male F344 rats divided into 7 groups treated with 0, 15, 30, 60, 125, 250 or 500 ppm (0, 0.9, 1.7, 3.3, 7.3, 16 or 43.4 mg/kg bw/d) via drinking water over a period of 104 weeks. In the top dose group, decreased body weight gain and increased mortality was observed. Combined incidences of renal adenocarcinomas and adenomas were significantly and dose-dependently increased at 125 ppm and above. In the highest dose group, the combined incidences of follicular adenocarcinomas, adenomas of the thyroid, and mesotheliomas of the peritoneum were significantly increased. At 60 ppm and above, dysplastic foci of the kidneys were significantly also increased. These lesions were considered to be pre-neoplastic effects, which gradually develop into neoplasms (Kurokawa et al. 1986a).

The carcinogenicity of potassium bromate was also studied in male B6C3F mice and male F344/N rats. Mice were treated with 0, 0.08, 0.4 or 0.8 g/L (0, 8.2, 41.2, 82.4 mg/kg bw/d) for up to 100 weeks and rats were treated with 0, 0.02, 0.1, 0.2 or 0.4 g/L (0, 1, 5.2, 10.4, 20.8 mg/kg bw/d) via drinking water. A dose-dependent increase in the incidence of mesotheliomas of the tunica vaginalis testis was observed, which was not statistically significant in the lowest dose group but was considered biologically significant. Rats developed renal cell tumors at concentrations of 0.1 g/L and above, which were significantly increased only in the top dose group. Thyroid follicular proliferative lesions, including hyperplasia, adenoma and carcinoma, were observed at 0.02 g/L and above but were significantly increased only in the two top dose groups. The same renal cell tumor observed in rats was also significantly increased in all treated male mice without any dose dependency. There were no other treatment-related increases in benign or malignant neoplasms in any of the examined tissues. However, mesotheliomas of the tunica vaginalis are of questionable relevance for humans and the incidence in the lowest dose group was not significant (DeAngelo et al. 1998).

In a re-evaluation of the rat study by DeAngelo et al. (1998), the time course of the carcinogenic effects of potassium bromate were discussed (Wolf et al. 1998). In addition to the final examination after 100 weeks reported by DeAngelo et al., data on 120 male F344 rats examined after 12, 26, 52, and 78 weeks were reported. Six animals from each group were sacrificed and necropsied at each interim time point. After 52 weeks, renal cell tumors were observed in the top dose group only. Mesotheliomas of the tunica vaginalis were observed after 52 weeks of treatment in both of the highest dose groups. After 78 weeks, mesothelioma were also present in other sites in a decreasing manner in spleen, gastrointestinal tract, mesentery, pancreas, urinary bladder, liver, and rarely in kidney. The incidence of thyroid follicular tumors was increased after 26 weeks at 0.1 g/L but not in a dose-dependent manner. After 100 weeks, even in the lowest dose group of 0.02 g/L, a significantly increased incidence of thyroid tumors was observed. All incidences and numbers of animals for the terminal examination were identical between DeAngelo et al. and Wolf et al., the only discrepancy was the reported incidence of thyroid tumors, for which no explanation was given by Wolf et al. (1998).

In another study, 81 male Fisher 344 rats were treated with a single intragastric administration of 0, 300 and 600 mg/kg potassium bromate and then observed for a period of 87 weeks. In the top dose group, 13.6% of the animals developed renal tumors. By contrast, there were no such tumors in the low dose and control group (Oinuma 1974 as cited in Kurokawa et al. (1990)).

Groups of 27 male mice of B6C3F, BDF and CDF strains treated orally with 0 and 750 ppm (0 and 77 mg/kg bw/d) for 88 weeks revealed no statistically significant differences in growth rate or survival time between the treated and control groups. However, significantly increased incidences of adenoma of the small intestine in CDF mice and significantly increased incidences of adenoma of the liver in B6C3F mice occurred when treated with 750 ppm. Nevertheless, there were no significant differences in the occurrence of adenocarcinoma between treated and control groups (Oinuma 1974 as cited in Kurokawa et al. (1990)). This single dose study is not suitable for a proper assessment of carcinogenicity.

Carcinogenicity of potassium bromate was primarily investigated in Fischer F344 rats, with a few studies being performed in mice. The test substance was predominantly administered via drinking water. In these studies, potassium bromate was shown to be a carcinogen in the rat (kidney, thyroid gland, mesothelium, peritoneum and tunica vaginalis testis) and in mice. However, the mouse studies showed an unclear pattern. In one of the three studies, the kidneys of C6C3F mice were affected at doses of 77.8 mg/kg bw/d (DeAngelo et al. 1998). Kurokawa et al. (1986b) could not find any tumors up to 119.8 mg/kg bw/d in B6C3F mice and Oinuma (1974, as cited in Kurokawa et al. (1990)) reported increased incidences of tumors of the intestine (CDF mice) and the liver (B6C3F mice) at the lowest dose of 60 mg/kg bw/d. According to Umemura and Kurokawa (2006), male rats are the most susceptible species for bromate exposure and only kidney tumors were found in both sexes, while the other tumors were restricted to male animals.

Taken together, studies with male rats were considered as most suitable for the derivation of guidance values. The types of tumors are discussed below.

## Modes of action

The toxicity of bromate is related to different MOAs. Generally, genotoxic and non-genotoxic mechanisms can be distinguished from each other. For genotoxicity, mutagenic and clastogenic effects are discussed in the following sections. The non-genotoxic MOAs related to bromate include thyroid and sex hormone imbalance, oxidative stress and alterations in apoptosis. Further references for potential cellular MOAs come from toxicogenomic analyses of potassium bromate. Global transcription analyses of human intestinal epithelial cells treated with 0.42 mM potassium bromate (IC_10_ value) results in 370 differently expressed genes (DEGs with *p* ≤ 0:01 and fold change (FC) ≥ 1:2). Biological context analyses using pathway enrichment revealed significantly affected gene ontology (GO) terms in 10 functional categories: inflammation and immune response, cell cycle, cellular processes, chemotaxis, signal transduction, DNA damage and oxidative stress (Procházka et al. 2019), which all reflect possible modes of action.

### Genotoxicity

Genotoxicity of bromate was studied in several in vitro and in vivo systems. This included a wide range of studies employing genotoxicity core tests such as micronuclei (MN) formation, sister chromatid exchange (SCE) and chromosomal aberrations (CA). Furthermore, mutagenicity studies were performed in bacteria, cellular systems, as well as in rodents. Indicator tests, such as analyses of oxidative DNA lesions (i.e., mostly 8-oxo-dG) and of DNA strand breaks, serve as biomarkers of exposure and can give additional information on the MOA. An overview of parameters and results of selected studies is given in Table S1 and will be discussed in the following paragraph. A limitation is that none of the studies identified in this publication were conducted according to OECD guidelines. Furthermore, most of the studies fall into category 3 according to Klimisch criteria (Klimisch et al. 1997), mostly because of missing information on the purity of the used bromate or the lack of a positive control in core tests. Where such information was available, studies fell into Klimisch categories 2 or 3. However, it is considered likely that most of the studies that did not report purity of the used bromate nevertheless used chemicals of high purity. Considering the wide range of studies, model systems and endpoints analyzed, in their entirety they give strong evidence for a genotoxic action of bromate without a clear No Observed Genotoxic Effect Level (NOGEL) in the low dose level (see Table S1 In vivo and in vitro data on genotoxicity).

#### Core tests

##### Clastogenicity and aneuploidy: analysis of micronuclei formation and chromosomal aberrations

The clastogenic and/or aneugenic properties of bromate were studied in a number of in vitro and in vivo models, in part in parallel to those in which primary DNA damage was analyzed, as reviewed below. Thus, in vitro micronucleus tests were reported in AS52 CHO cells (Ballmaier and Epe 2006), primary cultures of human and rat kidney cells (Robbiano et al. 1999), HepG2 cells (Zhang et al. 2011), human peripheral lymphocytes (Kaya and Topaktaş 2007), and human lymphoblastoid cells (Luan et al. 2007; Platel et al. 2009; Seager et al. 2012). Specifically, Robbiano et al. reported significant induction of micronuclei (MN) in primary rat and human kidney cells at a concentration of 0.56 mM bromate (Robbiano et al. 1999), a concentration that was also found to induce MN in human TK6 cells (Luan et al. 2007). With regards to studies in human cells, Kaya and Topaktas incubated human peripheral blood lymphocytes with 400–550 µg/mL potassium bromate for 24 and 48 h. MN were induced in a concentration-dependent manner at both time points analyzed. The authors also found evidence of increased SCE frequencies and chromosomal aberrations (Kaya and Topaktaş 2007). Of note, potassium bromate-induced the same level of chromosomal aberrations as the positive control, MMC. Platel et al. analyzed MN formation in TK6 cells over a concentration range of 50 µM to 5 mM using different treatment schedules for up to 24 h. Although the authors found some evidence of a threshold concentration of potassium bromate under certain treatment conditions in cells incubated for 24 h, the trend of increase in MN started from 50 µM with significantly increased values at 500 µM and higher. Similarly, Zhang et al. (2011) observed a significant increase in MN formation after treating HepG2 cells with concentrations ≥ 120 µM for 24 h. In another study that analyzed MN formation in human lymphoblastoid AHH1 cells in a low-dose range of 100–800 µM potassium bromate, there was a trend increase in MN at concentrations above 300 µM after a 4-h treatment with potassium bromate followed by a 22-h treatment with cytochalasin B (Seager et al. 2012).

In vivo studies on MN induction were performed in murine red blood cells (Awogi et al. 1992; Allen et al. 2000), bone marrow cells (Dongmei et al. 2015) and spermatids (Allen et al. 2000), as well as in rat red blood cells (Sai et al. 1992a), kidney (Robbiano et al. 1999), liver, stomach, colon and bone marrow cells (Okada et al. 2015). Most of these studies detected significant increases in MN formation in the cell types analyzed. The exceptions included a study using mouse bone marrow cells (Dongmei et al. 2015) and another study by Okada et al., which reported increased MN frequencies in rat stomach and bone marrow but not in liver and colon (Okada et al. 2015). A long-term and low-dose study analyzing male mice treated with ≥ 80 ppm (80 mg/l) potassium bromate in drinking water for 8 and 78 weeks found a significant increase in MN frequencies in erythrocytes after 8 weeks but not in spermatids at the lowest dose tested (Allen et al. 2000). The lowest dose of potassium bromate applied in a single treatment study in mice was 18.8 mg/kg bw (i.p. injection) followed by a 24- to 96-h observation period. Significant induction of MN was observed at 37.5 mg/kg bw after 24 h. No significant induction was evident at the lowest tested concentration of 18.8 mg/kg bw (Awogi et al. 1992).

Sai et al. showed that several antioxidants, such as GSH, Cys, and liposome-coated superoxide effectively prevented MN formation induced by potassium bromate in F344 rat peripheral blood cells (Sai et al. 1992a). The protective role of GSH is probably based on its ability to compete with DNA for reaction with free bromine radicals (Platel et al. 2009).

In conclusion, there is very solid evidence that bromate induces clastogenicity and/or aneuploidy in vitro and in/ex vivo in human and animal cells. It should be noted that most of the studies discussed above do not allow a clear discrimination between clastogenic and aneugenic effects. However, considering the fact that bromate induces oxidative DNA lesions and secondary strand breaks (see below), a clastogenic potential of bromate is very likely. Nevertheless, an aneugenic potential cannot completely be excluded, and may be possible through bromate-induced damage of proteins involved in the regulation of the spindle apparatus. Although there are some indications for a threshold dose/concentration for such effects, the available data do not allow deriving a threshold value for the induction of clastogenicity and aneuploidy according to our assessment.

##### Mutagenicity studies

Potassium bromate has been reported to be mutagenic at a dose of 3 mg potassium bromate per plate in the Salmonella reverse mutation assay (Ames test) (Ishidate et al. 1984), while in another study, no mutagenic activity of potassium bromate was observed at concentrations up to 600 µg per plate (Dongmei et al. 2015).

Several mutagenicity studies have been performed in cellular systems analyzing mutation frequencies in the Hprt, Tk, or Gpt loci in CHO cells (Speit et al. 1999; Ballmaier and Epe 2006), mouse lymphoma L5178Y cells (Harrington-Brock et al. 2003; Priestley et al. 2010), and human lymphoblastoid cells (Luan et al. 2007; Platel et al. 2011; Seager et al. 2012). All of these studies reported significant increases in mutation frequencies induced by potassium bromate, with the most sensitive response observed by Seager et al., who reported visible increases in mutation frequencies at a concentration of 250 µM potassium bromate after a 24-h treatment (POD in Hockey Stick model 180 µM) (Seager et al. 2012).

The in vivo, mutagenic activity of potassium bromate was analyzed in mice (Arai et al. 2002, 2003; Tsuchiya et al. 2018) and rats (Umemura et al. 2006, 2009; Yamaguchi et al. 2008). Significant increases in mutation frequencies were observed after treatment of animals with 500 ppm potassium bromate in drinking water for several weeks (Umemura et al. 2006; Yamaguchi et al. 2008). In both studies, lower concentrations did not result in significant increases in mutation frequencies.

In conclusion, there is convincing evidence from in vitro and in vivo studies using mammalian cells of a mutagenic potential of bromate. Similar to the results from clastogenicity and aneuploidy studies, the data do not allow the determination of a clear threshold value.

#### Indicator tests

##### Analysis of 8-oxo-dG

Levels of 8-oxo-dG as a biomarker of exposure were reported in multiple studies. The analyses were performed via (i) HPLC coupled to UV-detection, electrochemical detection or mass spectrometry, (ii) modified comet assays using the 8-oxo-dG-specific glycosylases FPG or OGG1, (iii) or immunochemical detection using antibodies raised against 8-oxo-dG.

Induction of oxidative DNA damage by bromate in cell-free systems, i.e. treatment of pure DNA, was studied by several groups (Ballmaier and Epe 1995, 2006; Parsons and Chipman 2000; Murata et al. 2001; Kawanishi and Murata 2006). The main type of damage induced by bromate was 8-oxo-dG adduct formation. By contrast, single strand breaks, AP sites, and other base modifications were only formed at minor frequencies (Ballmaier and Epe 1995). The presence of free thiols, such as GSH or other molecules with Cys residues, was necessary for direct damage induction (Murata et al. 2001). Experiments using deuterated water (D2O), as well as different scavenging reagents, such as catalase, SOD, desferoxamine, azide, and tert-butanol, excluded the involvement of hydroxyl radicals or singlet oxygen as the main DNA reactive molecules (Ballmaier and Epe 1995). Instead, the hydroxyl radical scavenger, methional, inhibited the formation of 8-oxo-dG efficiently. Methional not only scavenges hydroxyl radicals but also chemical species with weaker reactivity (Murata et al. 2001). Furthermore, enzymatic reaction or the presence of transition metals were not required for the generation of bromate-induced DNA damage (Ballmaier and Epe 2006). Taken together, these studies led to the conclusion that neither molecular bromine nor ROS are the main DNA-reactive molecules under cell-free conditions. Instead, bromine radicals or oxides appear to be the relevant DNA damaging species (Murata et al. 2001; Ballmaier and Epe 2006). Thus, it is possible that GSH/Cys reduces bromate to BrO2, which in turn oxidizes guanine. Similarly, GSH/Cys can reduce BrO2- and BrO- to BrO and Br*, which may also react with DNA (Murata et al. 2001).

Oxidative DNA damage as a primary marker for DNA damage has been analyzed at various concentrations and treatment schedules in a wide spectrum of cellular models, such as in primary rat kidney cells (Sai et al. 1994; Parsons and Chipman 2000), L1210 mouse leukemia cells and LLC-PK1 porcine kidney cells (Ballmaier and Epe 1995), mouse lymphoma L5178Y cells (Priestley et al. 2010), V79 Chinese hamster lung cells (Speit et al. 1999), AS52 Chinese hamster ovary cells (Ballmaier and Epe 2006), human leukemia HL60 cells (Murata et al. 2001), TK6 human lymphoblastoid cells (Platel et al. 2011), and human HepG2 liver-derived cells (Zhang et al. 2011). Specifically, Sai et al. reported an increase in 8-oxo-dG in rat renal proximal tubule cells after treatment with bromate at ≥ 2 mM and suggested that lipid peroxidation may be involved in the generation of oxidized DNA damage (Sai et al. 1994). Ballmaier and Epe compared damage profiles and 8-oxo-dG levels in L1210 mouse leukemia cells and LLC-PK1 porcine kidney cells after treatment with millimolar concentrations of bromate. The authors observed damage profiles similar to those observed in isolated DNA. In LLC-PK1 cells, which as kidney cells are derived from the target organ of bromate carcinogenicity, 8-oxo-dG levels were twice as high as those in L1210 mouse leukemia cells, which are derived from a non-target organ. However, a straightforward interpretation of this finding appears difficult due to different species origin. Remarkably, under conditions that did not influence cell proliferation, Fpg-sensitive base modifications were quite persistent and were repaired only with moderate efficiency (Ballmaier and Epe 1995). With regard to dose–response and time-course analyses of DNA damage induction, Parson and Chipman reported significant increases in 8-oxo-dG after treating rat kidney epithelial cells with 1.5 mM potassium bromate for 24 h. No increase was observed after short-term treatment of 15 min (Parsons and Chipman 2000). By contrast, Murata et al. observed a significant induction of 8-oxo-dG in human leukemia HL60 cells already 4 h after treatment with ≥ 0.5 mM potassium bromate (Murata et al. 2001). Similarly, Priestly et al. reported significant increases of 8-oxo-dG in mouse lymphoma L5178Y cells already 3 h after treatment at a concentration of 0.5 mM potassium bromate (Priestley et al. 2010). In the latter study, significant removal of 8-oxo-dG lesions was observed after 21 h; however, it is not completely clear if this effect was due to repair or dilution of lesions as a consequences of cell proliferation. In a recent study, using a modified FADU assay, significant induction of Fpg-sensitive sites was observed after treatment of human THP1 macrophage-derived cells for 2 h already at concentrations ≥ 50 µM potassium bromate (Mack et al. 2021).

Consistent with results from cell-free studies, depleting GSH/Cys levels in cells using diethylmaleate (DEM) led to a reduction in DNA damage levels (Ballmaier and Epe 1995; Parsons and Chipman 2000), suggesting a role of free thiols in DNA damage formation also in cellular systems. Such an effect was, however, not observed in in vivo studies (Sai et al. 1992b; Chipman et al. 1998), suggesting different mechanisms of DNA damage formation in whole organisms. In general, a multitude of in vivo studies in mice and rats demonstrated the formation of 8-oxo-dG in several organs after potassium bromate treatment. Studies in rats were performed by Kasai et al. 1987; Sai et al. 1991; Umemura et al. 1995; Chipman et al. 1998; Umemura et al. 1998; Cadenas and Barja 1999; Umemura et al. 2004; Umemura et al. 2009; Kolisetty et al. 2013. There was a significant induction of 8-oxo-dG after single dose i.p. injections at doses of 40–100 mg/kg bw (Sai et al. 1991; Chipman et al. 1998; Cadenas and Barja 1999). There was no increase compared to background levels at 20 mg/kg bw (Sai et al. 1991). When administered via drinking water, increased levels of 8-oxo-dG were observed in kidneys of F344 rats treated for 28 days with bromate doses as low as 46 mg/L in (Kolisetty et al. 2013). However, in most studies, bromate concentrations applied by drinking water were higher, i.e., in the range of 250–2000 mg/L (Umemura et al. 1998, 2004, 2009). Time-course studies demonstrated an increase in 8-oxo-dG levels in kidneys within 24 h after i.p. injections of potassium bromate, followed by a gradual decrease (up to 96 h) (Kasai et al. 1987; Sai et al. 1991). When administered in drinking water (500 mg/L), Umemura et al. observed an increase in 8-oxo-dG levels in male rats within 1 week after the onset of treatment, with levels remaining high during the entire period of bromate exposure. By contrast, in females increases in 8-oxo-dG levels occurred only 3 weeks after the first application of bromate (Umemura et al. 1998). Interestingly, GSH levels were reported to be higher in male than in female rats (Igarashi et al. 1983). Chipman et al. observed a significant twofold increase in in 8-oxo-dG in genomic rat kidney DNA after i.p. injection of bromate (100 mg/kg bw) and a 57% increase in DNA isolated from kidney mitochondria (Chipman et al. 1998). Interestingly, increases in 8-oxo-dG levels were usually higher in kidney than in liver tissue, which is a non-target tissue with regard to bromate-induced carcinogenicity (Kasai et al. 1987; Umemura et al. 1995). Probably as a compensatory mechanism to elevated 8-oxo-dG levels, Delker et al. observed an increase in Ogg1 mRNA expression in kidneys of F344 rats that were treated with 400 mg/L potassium bromate for up to 100 weeks (Delker et al. 2006). Of note, increases in oxidative DNA damage could be prevented or reduced by pre-administration of several antioxidants, such as resveratrol, melatonin, PBN, vitamin E (Cadenas and Barja 1999), as well as sodium ascorbic acid (Umemura et al. 2009). In addition to studies in rats, several drinking water studies were performed in wild-type and genetically modified mice (Arai et al. 2002, 2003, 2006). Increased levels of 8-oxo-dG were observed in kidney, liver and intestine of mice. Peak values of ~ 70-times above background were reported in Ogg1 KO mice treated with 2 g/L potassium bromate in drinking water for 13 weeks. In this study, 8-oxo-dG levels remained constant even 4 weeks after exposure to bromate was terminated. Even though mutation frequencies increased after bromate treatment, in particular in the Ogg1 deficient background (see below) (Arai et al. 2002, 2003), the authors did not find precancerous lesions in kidneys or any other organ after 12 weeks of treatment. In another mouse study, Yokoo et al. observed significantly lower levels of 8-oxo-dG formation in the intestines of Nrf2 KO mice (Yokoo et al. 2016). However, such a difference was not observed in murine kidneys, as reported by Tsuchiya et al. (Tsuchiya et al. 2018).

In conclusion, oxidative DNA damage, such as 8-oxo-dG lesions, appears to be a primary type of damage induced by bromate in vitro and in vivo. The available data support the view of a threshold dose response relationship for 8-oxo-dG formation (Spossova et al. 2015). However, at present, it is not entirely clear if this might be caused by limited sensitivity of the analytical methods used. Thus, it cannot be excluded that, in most of the above-mentioned studies, the reported lack of elevated 8-oxo-dG levels in the low-dose range was due to the induction of 8-oxo-dG below the limit of detection.

##### Analysis of DNA strand breaks

In addition to oxidative DNA damage, DNA strand breaks were analyzed following bromate treatment in a variety of cell culture models, such as primary rat and human kidney cells (Robbiano et al. 1999), V79 Chinese hamster cells (Speit et al. 1999), mouse lymphoma L5178Y cells (Priestley et al. 2010), human TK6 cells (Luan et al. 2007; Platel et al. 2011), isolated human white blood cells (Parsons and Chipman 2000), and human HepG2 cells (Zhang et al. 2011). Analyses were performed either with a standard alkaline comet assay, detecting DNA single and double strand breaks, as well as a neutral comet assay, preferentially detecting DNA double strand breaks. Some studies directly compared DNA strand break levels measured by the alkaline comet assay to those measured by the modified comet assay using Fpg and/or Ogg1 glycosylases to induce DNA strand breaks after excision of 8-oxo-dG (Speit et al. 1999; Priestley et al. 2010; Platel et al. 2011). Independent of the cell type tested, these studies consistently showed much a stronger increase in DNA strand break formation upon application of Fpg/Ogg1. These results are also consistent with a recent study, which demonstrated the absence of directly induced strand breaks by bromate up to a concentration of 200 µM, while Fpg-sensitive sites were detected already at 50 µM in human THP1 cells (Mack et al. 2021). Taken together, these studies again indicate that bromate mostly induces oxidative DNA damage, which can be converted to DNA strand breaks during base excision repair. This hypothesis is also supported by data from Zhang et al. (2011), which reported the absence of significant DNA strand breaks using the standard comet assay after bromate incubation with HepG2 cells for 40 min, while a significant and dose-dependent increase was obtained after incubation for 1 h, suggesting the occurrence of DNA strand breaks as DNA repair intermediates. At least one study reported the induction of DNA double strand breaks, as measured by the neutral comet assay, after 4 h incubation with 1 mM bromate (Luan et al. 2007). Consistent with the in vitro data from cell cultures, Ahmad et al. (2013) reported DNA strand breaks, analyzed using the alkaline comet assay, in intestines of adult male rats treated with a single oral dose of 100 mg/kg bw potassium bromate. Significant induction of DNA strand breaks was observed 12 h after treatment, with peak values reaching sixfold above background after 48 h. Thereafter, a decline was observed until the last measured time point of 168 h post treatment, suggesting progression of DNA repair.

In conclusion, the available data support the hypothesis that DNA strand breaks are formed as a result of bromate exposure in vitro and in vivo, mainly as secondary damage arising during the repair (or failed repair) of oxidative DNA lesions. Here, modeling of existing data suggests a linear dose–response relationship (Spassova et al. 2015).

#### General conclusions on the genotoxicity of bromate

As reviewed in the previous sections, there is strong evidence to consider bromate as a genotoxic compound. Bromate-induced clastogenic and aneugenic effects in various in vitro and in vivo assays. Moreover, mutagenic activity has also been demonstrated in vitro and in vivo*,* and a wealth of data proved oxidative DNA damage and DNA strand break formation upon bromate exposure. The situation becomes more complicated concerning questions, such as: (i) what is the exact underlying MOA of the genotoxicity of bromate, (ii) does a potential threshold dose exist and can it be quantitatively determined, and (iii) is the genotoxicity of bromate the sole contributing factor to its carcinogenic potential or do additional mechanisms play a role?

Concerning the MOA of the genotoxicity of bromate, thiol-dependent bromate reaction products appear to be a major source of primary DNA damage (Bull and Cottruvo 2006). Thus, it was shown in vitro that bromate-mediated oxidative DNA damage can be generated via thiol-dependent reaction products (Parsons and Chipman 2000; Murata et al. 2001; Ballmaier and Epe 2006). For example, Parson and Chipman 2000 concluded that extracellular GSH is protective against bromate-induced DNA damage, yet intracellular GSH actively mediates the genotoxicity of bromate. They assumed that GSH radicals might be involved in DNA damaging mechanisms. Thiols can react with bromate to sulfur-radicals which than can add on carbon–carbon double bonds. Moreover, an alternative and not mutually exclusive MOA for the observed genotoxicity was proposed by Kolisetty et al. (2013) who obtained evidence that bromate influenced apoptosis in the renal tubules in both male and female F344 rats. They hypothesized that suppression of apoptosis may lead to an induction of DNA damage.

Concerning the question if a potential threshold dose for the genotoxicity of bromate may exist, authors of some studies indeed proposed a thresholded dose–response relationship. However, in most cases, solid dose–response data, in particular in the low dose range, are not available. Furthermore, a series of statistical and modeling analyses of selected key genotoxicity studies was published and concluded that the dose–response relationships of bromate were also consistent with low-dose linear models of genotoxicity—at least for endpoints downstream of primary oxidative DNA lesions, i.e., DNA strand breaks, formation of MN and mutation frequencies (Spassova et al. 2013, 2015; Spassova 2019). The authors concluded that the data analyzed do not provide convincing evidence for the presence of a threshold for bromate genotoxicity. However, it should be noted that this conclusion may have been due to data limitations arising from the experimental studies. For example, the highest experimental concentrations not leading to detectable 8-oxo-dG induction originate from Umemura et al. (2006), who did not find a significant effect at 60 and 125 mg/L potassium bromate in drinking water in Gpt delta rats after 13 weeks of exposure. By contrast, the lowest exposure concentration to induce 8-oxo-dG adducts was reported by Kolisetty et al. (2013) who found a significant effect on 8-oxo-dG formation at a concentration of 46 mg/L bromate in drinking water in a 28-day study in F344 rats in both sexes. Interestingly, this roughly corresponds to the BMDL_10_ values derived for renal cancer in the same rat strain. Yet, in addition to genotoxicity, other mechanisms may also contribute to the carcinogenicity of bromate. In mice, the lowest BMDL_10_ reported for genotoxicity was 2.4 mg/L (i.e., for MN formation in mouse erythrocytes) (Health Canada 2018). This suggests that mice are more sensitive to genotoxicity induced by bromate than rats, while the carcinogenic potential of bromate in mice appears to be lower, which indicates that, in addition to genotoxicity, other parameters may contribute to the carcinogenic effects in rats (as discussed below).

Taken together, a threshold-like MOA for the genotoxicity of bromate may be possible; however, this cannot currently be assumed with reasonable certainty. In addition, the data do not allow a quantitative estimate of a potentially existing threshold dose. Therefore, we consider bromate as a genotoxic substance without a threshold-like dose–response relationship. At present, it is not completely clear to what extent bromate genotoxicity translates to carcinogenicity potential and if a potential threshold for carcinogenic effects exists (as discussed below).

### Non-genotoxic effects

As described by Health Canada (2018), other MOAs of the carcinogenicity of potassium bromate may exist. These MOAs include thyroid and sex hormone imbalance, immunosuppression and alterations of apoptosis (Health Canada 2018). Some recent findings of these MOAs will be discussed in the following paragraphs.

#### Thyroid hormone imbalance

The sodium iodide symporter (NIS) mediates the transport of iodide into thyroid epithelial cells and therefore accounts for one of the first steps in thyroid hormone synthesis. Both bromate and its stable metabolite, bromide, are substrates of NIS (Eskandari et al. 1997) and thus can be taken up by the thyroid. Consequently, damage by reactive intermediates or NIS inhibition followed by TSH stimulation were proposed as MOAs for the disruption of thyroid hormone homeostasis (Fisher and Bull 2006). Indeed, substrates of NIS, such as thiocyanate or nitrate, have been shown to competitively inhibit iodide uptake by NIS into the thyroid and to inhibit thyroid hormone synthesis. For bromate, significantly decreased T3 and T4 levels and significantly increased TSH levels were observed in male rats at a dose of 20 mg/kg bw given twice a week for four weeks (Khan 2017). Other studies, however, reported diverging effects of bromate on thyroid hormone synthesis. Whereas Wolf et al. (1998) reported decreased T3 levels at all doses investigated (0.02–0.4 g/L drinking water), but no effects on T4 concentrations after 12 weeks of treatment in rats, Dodd et al. (2013) reported unchanged T3 and T4 levels and a significant decrease of TSH only at 20 and 100 mg/L, but not at 5, 200 and 400 mg/L in a subchronic rat study.

Another proposed MOA of bromate in thyroids is direct oxidative damage. Rats administered potassium bromate at a dose of 110 mg/kg bw showed significant induction of lipid peroxidation in homogenates of thyroid glands (Karbownik et al. 2005). Lipid peroxidation accompanied by reduced activities of antioxidant enzymes e.g. catalase (CAT) and superoxide dismutase (SOD) as well as phase II metabolizing enzymes e.g. glutathione-S-transferase (GST) and glutathione reductase (GR), in thyroid tissue from rats was also observed in the study by Khan (2017). In vitro investigations using primary human thyroid cells from three donors treated with subtoxic potassium bromate concentrations from 1.25 to 5 mM showed a significant dose-dependent increase in the frequency of DNA single strand breaks and activation of DNA repair synthesis. In the same study, potassium bromate was administered to rats at a single dose corresponding to 1/2 LD50 which induced a statistically significant degree of DNA fragmentation in the thyroid (Mattioli et al. 2006). These data indicate a relevant uptake of bromate into the thyroid gland where the induction of oxidative stress leads to DNA damage.

In a 66 day study in which male rats were administered 10, 50 and 100 mg/L bromide (Velický et al. (1997)), T4 was decreased on day 16 and day 66 and T3 was decreased on day 66. The observed histopathological changes in the thyroid were in line with an activation of the hypothalamic-pituitary-thyroid axis at all dose levels. However, only a non-significant trend of a TSH increase at the high dose on day 66 was observed. Similarly, in male rats treated with 100, 200 and 400 mg/L for 133 days, histopathological changes in the thyroid suggested an activation of the hypothalamic-pituitary-thyroid axis. Further findings were a decrease of intrathyroidal iodide concentration, albeit without dose dependency, and decreased T4 levels. T3 concentrations in this study were unchanged. An increase in TSH was only observed at 100 mg/L, whereas only a mild decrease was observed at the higher doses (Velický et al. 1998).

Overall, there is evidence for a NIS-mediated uptake of bromate and bromide into the thyroid, although investigations on the disruption of thyroid hormone homeostasis or TSH stimulation by bromate led to conflicting results. The relevance of thyroid hormone homeostasis as a carcinogenic MOA in humans is, however, under discussion (see Sect. 6.1, Meek et al. 2003). Bromate and its metabolites were reported to cause DNA-strand breaks, as well as oxidative stress, in the thyroid gland, which may represent a possible MOA with respect to human relevance and a respective threshold.

#### Sex hormone imbalance

An altered balance of sex hormones can potentially result in promotion of tumors. Male rats treated for 4 weeks with 20 mg/kg bw potassium bromate showed reduced levels of FSH, LH and testosterone accompanied by reduced levels of antioxidant enzymes, e.g., catalase (CAT), peroxidase (POD), superoxide dismutase (SOD) and phase II metabolizing enzymes, e.g., glutathione reductase (GSR), glutathione peroxidase (GSHpx), GST and GSH (Khan et al. 2012). FSH enhances the production of an androgen-binding protein by the Sertoli cells, which plays an important role in the maintenance of spermatogenesis (Grover et al. 2005). A decrease in epididymal sperm density in potassium bromate-treated rats was observed in a reproductive study (Wolf and Kaiser (1996) as cited in (US EPA 2001)). LH stimulates testosterone production from Leydig cells. Leydig cell hyperplasia could also be a consequence of low testosterone. Leydig cell tumors were observed in rats exposed to potassium bromate, but also occurred in the control group (Health Canada 2018). Taken together, circumstantial evidence of a potential contribution of potassium bromate to sex hormone influenced carcinogenesis has been presented but further studies would be required for clarification.

#### Alterations in apoptosis

Sodium bromate has been suggested to increase apoptosis, which is followed by a compensatory suppression of apoptosis (Health Canada 2018). This alteration in apoptosis regulation is supported by in vitro data from human renal cells incubated with subtoxic concentrations of potassium bromate. This exposure led to a downregulation of TRAF3, NF-kB and IL1 gene expression, which counteract apoptosis and induce cellular dedifferentiation (Obaidi et al. 2018). Apoptosis suppression allows for survival and replication of cells with DNA damage, increasing the likelihood of renal tumor development (Bull and Cottruvo 2006). However, these mechanisms have not yet been confirmed in vivo.

## Derivation of the point of departure for risk assessment

The relevant and sensitive endpoints to be taken into account for human risk assessment are carcinogenicity and chronic renal toxicity as points of departure.

### Human relevance of the tumors observed in experimental animals

Potassium bromate was tested for carcinogenicity in hamsters, mice, and in rats. The clearest and most potent carcinogenic effect was evident from the studies performed in rats. All of the experiments in rats were carried out with the Fischer F344 strain. Potassium bromate-induced tumors in the kidney, the mesothelium, and the thyroid. Only kidney tumors were found in both sexes, the other tumors were restricted to male animals. One major site for the mesothelial tumors was the tunica vaginalis of the testis (DeAngelo et al. 1998). Tunica vaginalis mesotheliomas were found already after 52 weeks of treatment and were present at other mesothelial sites only after 78 weeks. This suggested that the primary tumor was the one located in the tunica vaginalis of the testis (Wolf et al. 1998; Crosby et al. 2000). In the kidney and thyroid, the specific tumor types induced were renal cell adenoma/carcinoma and thyroid follicular adenoma/adenocarcinoma, respectively.

For the derivation of human cancer risk estimates from bromate exposure, a decision on which of these tumors are appropriate for assessment is required. Thus, the tumor types induced by potassium bromate in the rat are discussed with regard to their relevance for humans.

Rat thyroid follicular tumors may be induced via several MOAs. One of them is considered not to be relevant for humans, namely UDP glucuronyltransferase (UGT) mediated induction in the absence of genotoxicity (IARC 1999; ECHA 2021). Furthermore, humans are generally considered to be less sensitive with respect to the induction of thyroid follicular tumors (IARC 1999). BMDL_10_ values for thyroid follicular tumors were derived using BMDS 3.1 and PROAST 67 using the data from (DeAngelo et al. 1998; Wolf et al. 1998) and (Kurokawa et al. 1983; Kurokawa et al. 1986a). The derived BMDL_10_ values ranged between 69.5 and 369 ppm (see supplementary files). The BMDL_10_ values for kidney cancer derived from the data were generally lower by a factor of up to sixfold (Table 10). Thus, if the thyroid follicular tumors would be used for risk assessment this would result in lower cancer risk estimates.

**Table 10:** Human cancer potency estimates for bromate based on F344 rat renal tumors derived by BMDS3.1 and PROAST 66.4 and 69 (for detailed results see supplementary data)

hBMDL_10_ [mg bromate/kg bw/d]^a^	Sex	Study	Model	Experimentally derived BMDL_10_ [mg/L concentration in rat drinking water]	AIC^c^	BMDU/BMDL
0.23	m	Kurokawa et al. (1983)	BMDS 3.1	23.6	138	3.7
0.16			PROAST 66.40	16.6	137	6.4
0.24	f	Kurokawa et al. (1983)	BMDS 3.1	25.4	125	3.3
(0.02)^b^			PROAST 66.40	1.86	125	36.2
0.65	m	Kurokawa et al. (1986a)	BMDS 3.1	67.7	90	3.0
0.66			PROAST 66.40	68.9	95	2.8
1.02	m	DeAngelo et al. (1998) and Wolf et al. (1998)	BMDS 3.1	107	128	2.6
0.85			PROAST 66.40/69	89.4	128	3.3
0.65		median, modelings above				

The hBMDL_10_ was derived by using an allometric factor of 4 and assuming a daily water consumption of 50 mL/kg rat (ECHA guidance R.8, EFSA 2012). A factor 0.765 correcting from potassium bromate to bromate (molecular weights 167 and 127.9, respectively) was applied. Calculating example: 23.6/4 × 0.765 /20 = 0.23. All BMDL_10_ values were obtained by model averaging

^a^To be used as point of departure for risk assessment

^b^Value excluded due to obviously outlying

^c^Rough value as no mean can be given

Tunica vaginalis mesotheliomas in rat testis were reviewed and evaluated for their relevance to humans (e.g. (Haber et al. 2009; Maronpot et al. 2009, 2016). This tumor type is found nearly exclusively in F344 rats. Mesothelial tumors are generally rare in female F344 rats. In other rat strains, tunica vaginalis mesotheliomas only appear occasionally and mainly after intraperitoneal application. Tunica vaginalis mesotheliomas are very rare in humans and were estimated to appear at a frequency of 2 × 10^–7^ (Haber et al. 2009). About a third of the cases were reported to be associated with asbestos exposure. With respect to this type of tumor induced by acrylamide, Haber et al. (2009) concluded that for risk assessment, a non-linear dose–response may be adequate due to an assumed non-genotoxic MOA. For tunica vaginalis mesotheliomas in rat testis, this would lead to very low risk estimates. Consequently, it is recommended not to use tunica vaginalis mesotheliomas in rat testis for extrapolation to derive human cancer risks. Considering Maronpot et al. (2016), tunica vaginalis mesotheliomas are most likely secondary to Leydig cell tumors, a common spontaneous tumor in Fischer rats. The key event might be hormone imbalance associated with Leydig cell tumors. Alternatively, mechanical pressure from Leydig cell tumors might implicate the generation of tunica vaginalis mesotheliomas. However, since in the respective rat study (DeAngelo et al 1998). Leydig cell tumors or pre-stages were not described, the mode of action of the observed tunica vaginalis mesotheliomas is unclear. Direct interaction via oxidative stress could be another mode of action plausible for bromate. Thus, human relevance cannot be entirely excluded. Since tunica vaginalis mesotheliomas in testis is very rare in humans and rats may be more sensitive with respect to tunica vaginalis mesothelioma development, this effect is not used as a basis for the BMDL derivation.

Renal cell adenoma and carcinoma induced in male rats caused by protein droplet accumulation containing α2u-globulin in tubule cells are known to be species-specific in the male rat and should not be used for extrapolation to humans (IARC 1999; ECHA 2021). However, in the case of bromate, the issue is more complex. On the one hand, several studies are available showing that there is clear evidence for α2-macroglobulin protein droplet accumulation in tubule cells in potassium bromate-treated male but not in female rats (for review, see (Health Canada 2018)). This would support lack of human relevance of the renal tumors found in male rats. On the other hand, positive genotoxicity (as evident for bromate) is one reason to assign a male renal cell tumor as human-relevant. Renal cell tumors were also induced in female rats (Kurokawa et al. 1983) indicating that another MOA was involved. The tumor incidences were at mostly only slightly higher in male than in female rats in the study performed by Kurokawa et al. (1983). Thus, the MOA of renal tumor induction via the α2-macroglobulin protein droplet accumulation pathway does not seem to be applicable to bromate. It is considered adequate to use the renal cell adenoma and carcinoma to derive human cancer risk estimates for bromate. This approach was also used for risk evaluation in other studies (Anses 2012; RIVM 2014; ECHA 2017a).

### Threshold limit derivation based on cancer endpoints

The available data on renal carcinogenicity (Kurokawa et al. 1983; Kurokawa et al. 1986a; DeAngelo et al. 1998; Wolf et al. 1998) were analyzed with BMDS 3.1, PROAST 66.40 (all but Wolf et al. 1998) and PROAST 69 (Wolf et al. 1998) to derive carcinogenicity potency estimates (Table 10). According to the recommendations in the respective guidances, model averaging was performed (EFSA Scientific Committee 2017; US EPA 2020; RIVM 2021). Both BMDS and PROAST are established approaches to model dose–response curves, details on the differences can be found in e.g. Haber et al. (2018) or WHO (2020). Both approaches were used in the standard and recommended settings mirroring the requirements of the respective guidances. BMDL_10_ values were used as recommended by the modeling results. The details of each single modeling can be found in the supplementary data. The BMDL_10_ values of BMDS and PROAST were generally found lying numerically quite close together with one exception for the female animals. The latter may be due to the underlying data in the tumor incidence dose-reponse curve. The hBMDL_10_ was derived by using an allometric factor of 4 and assuming a daily water consumption of 50 mL/kg rat (ECHA 2012, EFSA 2012). The median hBMDL_10_ (lower 95% confidence limit on the benchmark dose for a 10% human cancer response) derived from the models was 0.65 mg bromate/kg bw/d. This median BMDL_10_ value is identical to the results of the model with the lowest AIC value (i.e. Kurokawa et al. 1986a). One value of 0.02 mg Bromate/kg bw/d was excluded as an obvious outlier compared to the other model results.

### Threshold limit derivation based on non-cancer endpoints

Two subchronic studies (Kurokawa et al. 1990; Dodd et al. 2013) and several chronic studies (Kurokawa et al. 1986a, b; DeAngelo et al. 1998) have demonstrated non-cancer effects in the kidney following oral exposure to bromate. Only the study by DeAngelo et al. (1998) adequately described the dose–response relationship of the non-cancer effects for the derivation of BMDL, NOAEL, or LOAEL values for non-cancer effects. Moreover, this data set proved to be the most sensitive, with a NOAEL of 0.02 g potassium bromate/L (1 mg/kg bw/d).

Assuming a daily water consumption of 50 mL/kg bw for rats (ECHA 2012; EFSA Scientific Committee 2012) and using a default assessment factor of 100, a health-based guidance value to protect from deterministic effects by bromate would be 7.7 µg bromate/kg bw/d, respectively, including the correction from potassium bromate to bromate.

### Comparison of the cancer and non-cancer threshold limit derivation

By linear extrapolation, the health-based guidance value to protect from urothelial hyperplasia by bromate with 7.7 µg bromate/kg bw/d is associated with a renal cancer risk of 0.12% (12/10,000). As this would lead to an unacceptable high tumor risk, the mean hBMDL_10_ of 0.65 mg bromate/kg bw/d for carcinogenicity is taken forward for exposure risk evaluation.

## Exposure assessment

To assess the exposure of swimmers to bromate, three routes of exposure were considered, namely oral, inhalation and dermal. Furthermore, different target groups (infants and toddlers, children and adults), exposure scenarios, including recreational and sport-active swimmers and top athletes, were considered in the calculation of pool water uptake for each exposure route. The default parameters for bodyweight and the specific swimming parameters for the duration per day and the frequency per year were taken from Anses (2012). While Anses (2012) assumed a frequency of 48 swimming sessions per year for children, RIVM (2006) assumed a frequency of 104 times per year. In the present risk assessment, the group of children participating in competitive sports, further referred to as ‘sport-active children’ was addressed separately, assuming a frequency of 238 times per year. For another group of children, it seemed reasonable to assume a long-term average frequency of not more than one pool visit per week. It is acknowledged that these values are somewhat arbitrary and may need to be adjusted to other countries with different climate and habits. Schets et al. (2011) reported lower swimming frequencies than other sources. This is probably due to the fact that the population answering the questionnaire included an unknown number of responders of non-swimmers, thus reducing the calculated average frequency. Their data were therefore not further considered. Footnotes of table S2 describe in detail, why other reported values were not considered for our risk assessment.

For exposure assessment exposure to bromate was averaged over a year. As the risk assessment focuses on a chronic adverse effect of carcinogenicity and an additional life-long extra risk for kidney cancer, this was considered to be justified.

As our risk assessment was not intended to be made in the context of the biocide product regulation, the exposure values used in this paper differ in part from the default values taken by ECHA (2007). Nevertheless, for a better possibility to compare both approaches, an additional exposure assessment for the oral exposure based on the default values and boundary conditions given in the framework of the biocide product regulation was added in the supplementum (table S3).

### Oral exposure route

Ingestion rates were taken from Dufour et al. (2017) and were derived for sport-active groups from Briggle et al. (1981) and Allen et al. (1982). The daily pool water uptake per kg bw and day was calculated for the different target groups based on the data for bodyweight, swimming duration per day and swimming frequency per year given by Anses (2012) (Table 11).

**Table 11:** Exposure scenarios for oral water uptake from pool water

	Ingestion rate^a^ (geometric mean) [mL/h]	Duration per day^b^ [h]	Frequency per year^b^ [d/a]	Body weight^b^ [kg]	Average daily pool water uptake per kg body weight and day [µL/kg bw/d]
Infants and toddlers (0–1 y)	24	0.5	48	10	158
Children (2–15 y)	24	1	48	30	105
Children (sport-active)	127	1.5	238	30	4141
Adults	12	1	48	70	23
Adults (sport-active)	127	2	143	70	1422
Adults (top athletes)	127	5	238	70	5915

^a^For infants, children and adults according to Dufour et al. (2017) and for sport-active groups calculated based on Briggle et al. (1981) and Allen et al. (1982)

^b^According to Anses (2012)

A larger experimental human-biomonitoring study for cyanuric acid determined the ingestion of swimming pool water by 549 children and adult recreational swimmers (Dufour et al. 2017). It is important to note that swimmers were directed to perform normal swimming activities for approximately 1 h for this study and calculation of the ingestion rate was based on the self-reported data of the minutes spent in the water. Thus, this study does not give any information on the average swimming duration in swimming pools per day, and the ingestion rates were suggested by the authors not to be taken too strictly. Values calculated for children were 23.9 mL/h and for adults 12.4 mL/h (geometric means, Dufour et al. 2017).

For sport-active swimmers, an ingestion rate of about 322 mL/h was reported (Dufour et al. 2017), based on a small study with five competitive swimmers (Briggle et al. 1981; Allen et al. 1982). As the uptake for sport-active children was not investigated (Dufour et al. 2017), we used the data given by Briggle et al. (1981) and Allen et al. (1982). These studies differ in the methods: concentration of cyanuric acid in the swimming pool of 29.9 µg/mL (C(p)) and a swimming period of 2 h were applied in one study (Briggle et al. 1981) and a determination of the amount of cyanuric acid excreted via urine within 24 h for five long-distance swimmers between 9 and 17 years was the basis of the other study (Allen et al. 1982). The mean cyanuric acid excretion was 9.8 mg (A(u)). No information on the cyanuric acid concentration in the pool water and the swimming duration were given in this study (Allen et al. 1982). From the available information, the ingested water volume can be calculated as follows:

$$ V(p) = V(u) \times \frac{C(u)}{{C(p)}}{\text{and}}\quad C(u) = \frac{A(u)}{{V(u)}}, $$

*V*(*p*) is the ingested water volume from pool water, *V*(*u*) is the volume of urine (24 h), *C*(*p*) is the concentration of cyanuric acid in pool water, *C*(u) is the concentration of cyanuric acid in urine (24 h) and *A*(*u*) is the amount of cyanuric acid in urine (24 h).

This resulted in an estimated total ingested volume of 328 mL for sport-active swimmers. Considering a 98% recovery rate of cyanuric acid (Allen et al. 1982) the resulting value is 322 mL (Dufour et al. 2017). As this volume refers to the swimming period of 2 h (personal communication, Briggle et al. (1981)), it is divided by two to obtain the ingestion rate per hour, leading to a rate of 161 mL/h (arithmetic mean), the same amount that was calculated previously (Dufour et al. 2006). As ingestion rates were given as geometric means (Dufour et al. 2017), we calculated a geometric mean of 127 mL/h for the sport-active children, based on the same data (single values for each swimmer) (Allen et al. 1982). This average ingestion rate was also used for adults. Nevertheless, it should be considered that, on average, adults might swallow less water than children aged between 9 and 17 years. In conclusion, based on the bodyweight, the range of average daily oral pool water uptake is between 23 µL/kg bw/d for the group of recreational swimming adults and 5915 µL/kg bw/d for adult top athletes.

An overview of other existing values which were checked for their relevance for the further calculation of the average oral water ingestion per day and the reasons for not being further considered for our assessment of the oral exposure are given in Table S2.

To make our data comparable to exposure assessment approaches recommended under the biocide product regulation, in Table S3 water uptake was calculated based on the grouping of swimmers and the corresponding default values for body weight suggested bei ECHA (2007). Indicative exposure values for the ingestion rates based on the same data as above were derived here according to ECHA (2017c): the 75th percentile of the exposure data was used for moderate, the 95th percentile for considerable and the maximum for high data uncertainty. In addition, ConsExpo data for secondary exposure scenario (post-application) was used. Special data for infants and toddlers or sport-active swimmers are not given by ConsExpo. Thus, we used the ConsExpo-data just for the group of the adults. The ConsExpo default value for body weight is 65 kg.

Based on these approaches, the average daily oral pool water uptake would be between 59 µL/kg bw/d for the group of recreational swimming adults and 24,891 µL/kg bw/d for sport-active children of the age 2 to < 6 years and 110 µL/kg bw/d for the group of adults according to ConsExpo as averaged daily exposure. The much higher values are mainly due to the higher exposure values that had to be used due to data uncertainty according to the ECHA (2017c) approach, if the data for the sport-active swimmers of Allen et al. (1982), consisting of only 5 values would be considered with its maximum.

### Inhalation exposure route

Bromate is a non-volatile compound (see also the very low estimated Henry coefficient in the supplement S1.1.). Gas-phase inhalation has been shown to contribute up to 5% of the body burden of swimmers for chlorinated acetates, whereas oral uptake contributed 94% (Cardador and Gallego 2011). As chloro-acetates have a much higher Henry coefficient of about 3.50 × 10^–7^ (m^3^ × Pa)/mol (Bowden et al. 1998) than bromate (about 2.53 × 10^–13^ (m^3^ × Pa)/mol, see S1.2.), it can be assumed that gas-phase inhalation of bromate from pool water is negligible compared to oral uptake.

However, exposure may be possible via aerosol formation. Uptake of disinfection by-products via aerosol formation has been considered of minor importance (ECHA 2017a). In a study on aerosol formation during showering, Zhou et al. (2007) measured an aerosol particle mass concentration of 5000–14,000 µg/m^3^ inside the shower for hot water (43–44 °C, mass median diameter 6.3–7.5 µm) and 20–100 µg/m^3^ for cold water (24–25 °C, mass median diameter 2.5–3.1 µm), the latter being much closer to the temperature generally observed in swimming pools. The recommendation of the FINA (Fédération Internationale de Natation) for the temperature of pool water used by competitive swimmers is 25–28 °C (FINA 2020).

Nevertheless, an aerosol concentration in the given range for hot water of 10,000 µg/m^3^ was used to further calculate the inhalation uptake to be on the safe side.

For hot water, furthermore, an alveolar deposition fraction of about 10% was given by Zhou et al. (2007). Assuming an average aerosol particle mass concentration of 10,000 µg/m^3^ and an alveolar uptake fraction of 10%, an aerosol particle uptake of 1000 µg per m^3^ inhaled air (10%), i.e. 1 µL pool water per m^3^, was derived for further calculations of the inhalation uptake fraction. Exposure scenarios for the inhalation uptake route are summarized in Table 12.

**Table 12:** Exposure scenarios for the inhalation of pool water aerosol

	Aerosol particle concentration^a^ [µL/m^3^]	Inhaled air per hour^b^ [m^3^/h]	Duration per day^b^ [h]	Frequency per year^b^ [d/a]	Body weight^b^ [kg]	Daily pool water inhalation per kg bodyweight and day [µL/(kg bw/d)]
Infants and toddlers (0–1 y)	1	0.05	0.5	48	10	0.0003
Children (2–15 y)	1	1	1	48	30	0.004
Children (sport-active)	1	1.9	1.5	238	30	0.062
Adults	1	1	1	48	70	0.002
Adults (sport-active)	1	3.2	2	143	70	0.036
Adults (top athletes)	1	3.2	5	238	70	0.149

^a^Based on data from Zhou et al. (2007)

^b^According to Anses (2012)

The range of average inhalation daily pool water uptake was between 0.3 nL/kg bw/d for the group of infants and toddlers and 150 nL/kg bw/d for the adult top athletes.

### Dermal exposure route

No studies on the dermal uptake of bromate in humans are available. However, the dermal uptake of bromate is expected to be negligible, as bromate occurs in pool water as anion (SCCS 2018). The fraction taken up via skin while swimming can be approximated in the following way: for the dermal route, an exposure of the total body surface has to be assumed, which is 17,500 cm^2^ for an adult, according to the Scientific Committee on Consumer Safety (SCCS 2018); for children, surface areas according to Phillips et al. (1993) were used. The dermal permeability coefficient Kp of 4.29 × 10^–6^ cm/min was calculated from data of a dermal absorption study for guinea pig skin (Anderson 1994) (S4). The dermal permeability coefficient, Kp, was derived from the absorption rate through guinea pig skin. Due to the higher density of hair follicles, the transfer through guinea pig skin is expected to be considerably higher than through human skin. Therefore, a possible increased absorption through injured or macerated skin suggested by the studies of Gattu and Maibach (2011) has not been further considered by using an additional extrapolation factor. The average daily dermal ‘pool water exposure’ considered for the calculation of dermal bromate uptake, is calculated as follows:

Dermal permeability coefficient [cm/min) × body surface [cm^2^] × (minutes of swimming per day [min] × (days of swimming per year [d] / days of a year [d]) / bodyweight [kg].

Data for all exposure groups are given in Table 13.

**Table 13:** Exposure scenarios for dermal bromate uptake from pool water

	Body surface^a,b^ [cm^2^]	Duration per day^c^ [h]	Frequency per year^c^ [d/a]	Body weight^c^ [kg]	Amount of daily pool water per kg bodyweight and day considered for 100% bromate uptake [µL/kg bw/d]
Infants and toddlers (0–1 y)	6400^a^	0.5	48	10	11
Children (2–15 y)	12,600^a^	1	48	30	14
Children (2–15 y) (sport-active)	12,600^a^	1.5	238	30	106
Adults	17,500^b^	1	48	70	8
Adults (sport-active)	17,500^b^	2	143	70	50
Adults (top athletes)	17,500^b^	5	238	70	210

Permeability coefficient *K*_*p*_ = 4.29 × 10^–6^ cm/min, assumed bromate concentration 1 µg/L

^a^Calculated for children by surface area/body weight ratios given byPhillips et al. (1993)

^b^For adults according to the SCCS (2018)

^c^According to Anses (2012)

### Amount of pool water considered for bromate exposure via all routes of uptake

The total amount of pool water that was considered for the calculation of maximum acceptable bromate concentrations and the corresponding fractions for each uptake pathway are summarized in Table 14. Swallowing swimming pool water represents the main uptake route (73–98%). The dermal uptake of bromate (2–27%) is less relevant and the uptake of bromate via respiration of aerosol can be neglected in this context as it is far below 1%.

**Table 14:** Total amount of pool water to consider for bromate exposure for three uptake routes based on Tables 11, 12, 13

	Water volume per kg bodyweight and day (rounded) [µl/kg bw/d]						
	Oral	Fraction (oral)	Inhalation	Fraction (inhalation)	Dermal	Fraction (dermal)	Sum
Infants and toddlers (0–1 y)	158	0.94	0.0003	0.000002	11	0.06	169
Children (2–15 y)	105	0.88	0.004	0.000037	14	0.12	119
Children (2–15 y) (sport-active)	4141	0.98	0.062	0.000015	106	0.02	4246
Adults	23	0.73	0.002	0.000061	8	0.27	31
Adults (sport-active)	1422	0.97	0.036	0.000024	50	0.03	1472
Adults (top athletes)	5915	0.97	0.149	0.000024	210	0.03	6125

### Derivation of cancer risk-related bromate concentrations in swimming pool water for different exposure scenarios and a risk of 1:100,000

Considering a human cancer potency estimate hBMD_10_ for bromate of 0.65 mg bromate/kg bw/d based on F344 rat renal tumors (see previous chapter, Table 10) and the relevant pool water volume summarized for each uptake pathway (oral, dermal, inhalation), different target groups (infants, children, adults) and exposure scenarios (recreational, sport-active, top athletes) (Table 14) the corresponding bromate concentration in swimming pool water can be calculated for the cancer risk of interest. If, for example, a tolerable maximum additional theoretical lifelong cancer risk of 1:100,000 is addressed, which corresponds to the risk taken as a basis for the derivation of bromate drinking water guidance values by several institutions (World Health Organization 2017; Health Canada 2018; US Environmental Protection Agency, Office of Water 2018; EU 2020), the calculated maximum bromate concentrations that result for pool water would be in the range between 0.011 and 2.096 mg/L bromate (Table 15). It is a wide range mostly because the amount of swallowed water differs widely between the target groups (Table 14).

**Table 15:** Derivation of cancer risk-related bromate concentrations in swimming pool water considering uptake via all routes (oral, inhalation, dermal) (see Table 14) and a value of 65 ng/kg bw/d for an additional theoretical life-long cancer risk of 1:100,000 derived from the hBMDL_10_

	Volume per kg body weight and day^a^ [µL/kg bw /d]	Additional theoretical lifelong cancer risk of 1:100,000^b^ [ng Bromate / kg bw /d]	Bromate concentration in swimming water for a risk of 1:100,000 [mg/L]
Infants and toddlers (0–1 y)	169	65	0.385
Children (2–15 y)	119	65	0.544
Children (2–15 y) (sport-active)	4246	65	0.015
Adults	31	65	2.096
Adults (sport-active)	1472	65	0.044
Adults (top athletes)	6125	65	0.011

^a^As calculated in Table 14

^b^Based on the hBMDL_10_ of 0.65 mg bromate/kg bw/d as calculated in Table 10. The hBMDL_10_ represents an additional theoretical life-long cancer risk of 1:10 (10%), lower bound estimate of the confidence interval

The data show that among the three target groups of infants (0–1 year), children (2–15 years) and adults referring to the same cancer risk, lower maximum bromate concentrations would be acceptable for children (0.544 mg/L) and infants (0.385 mg/L) than for adults (2.096 mg/L). Between one and two orders of magnitude lower maximum bromate concentrations are derived for sport-active children (0.015 mg/L) as well as for adults (0.044 mg/L). The lowest maximum bromate concentration was calculated for the group of adult top athletes (0.011 mg/L) (Table 15).

In the supplementum (Table S4), cancer risk-related bromate concentrations calculated according to the approaches for exposure assessment (ECHA 2007, 2017c) used under the biocide product regulation for an additional theoretical life-long cancer risk of 1:100,000 and 1,000,000 are given. For a risk of 1:1,000,000 maximum bromate concentrations would be in the range of 0.0004 mg/L for sport-active children (age 6 to < 12 years) and adults (top athletes) and 0.11 mg/L for adults (recreational swimmers).

## Discussion

### Formation and occurrence of bromate in swimming pool water

National and international standards require the disinfection of pool water to protect swimmers against microbial infections. Due to the disinfection processes, bromate may be produced by accident. As bromide ions may be present in natural freshwater and especially in seawater, any treatment of these waters by chlorine, ozone or hypochlorite is liable to oxidize bromide to bromate. Background levels of bromate in sea water are below 1 µg/L (Chen et al. 2006; Zakaria et al. 2011; Lim and Shin 2012).

Bromate was rarely detected in samples from fresh water pools (Table 2). This is due to the low concentrations of bromide. The high concentration of bromide in sea water may result in the formation of bromate at high levels. However, this is not necessarily the case, since there are several sea water swimming pools with chlorine as the disinfecting agent, where bromate was not detectable, i.e. below 200 µg/L. Considering the relatively high LLOQ, there is no evidence that these samples would comply with a lower threshold value. Other pools exhibit high or even very high concentrations of bromate. In some cases, the source of bromate formation was identified in the treatment process. In these facilities the disinfectant, chlorine, is produced in situ by electrolysis of seawater. Due to the high concentration of bromide in seawater it is impossible to prevent the formation of bromate by electrolysis. This formation would not occur if pure sodium chloride brine would be used as the electrolyte instead of seawater. However, the reason for the high bromate formation rate could not be identified in one seawater pool; one scenario has been monitored where after a complete water exchange concentrations increased from 2.5 to 14.6 mg/L within two months (Table 4).

Several physical and chemical properties influence the final content of bromate in pool water (supplement S3), and it was claimed that by controlling of some of these parameters, the bromate concentration can be kept at or below 100 µg/L when using bromide/ozone treatment (Hoffmann 2015).

Concerning swimming pool water limit values, it has to be considered that especially for seawater the LOQ of 0.1–0.2 mg/L compared to the LOQ of fresh water of ~ 0.02 mg/L is significantly higher than the calculated maximum acceptable bromate concentrations (risk of 1:100,000) for the population group of sport-active swimmers (0.011—0.044 mg/L).

### Critical endpoint/critical study

Bromate was shown to induce cancer in F344 rats by two independent research groups (Kurokawa et al. 1990; DeAngelo et al. 1998). There was no clear evidence for carcinogenicity in mice or hamsters (Table 8). In rats, the tumors induced were renal and thyroid follicular adenomas and adenocarcinomas, and mesotheliomas of the tunica vaginalis. The follicular cell tumors of the thyroid and the mesotheliomas were not considered adequate for human cancer risk assessment due to an assumed higher sensitivity of rats compared to humans and for other reasons described above.

### Mode of action

Bromate-induced protein droplet accumulation containing α2u-globulin in renal tubule cells of male rats. In general, the α2-macroglobulin induction MOA is considered a reason for renal cell tumor development specifically in male rats. However, bromate caused renal cell tumors in both male and female rats with a similar potency (Kurokawa et al. 1983). Thus, α2-macroglobulin does not seem to contribute significantly to renal cell tumor induction in rats, leaving genotoxicity as the primary MOA in this case. However, alternative MOAs might contribute to bromate-induced carcinogenicity of other tumors. These MOAs include thyroid and sex hormone imbalance, alterations of apoptosis and immunosuppression. Bromate and its metabolites were reported to cause indirect and direct damage to the thyroid gland. Indirect damage includes NIS inhibition followed by TSH stimulation. Direct damage of bromate in thyroids includes oxidative damage followed by DNA-strand breaks. Consequently, there is a relevant uptake of bromate into the thyroid gland leading to cellular damage and disruption of thyroid hormone homeostasis. The induction of DNA strand breaks in combination with compensatory cell proliferation to balance thyroid hormone homeostasis is regarded as a potential MOA for tumors of the thyroid gland. In a similar way the altered balance of sex hormones can potentially result in promotion of tumors. Potassium bromate-treated rats showed clearly reduced levels of FSH, LH and testosterone, accompanied by reduced levels of antioxidant enzymes. These alterations were potential contributors to induction of observed mesotheliomas in the tunica vaginalis of the testis. Further, sodium bromate has been suggested to suppress apoptosis by downregulation of TRAF3, NF-kB and IL1 and to influence macrophage reactivity against tumor cells. Consequently, apoptosis suppression may allow for survival and replication of cells with DNA damage, while additional suppression of the immunological tumor defense allows the survival and progression of mutated cells increasing the likelihood of tumor development.

### Cancer potency estimates

The renal cell tumors in rats were used as point of departure for human cancer risk assessment in the present study. The median hBMDL_10_ value derived was 0.65 mg Bromate/kg bw/d (range 0.16–1.02). BMDS 3.1 and PROAST versions 66.40, 67 and 69 generally provided similar results (Table 10) except for the renal cell tumor in female rats. This was due to the underlying dose–response data. Our modeling results were similar to the hLEDL_10_ derived by US EPA (2001) and OEHHA (2009), with 0.59 mg bromate/kg bw/d and 0.96 mg bromate/kg bw/d, respectively, and also with the hBMDL_10_ value of 1 mg bromate/kg bw/d derived by Health Canada (2018), respectively (Table 16).

**Table 16:** Existing cancer potency estimates for bromate based on male F344 rat renal tumors

Cancer potency [mg bromate/kg bw/d]	Type	Species, sex	Study	Extrapolation method	Source
0.59	hLEDL_10_^a^	Male F344 rat	DeAngelo et al. (1998)	Multistage Weibull time-to-tumor model, scaling	US EPA (2001)
0.96	hLEDL_10_			Multistage Weibull time-to-tumor model, scaling	OEHHA (2009)
1.00	hBMDL_10_^b^			Multistage Weibull time-to-tumor model, PBPK, scaling	Health Canada (2018)

^a^Lower-bound estimate of the average lifetime dose associated with a ten percent human cancer risk, lower 95th percentile

^b^Lower 90% confidence limit of the benchmark doses for a 10% human cancer response

### The derived bromate concentrations in swimming pool water compared to existing regulatory values

Values for bromate in pool water and drinking water proposed by various organizations are given in Table 6. In all cases, as with our approach, carcinogenicity was the key endpoint of concern, and linear extrapolation using a non-threshold approach has mostly been used for the derivation of pool water and drinking water values (supplemental information S5). This implies that bromate is considered to be a genotoxic carcinogen and a substance without effect threshold. By contrast, the German national standard for bromate in pool water (UBA 2014) was derived considering bromate as a carcinogen with an effect threshold resulting in a limit of 2 mg bromate/L that was also set within the DIN 19,643–1:2012–11. Without exception in previous risk assessments as well as in ours, oral uptake was considered the relevant route of exposure to bromate via pool water, while the dermal and inhalation exposure are expected to be negligible when deriving guidance values for bromate in swimming pool water. Our estimated dermal exposure using a permeability coefficient Kp of 4.29E-06 cm/min on the basis of Anderson (1994) (cf. chapter 7.3), with a maximum dermal fraction of 27% of all exposure pathways in case of adults, represents a conservative assumption, since a significantly lower bromate absorption should be assumed for human skin compared to guinea pig skin. Similarly, our estimate of the inhalation exposure to bromate using an average aerosol particle mass concentration of 10,000 µg/m^3^ for a water temperature of 43–44 °C also represents a conservative value (cf. chapter 7.2).

Naturally, both the dose response assessment of bromate as a genotoxic chemical substance and the exposure assessment have a decisive impact on the result of risk characterization.

Whether an additional carcinogenic lifetime risk of 1:100,000 should be taken as a basis when deriving bromate pool water values, as in most of the existing proposals, is ultimately also a question of risk management in the specific individual case. Although there is no EU legislation setting the 'tolerable' risk level for carcinogens in the society, cancer risk levels of 1:100,000 and 1:1,000,000 could be seen as indicative tolerable risks levels for workers and the general population, respectively (ECHA 2012).

A detailed comparison of the suggested values according to Table 6 and our derived values is challenging partly because the derivation of the specific bromate pool water guidance values is incompletely described. The oral doses based on renal tumors, all attributed to a lifetime cancer risk of 1:100,000, ranging from 0.0143 (Anses 2012) to 0.065 µg/kg bw/d (this study) and are within one order of magnitude. Anses' bromate pool water values and the values we derived (Table 15) are comparable; however, for infants and toddlers, as well as adults (recreational and sport-active swimmers), larger differences with factors of 6.4 (infants/toddlers), 5.6 (adult occasional swimmers), and 4.4 (adult sport-active swimmers), were observed. This can mainly be explained by discrepancies in the assumed ingestion rates (Table 11, Table S2) and the oral body doses (Table 6) but to a lesser extent by differences of the exposure scenarios for the inhalation of pool water aerosol or dermal bromate uptake. Since Anses used the (higher) arithmetic means instead of the geometric means to describe the ingestion rates, these differ from our data by approximately a factor of 2.3 (Dufour et al. 2017). RIVM (2014) has derived bromate pool water guidance values for toddlers, adults, and competitive swimmers, however, without any further differentiation of the individual exposure groups. RIVM (2014) finally set a guidance value of 0.1 mg bromate/L for an extra cancer risk of about 1:100,000 (Table 6) using an oral body dose of 0.05 µg/kg bw/d. The values of RIVM are comparable to those bromate concentrations derived by us, with the exception of the values for adults and sport-active swimmers. Lower values (0.117 mg/L vs. 2.1 mg/L) were derived by RIVM because RIVM based its calculation for adults on parameters for the duration and frequency of swimming taken from data for competitive swimmers as ‘worst case’ for adults in general (Table S2). Additionally, an oral ingestion rate of 50 mL per hour is suggested by RIVM (2016) also used as default value in ConsExpo for the secondary exposure scenario (post-application), which is more than four times higher than the 12 mL per hours derived by Dufour et al. (2017). As it is not completely clear, how RIVM derived the 50 mL per hour and the data of Dufour et al. (2017) is based on human biomonitoring and *n* = 362 swimmers, it seems reasonable to harmonize the RIVM/ConsExpo default data used under the biocide product regulation with the results of Dufour et al. (2017). Even if indicative exposure values according to ECHA (2007) are derived, the 75th percentile for moderate data uncertainty results still in a lower ingestion rate of 27 mL per hour.

For disinfection by-products like bromate, for which inhalation and dermal exposures are of minor importance, existing drinking water guidance values or standards for bromate are considered to be adequately protective for swimming pool water. The reason is that the ingested amount of water during swimming is lower than 2 L per day over a lifetime, which is the default assumption for the derivation of drinking water limits. Thus, ECHA (2017c) suggested using drinking water limits as a first-tier approach to assess bromate in pool water. Using drinking water limits of bromate as suggested by ECHA to assess chemical substances in pool water would overestimate the health risk. Risk-related bromate drinking water standards associated with the excess cancer risk of 1:100,000 account for 2 µg/L (World Health Organization 2017) or 4 µg/L (Health Canada 2019) (Table 6). A somewhat higher provisional bromate drinking water guideline value and limit value, respectively, have been set by World Health Organization (2017) and EU (2020) with a bromate concentration of 10 µg/L. The World Health Organization (2017) pointed out that their guideline value is provisionally higher because of limitations in available analytical and treatment methods. These health-based bromate drinking water values are lower than recommended maximum bromate concentrations in pool water derived even for the most susceptible exposure group of competitive swimmers, with values of 8.7 µg/L (Anses 2012), 11 µg/L (this study), and 58.5 µg/L (RIVM 2014). If calculations would be based on the indicative exposure values according to ECHA (2007) in biocide product regulation combined with a protective risk level for the general population of 1:1,000,000 even lower tolerable bromate concentrations would result down to 0.3–0.4 µg/L for sport-active children and adult top athletes (Table S4).

### Further aspects

Pool water risk assessment covering all disinfection by-products (DIBP) is beyond the scope of this study. A first cursory look revealed that the quantitative differences between bromine-based disinfection versus chlorine-based disinfection almost all concern levels of the bromoform and chloroform. For mixed trihalomethanes, chlorinated pool water shows higher levels than brominated pool water, and the sum of irritating chloro-amines in water and air achieved slightly lower concentrations in case of brominated pool water (Richardson et al. 2010; Hoffmann 2015), perhaps because in the reduction of free chlorine, bromide competes with mono- and dichloramine (Luong et al. 1982). A guidance document addressing DBP was published by the European Chemicals Agency (ECHA 2017a).

It also has to be considered that no extra factor was applied for children, although this is proposed for mutagenic substances, e.g., by Risk Assessment Forum (2005). Furthermore, the derived bromate levels in pool water do not consider possible other sources. Only the allocation to bathing water was considered. There might be other sources such as the drinking water or the use of potassium bromate as an additive in flour (E924) (Johnson 2013), which, although banned in the EU since 1990, is still permitted in other countries, e.g., the USA (Shanmugavel et al. 2020).

## Conclusion

There is convincing evidence from a multitude of studies that bromate induces oxidative DNA damage and acts as a clastogen in vitro and in vivo. Since statistical modeling of the available genotoxicity data is compatible with both linear and non-linear dose–response relationships, bromate should be considered as a non-threshold carcinogen. A threshold-like MOA for the genotoxicity of bromate may be possible; however, it can currently not be assumed with reasonable certainty. In addition, the data do not allow the calculation of a quantitative estimate of a potential existing threshold dose. BMD modeling with model averaging for renal cancer studies (Kurokawa et al. 1983; Kurokawa et al. 1986a; DeAngelo et al. 1998) resulted in a median hBMDL_10_ of 0.65 mg bromate/kg bw /day.

Evaluation in different age and activity groups revealed that top athletes had the highest exposure, followed by sport-active children, sport-active adults, infants and toddlers, children and adults.

The predominant route of exposure was oral (73–98%), by swallowing water, followed by the dermal route (2–27%); whereas the inhalation route was insignificant (< 0.5%).

Accepting the same risk level for all population group results in different guidance values due to the large variation in exposure. For an additional risk of 1:100,000, the bromate concentrations would range between 0.011 for top athletes, 0.015 for sport-active children and 2.1 mg/L for adults and 0.385 to 0.544 mg/L for non-sport active children including toddlers and infants.

## Supplementary Information

Below is the link to the electronic supplementary material.

Supplementary file1 (DOCX 391 KB)

Supplementary file2 (XLSX 37 KB)

Supplementary file3 (XLSX 37 KB)

Supplementary file4 (XLSX 37 KB)

Supplementary file5 (XLSX 37 KB)

Supplementary file6 (XLSX 37 KB)

Supplementary file7 (XLSX 37 KB)

Supplementary file8 (XLSX 37 KB)

Supplementary file9 (XLSX 38 KB)

Supplementary file10 (XLSX 37 KB)

Supplementary file11 (XLSX 11 KB)

Supplementary file12 (TXT 0 KB)

Supplementary file13 (XLSX 272 KB)

Supplementary file14 (DOCX 159 KB)

Supplementary file15 (TXT 0 KB)

Supplementary file16 (TXT 0 KB)

Supplementary file17 (DOCX 110 KB)

Supplementary file18 (DOCX 141 KB)

Supplementary file19 (XLSX 312 KB)

Supplementary file20 (XLSX 313 KB)

Supplementary file21 (TXT 0 KB)

Supplementary file22 (XLSX 272 KB)

Supplementary file23 (DOCX 155 KB)

Supplementary file24 (TXT 0 KB)

Supplementary file25 (XLSX 354 KB)

Supplementary file26 (DOCX 121 KB)

Supplementary file27 (DOCX 118 KB)

Supplementary file28 (TXT 0 KB)

Supplementary file29 (XLSX 313 KB)

Supplementary file30 (DOCX 107 KB)

Supplementary file31 (TXT 0 KB)

Supplementary file32 (XLSX 272 KB)

Supplementary file33 (DOCX 56 KB)

Supplementary file34 (TXT 0 KB)

Supplementary file35 (XLSX 353 KB)

Supplementary file36 (DOCX 108 KB)

Supplementary file37 (TXT 0 KB)

Supplementary file38 (XLSX 312 KB)

## Footnotes

1 The equations shown are a simplification; in aqueous solutions, the ions Br^−^, Br_3_^−^, OBr^−^ and BrO_3_^−^ result from/in dis- and com-proportionation reactions of Br_2_. At low pH, compropotionation is favored, whereas at higher (alkaline) pH values the disproportionation predominates. The reaction may oscillate around the equilibrium, and the bromate-bromide system is a model for oscillating reactions (p.e. Zaikin and Zhabotinsky 1970; Toporek et al. 2015).
