# Supplementary material for: New aspects in deriving health-based guidance values for bromate in swimming pool water
Source: Arch Toxicol. 2022 Apr 6;96(6):1623–59. doi: 10.1007/s00204-022-03255-9 (PMC9095538; doi:10.1007/s00204-022-03255-9)
Supplement: Supplementary file 1 — Supplementary file1 (DOCX 391 KB) [file 204_2022_3255_MOESM1_ESM.docx]

## **Supplementum**

## **S 1 Physical data of sodium- and potassium bromate, Henry coefficient, analytical methods**

## **S 1.1. Henry coefficient**

The Henry coefficient has not been determined for neither potassium nor sodium bromate, and European Union (EU) 2006 does not require to measure / calculate a Henry coefficient for substances with melting points above 300°C. The Henry coefficient would allow for a rough estimate for the concentration of a compound as gas in the atmosphere (or its vapour pressure) in equilibrium with its concentration in water. It is generally accepted that salts have a negligible Henry coefficient. This is because the equilibrium has to be established between non-dissociated salt in water and in air. Therefore, the expectation is that inhalation of bromate in gaseous state (as potassium bromate or as sodium bromate) is of no relevance. The evidence for this expectation is as follows: For the approximative calculation of the Henry coefficient, the vapor pressure of a substance can be divided by its water solubility. The higher the vapor pressure and the lower the water solubility, the higher the Henry coefficient and the higher the equilibrium concentration in the gas phase. For a worst-case assumption (i. e. high gas concentration), the lower of the two water solubilities given in the table above shall be used, which is 66 g/L = 395 mol/m³ for potassium bromate. However, data for the vapor pressure of potassium or sodium bromate are not available. As a substitute, the vapor pressure for potassium bromide (KBr) is used. At 900 K and 637 K, the vapor pressure of KBr is 3.348 and 3.43 × 10^-5^ Pa, respectively (Zimm and Mayer, 1944). The vapor pressure extrapolated to 293 K is 1.16 × 10^-23^ Pa. With these data, the estimated Henry Coefficient for potassium bromate is

$${H'}_{293 K}=\frac{{1.16\times10}^{-23}Pa\times m^{3}}{395 mol}=\frac{{2.94\times10}^{-26} Pa\times m^{3}}{mol}\to H=1.21\times{10}^{-29}$$

with R = 8.314 J/(mol × K) as gas constant and T as temperature in K (293 K). If a pool water would contain 1 g/L sodium bromate and the dissociation is assumed to be 99 %, 10 mg/L would be undissolved, which is 0.067 mol/m³. The calculated equilibrium concentration in air for the undissolved bromate was 0.067 mol/m³ x 1.21 x 10^-29^ = 8.07× 10^-31^ mol/m³ = 1.35 × 10^-22^ µg/m³. That is, you need to breath 2 Mio m³ air to inhale 1 molecule potassium bromate, if it was available in the gas state, only. Although this calculation uses the vapor pressure of potassium bromide as substitute for potassium bromate, the assumption that gas phase potassium bromate does not contribute to inhalation uptake; any inhalation exposure to potassium bromate in the area of swimming pools is attributable to dissolved material in water spray.

## **S 1.2. Physical data**

Physical data of the bromate salts are listed in the REACh registration dossiers (ECHA-Sodium-bromate 2019; ECHA-Potassium-bromate 2019) and listed in the following table:

| **Compound** | **CAS-No.** | **Molecular mass** [g/mol] | **Melting Point** [°C] | **Water solubility** [g/L] |
| --- | --- | --- | --- | --- |
| NaBrO_3_ | 7789-38-0 | 150.89 | 391 | 364 at 20 °C |
| KBrO_3_ | 7758-01-2 | 167.01 | 350 | 66 at 20 °C |

## **S 1.3. Analytical methods**

A variety of different analytical methods have been used to determine bromate in the water matrix (Michalski und Mathews 2007). The bromate ion can be separated from other ions by use of ion chromatography or by electrophoresis. The separation is to be followed by a detection method. After column separation, the usual detection methods are conductivity detection (CD), post-column reaction (PCR) or mass spectrometry (MS) (Butler, Lytton et al. 2005).

Reversed-phase HPLC (C-12 column) with triple-quadrupole mass spectrometry was used for the simultaneous quantification of perchlorate, bromate, iodate and chlorate at detection levels of 21, 45, 70 and 45 ng/L, respectively (Snyder, Vanderford et al. 2005). Mass spectrometric analysis allows an unanimous identification of bromate.

The international standard DIN EN ISO 11206:2013-05 uses ion chromatography for bromate separation and post-column reaction of iodide with bromate to I_3_^-^, which is quantified photometrically with a detection limit of 0.5 µg/L. The standard DIN EN ISO 15061:2001-12 uses ion chromatography for separation and either conductivity or photometric detection for the quantification of bromate with a detection limit of 0.5 µg/L. Post-sampling treatment of the water samples are described in the standards to limit false-positive and false-negative results.

A very sensitive method for bormate detection was published by Lin and Chin (2012). Bromate is transformed into a derivative by reaction with 2,6-dimethylphenol, solid phase extraction and GS-MS analysis. The limit of detection (LOD) is 0.016 µg/L, the limit of quantification (LOQ) is 0.051 µg/L.

## **S 2 Kinetics of bromate formation**

The kinetics of bromate formation was investigated in 1983 (Haag and Holgne 1983). Reaction pathways and second order reaction rate constants (k_i_) are

$O_{3}+ {Br}^{-}\to O_{2}+{OBr}^{-};$ k_1_ = 160 L/(mol x s) (**1**),

$O_{3}+{OBr}^{-}\to2O_{2}+{Br}^{-};$ k_2_ = 330 L/(mol x s) (**2**),

$2O_{3}+{OBr}^{-}\to2O_{2}+{BrO}_{3}^{-};$ k_3_ = 100 L/(mol x s) (**3**),

and the kinetic equations are

$\frac{d\left[ HOBr \right]_{total}}{dt}=k_{1}\times\left[ {Br}^{-} \right]\times\left[ O_{3} \right]-\alpha\times\left( k_{2}+k_{3} \right)\times\left[ HOBr \right]_{total}\times[O_{3}];$ (**4**),

$\frac{d\left[ {BrO}_{3}^{-} \right]}{dt}=\alpha\times k_{3}\times{[HOBr]}_{total}\times[O_{3}]$; (**5**),

$\frac{d\left[ O_{3} \right]}{dt}=-(k_{1}\times\left[ {Br}^{-} \right]\times\left[ O_{3} \right]+\alpha\times\left( k_{2}+k_{3} \right)\times\left[ HOBr \right]_{total}\times[O_{3}]$; (**6**).

[HOBr]_total_ = [HOBr] + [OBr^-^]; α is the degree dissociation of HOBr at a given pH value (α = [OBr^-^]/[HOBr]_total_). HOBr has an acid value of pK_a_ = 8.76 (Haag and Holgne 1983). The reaction 3 HOBr -> 2 Br^-^ + BrO_3_^-^ + 3 H^+^ is negligible slow at pH ≤ 8 and becomes negligible below pH = 6. (Siddiqui und Amy 1993)

As the hypohalogenite acid, and not the free anion has the antimicrobial activity, it is desirable to keep the water at neutral or slightly acidic pH values. Against this requirement, bromine is more favorable than chlorine as HOBr has a pKa of about 8.7, whereas the pKa of HOCl is 7.5 (Brugger 2014).

**S 3 Factors influencing bromate formation during pool water disinfection with ozone**

The yield of bromate generated during ozonation of water is dependent on several factors, and an empirical relation was published that shows excellent matches with measured data (Song et al. 1996):

[BrO_3_^-^] = 10^-6.11^ x [Br^-^]^0.88^ x [DOC]^-1.18^ x [NH_3_-N]^-0.18^ x [O_3_]^1.42^ x pH^5.11^ x [IC]^0.18^ x t^0.27^, (**1**)

where bromate and bromide are measured in µg/L, dissolved organic carbon (DOC), ammonia-nitrogen (as precursors for bromo-amines), ozone and inorganic carbon (IC as CaCO_3_) are measured in mg/L, and t is the contact time in minutes. This relation shows the strong influence of the pH value and the negative influence of ammonia like bromo-amine precursors (NH_3_-N) and DOC as they tend to decrease OBr^-^ and ozone. The model was validated for a maximum bromate concentration of 300 µg/L. Equation **1** is valid for 20 °C; at higher temperatures, the yield of bromate increases:

[BrO_3_^-^]_T_ = [BrO_3_^-^]_T=20_ x (1.043)^(T – 20)^ (**2**).

**S 4 Calculation of a permeability coefficient for the dermal bromate uptake**

In an in-vitro experiment with guinea pig skin, an area of 1.77 cm² was exposed to 16.5 mg bromate dissolved in 0.2 mL (~0.2 g, concentration 82.5 mg/cm³ = 0.645 mmol/cm³). After an exposure time of 15 and 30 minutes, respectively, Anderson (1994) reported data for bromide in 5 mL receiving fluid (**table S 4.1**).

**Tab. S 4.1**: Average levels of bromide analysed in 5 mL receiving fluid after in-vitro exposure to 0.645 mmol/cm³ bromate on 1,77 cm² guinea pig skin for different exposure- and collection times reported by Anderson (1994) converted in mmol.

| **Collection time** | **15 min exposure** | | **30 min exposure** | |
| --- | --- | --- | --- | --- |
| [min] | [ppm] | [mmol] | [ppm] | [mmol] |
| 30 | 0.7 | 4.38E-05 | 0.85 | 5.31E-05 |
| 60 | 1.35 | 8.44E-05 | 0.9 | 5.63E-05 |
| 120 | 2.15 | 1.34E-04 | 2.1 | 1.31E-04 |
| 240 | 1.65 | 1.03E-04 | 2.35 | 1.47E-04 |

For material transport over a boundary layer, Fick’s first law of diffusion can be applied

$\frac{dn}{dt}=-D\times A\times\frac{dc}{dx},$

with dn/dt the number (or mass) of molecules (n) transported per time (t), D being the diffusion constant, A being the contact surface and dc/dx being the concentration gradient (**figure S 4.2**).

b

C

x

**Fig. S 4.2** Illustration of the transport process via a barrier (yellow)

Under condition that the concentration gradient along the transport pathway x does not change, dc can be replaced by ∆c; this is justified if over the period of the experiment the concentrations on both sides of the barrier change only marginally. The barrier b is constant (dx = ∆x). Under these conditions, dn/dt can be replaced by ∆n/∆t. With a new transfer constant K_p_ = - D/b (permeability coefficient), the equation simplifies to

$\frac{\Delta n}{A\times\Delta t}=\Delta c\times K_{p}$, and with data given in **table S 4.1**, corresponding K_p_ values can be calculated (**table S 4.3**).

**Tab. S 4.3**: K_p_ values (permeability coefficients) in dependence on exposure and collection time based on experimental data reported by Anderson (1994).

|  | **K_p_** [cm/min] | |
| --- | --- | --- |
| **Collection time** [min] | **15 min exposure** | **30 min exposure** |
| 30 | 2.56E-06 | 1.55E-06 |
| 60 | 4.94E-06 | 1.64E-06 |
| 120 | 7.82E-06 | 3.83E-06 |
| 240 | 6.01E-06 | 4.29E-06 |

The K_p_ values are in a range from 1.55E-06 to 7.82E-06 cm/min. For the estimation of dermal absorption of bromate from pool water the longer exposure time of 30 min was taken for further calculation as being closer to general duration times of swimming. Anderson (1994) has reported that after cessation of exposure, the resorption of bromate on the skin progresses. Therefore, the K_p_ value of 4,29E-06 cm/min calculated for a 30 min exposure and 240 min collection time was taken to approximate the dermal uptake of bromate for swimmers.

It has to be born in mind that in the study of Anderson (1994) bromide and not bromate was analysed in the receiving fluid, thus, it remains some uncertainty to which extent bromate itself was penetrating the skin.

Although it is acknowledged that the data presented in the publication of Anderson (1994) do not necessarily represent the dermal penetration of bromate, similar results were generated with other ions. Tregear (1966) reported dermal resorption of radioactive bromide. For NaBr, a 0.15 M solution was applied (1,5E-04 mol/cm³) and a dermal resorption of 8.2E-09 mol/(cm² x min) was observed in rabbits in vivo, which results in a Kp = 5,47E-05 cm/min. On the ventral forearms of male volunteers, the penetration of labelled bromide was 1.1E-10 mol/(cm² x min) which means a Kp of 7,3E-07 cm/min. For radiolabelled phosphate on rabbit skin in vivo, the resorption was 3.4E-09 mol/(cm² × min) which is results in a Kp of 1.1E-04 cm/min.

Paweloszek et al. (2016) found for bromide on porcine skin in vitro a Kp of 6.4E-09 m/s = 3,8E-05 cm/min.

**S 5 Regulatory toxicological guidance values for bromate**

**S 5.1 Oral exposure**

Various international organizations have assessed the risk associated with the exposure to bromate in pool or drinking water. Carcinogenicity has been assessed as the key endpoint of concern, and linear extrapolation using a non-threshold approach has been used in most cases.

The International Toxicity Estimates for Risk (ITER) database enlisted for bromate (CASRN 15541-45-4) only data from US EPA 2021 on the oral (and inhalation) exposure pathways (non-cancer/cancer). U.S. EPA derived a Reference Dose (RfD) of 0.004 mg Bromate-ion/kg-day. The critical end point is kidney effects in rats, to which potassium bromate was administered with drinking water for more than 2 years (DeAngelo et al. 1998). The Point of Departure was the NOAEL of 1.1 mg bromate-ion/kg-day. In addition, an Uncertainty Factor of 300 was used.

US EPA (2001) has also evaluated the carcinogenicity of bromate under its IRIS program. According to its "Proposed Guidelines for Carcinogen Risk Assessment" (U.S. EPA, 1996), US EPA (2001) determined that bromate should be evaluated as a likely human carcinogen by the oral route of exposure. Based on the 2-year drinking water study by DeAngelo et al. (1998), U.S. EPA estimated an Oral Slope Factor of 0.7/(mg / kg-day). Cancer target organs were kidney, testis, and thyroid tumours. The extrapolation method used was the one-stage Weibull time-to-tumor model. This Oral Slope Factor is equivalent to a Risk Specific Dose (RSD) of 1.4 x 10^-5^ mg / kg-d (= 1 x 10-5/ 0.7/(mg / kg)-day) for an additional lifetime cancer risk of 1:100000. The Drinking Water Unit Risk can then be calculated as 2 x 10^-5^ per μg/L. The corresponding risk-related drinking water concentrations are then as follows:

**Risk Level** **Concentration**

10^-4^ (1 in 10,000) 5 µg/L

10^-5^ (1 in 100,000) 0.5 µg/L

10^-6^ (1 in 1,000,000) 0.05 µg/L

Drinking Water Standards and Health Advisories (DWSHA) are issued periodically by U.S. EPA´s Office of Water. Within its Health Advisory (HA) Program, U.S. EPA publishes concentrations of drinking water contaminants at Drinking Water Specific Risk Level Concentration for cancer (10-4 Cancer Risk) and concentrations of drinking water contaminants at which noncancer adverse health effects are not anticipated to occur over specific exposure durations. The one-day HAs, ten-day HAs, and lifetime HAs are not to be construed as legally enforceable federal standards. In contrast, an enforceable Maximum Contaminant Level (MCL) represents the highest level of a contaminant that is allowed in drinking-water. MCLs are set as close as feasible to the Maximum Contaminant Level Goal (MCLG) using the best available analytical and treatment technologies and taking cost into consideration. U.S. EPA (2018) recommended the following bromate values (mg/L): MCLG zero, MCL 0.01, DWEL 0.14, and 0.005 mg/L at 10^-4^ Cancer Risk.

Office of Environmental Health Hazard Assessment (OEHHA) (2009) derivation of PHGs (Public Health Goal) for Bromate in Drinking Water used also the data from DeAngelo et al. (1998). Some differences between the OEHHA (2009) and US EPA (2001) analyses that could account for the resulting human cancer potencies (**Tab. 16**) are the diverse models used, differences in counts of the site-specific tumor incidence, and tumor coding.

World Health Organization (2017) has set a Provisional Guideline Value of 0.01 mg/L (10 μg/L). The basis of the guideline value derivation is an upper-bound estimate of cancer potency for bromate of 0.19 per mg/kg body weight per day, based on low-dose linear extrapolation (a one-stage Weibull time-to-tumour model was applied to the incidence of mesotheliomas, renal tubule tumours and thyroid follicular tumours in male rats given potassium bromate in drinking-water, using the 12-, 26-, 52- and 77-week interim kill data). A health-based value of 2 μg/L is associated with the upper-bound excess cancer risk of 1:100,000. WHO´s guideline value is provisional because of limitations in available analytical and treatment methods.

The key assumptions in the WHO and U.S. EPA evaluations are that bromate causes testicular, kidney and thyroid cancer in humans based on the experimental rodent studies, the cancer response is linear in the low-dose range, and the combined (all sites) cancer risk scales directly from animals to humans on a per-body-weight basis (WHO) or with body weight to the 3/4 power (EPA) (Fawell und Walker 2006). In the most recent Guidelines for Health Canada (2019) the maximum acceptable concentration (MAC) of 10 µg/L from the last update in 1998 was reaffirmed (Health Canada 2019). Again, DeAngelo et al. (1998) was chosen as the key study to assess the carcinogenic risk associated with the ingestion of bromate in drinking water. The health basis of the MAC are tumours of the testicular mesothelium (classified as a possible human carcinogen). The mesotheliomas of the tunica vaginalis provide the most conservative estimate of cancer risk and are therefore used to estimate the health-based values (HBV) for the carcinogenic risk assessment using the non-threshold approach. Thus, the HBV for bromate in drinking water associated with an excess lifetime cancer risk of 1:100,000 is determined to be 0.004 mg/L (4 μg/L). HBVs of 0.016 mg/L (16 μg/L) and 0.04 mg/L (40 μg/L), respectively, for non-cancer effects resulting from exposure to bromate in drinking water were calculated. The cancer risk assessment leads to a MAC that is protective of human health from both cancer and non-cancer effects. The MAC of 10 µg bromate/L takes into consideration limitations in analytical methodology and treatment technologies.

The approach in Europe is largely to follow WHO although the European Commission has toxicological advisory committees that can propose modifications to the WHO approach for a particular substance. The European Commission (EC) adopted in the Drinking Water Directive a value of 10 µg/L based on a balance between the potential risks and the practical application of ozonation as a drinking water treatment process. In the EC´s proposal adopted on 1 February 2018 for a revised drinking water directive, the parametric value of bromate was not modified.

**S 5.2 Inhalation exposure**

The data for inhalation toxicity of bromate were assessed by U.S. EPA as unsuitable for deriving a Reference Concentration (RfC). Likewise, U.S. EPA could not perform a quantitative cancer risk assessment for inhalation exposure due to the lack of appropriate data.

**Tables (Supplementum)**

**Tab. S 1** In vivo and in vitro data on genotoxicity. Core and indicator tests.

1. Core tests

| **In vitro / in vivo ?** | **Endpoint (assay)** | **Model system** | **Concen-tration of KBrO_3_** | **Treatment schedule** | **Result** | **Comments** | **Indications for threshold within the dose ranged analyzed (yes / no)** | **Klimisch category** | **References / institution** |
| --- | --- | --- | --- | --- | --- | --- | --- | --- | --- |
| In vitro  (bacteria) | Ames test | *Salmonella* | 10, 30, 200, 600 µg/plate +/- S9 mix | Treatment for 48 h at 37°C | Negative |  | N/A | 3 | (Dongmei et al. 2015) |
| In vitro  (bacteria) | Ames test | *Salmonella* | 3 mg/plate  + S9 mix | Treatment for 48 h at 37°C | KBrO_3_ positive in Ames test |  | N/A | 3 | (Ishidate et al. 1984) National Institute of Hygienic Sciences, Tokyo, Japan |
| In vitro  (cells) | MN | AS52 CHO cells | 2-10 mM | Treatment for 15 min, MN scored 24 h later | Increase in MN frequency at 2 mM |  | No | 3 | (Ballmaier und Epe 2006) University of Mainz, Germany |
| In vitro  (cells) | MN | HepG2 cells | 0.12-1 mM | Treatment for 24 h | Significant increase in MN at 0.12 mM |  | No | 3 | (Zhang et al. 2011) Dalian Medical University |
| In vitro  (cells) | MN | Human lymphoblastoid AHH1 cells | 0.1-0.8 mM | 4-h treatment + 22 h with cytochalasin B | POD at 0.35 mM according to Hockey-stick model; visible increase from 0.3 - 0.4 mM | Data reanalyzed by (Spassova, Miller et al. 2013) | Conclusion by authors: yes;  Conclusiion by (Spassova, Miller et al. 2013): data consistent with low dose linearity | 3 | (Seager et al. 2012) |
| In vitro  (cells) | MN | Human lymphoblastoid TK6 cells | 0.5-5 mM | Treatment for 4 h | Increase at 0.5 mM; dose-response dependent induction | At 5 mM, MN frequencies were 30 times above background levels | No | 3 | (Luan et al. 2007) National Institute of Health Sciences, Tokyo, Japan |
| In vitro  (cells) | MN | Human lymphoblastoid TK6 cells | 0.05-5 mM | Short-term treatment followed by recovery time (1+23 h, 2+22 h, 3+21 h, 6+18 h); 24-h long-term treatment | Non-linear dose-effect relationships starting with non-mutagenic low doses (<0.1 mM depending on schedule) and going stepwise up to higher doses (up to 5 mM);  in case of 24-h long-term treatment, increase in mutagenicity at 0.05 mM | After short-term exposure, 3 different plateaus were observed suggesting complex dose-dependent activations and interactions of different cellular mechanisms;  data reanalyzed by (Spassova, Miller et al. 2013) | Conclusion by authors: yes;  Conclusion by (Spassova, Miller et al. 2013): data consistent with low dose linearity | 3 | (Platel et al. 2009) Institut Pasteur de Lille, France |
| In vitro  (cells) | MN | Human peripheral lymphocytes | 400-550 µg/ml | Treatment for 24 and 48 h | Significant increase ≥ 500 µg/ml at 24 h; significant increase at ≥ 400 µg/ml at 48 h |  | N/A | 3 | (Kaya und Topaktaş 2007) Cukurova University, Turkey |
| In vitro  (cells) | MN | Primary human and rat kidney cells | 0.56-1.8 mM | Treatment for 48 h | Significant increase in MN at 0.56 mM | Similar levels in rat and human cells | No | 2 | (Robbiano et al. 1999) University of Genoa, Italy |
| In vitro  (cells) | Mutagenicity (HPRT) | V79 Chinese hamster cells | 5-200 mM | Treatment for 1 h | Increase after 5 mM treatment | High proportion of deletion mutations. 3 out of 4 point mutations were G-T transversions (typical for replication after 8-oxo-dG) | No | 3 | (Speit et al. 1999) University of Ulm, Germany |
| In vitro  (cells) | Mutagenicity (GPT) | AS52 CHO cells | 20-100 mM | Treatment for 15 min | Significant increases in FPG sensitive sites | Correlation analysis between FPG-sensitive sites and mutation frequencies | No | 3 | (Ballmaier und Epe 2006) University of Mainz, Germany |
| In vitro  (cells) | Mutagenicity (TK and HPRT) | Mouse lymphoma L5178Y | 1-4 mM | Treatment for 3 h | Increase in mutation frequencies in the *Tk* and *Hprt* loci ≥ 0.5 mM |  | No | 3 | (Priestley et al. 2010) AstraZeneca, Cheshire, UK |
| In vitro  (cells) | Mutagenicity (TK) | L5178Y mouse lymphoma cell line | 0.06-3 mM | Incubation for 4 h at 37°C | Mutation frequency > 100 x 10^-6^ at 0.6 mM with minimal cytotoxicity (70-80% survival) and > 1300 x 10^-6^ at 3 mM (~10% survival) | Mechanism of action, primarily via loss of heterozygosity;  data reanalyzed by (Spassova, Miller et al. 2013) | Conclusion by (Spassova, Miller et al. 2013): data consistent with low dose linearity | 3 | (Harrington-Brock et al. 2003) US EPA, USA |
| In vitro  (cells) | Mutagenicity (TK) | Human lymphoblastoid TK6 cells | 0.75-5 mM | 1-h treatment | Statistically significant increase at 2 mM |  | Yes | 3 | (Platel et al. 2011) University Lille Nord de France |
| In vitro  (cells) | Mutagenicity (TK) | Human lymphoblastoid TK6 cells | 0.5 - 5 mM | Treatment for 4 h | Tendency of increase at 0.5 mM, statistically significant at 5 mM;  dose-response dependent induction | At 5 mM KBrO_3_, TK mutation frequencies were 30 times the background level;  90% of the induced mutations were large deletions that involved LOH;  major genotoxicity may be due to DSBs that lead to large deletions rather than to 8-oxo-dG adducts that lead to GC->TA transversions | No | 3 | (Luan et al. 2007) National Institute of Health Sciences, Tokyo, Japan |
| In vitro  (cells) | Mutagenicity (HPRT) | Human lymphoblastoid AHH1 cells | 0.1 - 0.8 mM | 24-h treatment | POD at 0.18 mM according to Hockey-stick model; visible increase from 0.25 mM | Data reanalyzed by (Spassova, Miller et al. 2013) | Conclusion by authors: yes;  Conclusion by (Spassova, Miller et al. 2013): data consistent with low dose linearity | 3 | (Seager et al. 2012) |
| In vivo | BrdU labeling indices | Rat (Gpt delta), kidney | 500 ppm (drinking water) | KBrO_3_ in drinking water for 9 wks, with 1% of alpha-TP or SAA administered in the diet from 1 wk prior to the KBrO_3_ treatment | BrdU-LIs in the proximal tubules of female rats significantly reduced by SAA, but not those in males | IHC and WB analyses suggested that induction of cell proliferation observed in males might primarily result from the accumulation of α2U-globulin, independent of oxidative stress | N/A | 3 | (Umemura et al. 2009) National Institute of Health Sciences, Japan |
| In vivo | BrdU labelling indices | Rat (male and female), kidney | 300 mg/kg (i.g.) 80 mg/kg (i.p.)  15, 30, 60, 125, 250, 500 ppm (drinking water) | 48 h after administration (i.g., i.p.)  4 wks (oral) | Increase in BrdU LI for proximal convoluted tubules at ≥30 ppm in males and at ≥250 ppm in females | α2u-globulin accumulation in the kidneys of male rats was increased at ≥125 ppm | Yes | 3 | (Umemura et al. 2004) National Institute of Health Sciences, Tokyo, Japan |
| In vivo | MN (bone marrow) | Mouse (male and female) | Mouse: 100 – 2150 mg/kg bw (o. g.) | 14 d observation period | Negative | Negative test results in Ames test and mouse sperm abnormality test, as well | Negative test results | 3 | (Dongmei et al. 2015) |
| In vivo | MN (erythrocyte and spermatide) | Mouse (male) | 80-800 ppm (drinking water) | Sampling after 8 wks and 78 wks | At 800 ppm, MN frequencies were ≤ 4 times above control levels after 8 wks and ≤ 2 times above control levels after 78 wks; at 80 ppm, a significant elevation in MN frequency was observed only after 8 wks.  No effects on germ cells | Same cohort as used in DeAngelo [1998];  data reanalyzed by (Spassova, Miller et al. 2013) | No Conclusion by (Spassova, Miller et al. 2013): data consistent with low dose linearity | 2 | (Allen et al. 2000) US-EPA, USA |
| In vivo | MN (reticulocytes) | Mouse (male), peripheral blood reticulocytes | 37.5 - 212 mg/kg bw (i.p.) (NGTR); 18.8 – 212 mg/kg bw (i.p.) (OPF) | NGTR: 0, 24, 48, 72, 96 h; OPF: 0, 24, 48, 72, 96 h | NGTR: Significant increase in M- at 37.5 mg/kg bw after 48 h; OPF: Significant increase at 37.5 mg/kg bw after 24 h; no significant increase at 18.8 mg/kg bw | Data reanalyzed by (Spassova, Miller et al. 2013) | Conclusion by (Spassova, Miller et al. 2013): data consistent with low dose linearity | 3 | (Awogi et al. 1992) Otsuka Pharmaceutical Factory (OPF) Nippon Glaxo (NGTR), Japan |
| In vivo | MN (kidney cells) | Rat (male) | 160 mg/kg (½ LD50) or 107 mg/kg (1/3 LD50) | 160 mg/kg p.o., single dose 107 mg/kg p.o. for 3 successive days | Significant increase in MN with both treatment schedules |  | N/A | 2 | (Robbiano et al. 1999) University of Genoa, Italy |
| In vivo | MN (liver, stomach, colon, and bone marrow) | Rat (male) | 40, 60, 80 mg/kg (i.g.) | Treatment for 4, 14, 28 d | Increased frequency of MN in stomach and bone marrow, but not in liver and colon |  | No | 2 | (Okada et al. 2015)  Yakult Honsha Co. Ltd. Tokoy, Japan Collaborative study by the Mammalian Mutagenicity Study group (Subgroup of the Japanese Environmental Mutagen Society) |
| In vivo | MN (reticulocytes) | Rat (male), peripheral blood reticulotcytes | 40, 60, 80 mg/kg bw (i.p.) | Analyses up to 55 h post injection;  co-treatment with GSH or Cys i.p. at doses of 8,000 mg/kg and 400 mg/kg, respectively 30 min before and 30 min after KBrO_3_ treatment | Peak in MN formation 32 h after treatment;  co-treatment with GSH or Cys i.p. significantly inhibited MN formation | Results indicate that antioxidants have protective potential against the clastogenicity of KBrO_3_ | No | 3 | (Sai, Hayashi et al. 1992) National Institute of Hygienic Sciences, Tokyo, Japan |
| In vivo | Mutagenicity (GPT) [combined with 8-oxo-dG] | Mouse (WT and *Nrf2* KO) | 1500 ppm (drinking water) | Treatment for 4 or 13 wks | Time-dependent increase in deletion mutations accompanied by an increase in 8-oxo-dG levels |  | N/A | 3 | (Tsuchiya et al. 2018) National Institute of Health Sciences, Kanagawa, Japan |
| In vivo | Mutagenicity (GPT) | Mouse (WT and *Ogg1* KO), kidney | 2 g/l (drinking water) | Treatment for 12 wks | Increase in mutation frequency after 12 wks in wt and OGG1 KO mice. |  | No | 3 | (Arai et al. 2002) Banyu Tsukuba Research Institute, Tsukuba, Japan |
| In vivo | Mutagenicity (GPT) | Mouse (WT and OGG1), liver | 2 g/l (drinking water) | Treatment for 12 wks | Higher mutation frequency in treated *Ogg1* KO mice |  | N/A | 3 | (Arai et al. 2003)  Banyu Tsukuba Research Institute, Tsukuba, Japan |
| In vivo | Mutagenicity (Big Blue rat) combined with 8-oxo-dG | Rat (Big Blue), kidney | 125-500 ppm (drinking water) | Treatment for 16 wks | 500 ppm significantly increased mutants and mutation frequencies;  doses ≤ 125 ppm had no effects;  8-oxo-dG was significantly increased at doses ≥500 ppm | Authors conclude that data points to no-effect levels for in vivo mutagenic and toxic effects, proliferation stimulus and oxidative stress of KBrO_3_ in rat kidney;  8-oxo-dG data reanalyzed by (Spassova, Miller et al. 2013) | Conclusion by authors: yes;  Conclusion by (Spassova, Miller et al. 2013): data consistent with low dose linearity | 2 | (Yamaguchi et al. 2008) Osaka City University Medical School |
| In vivo | Mutagenicity (GPT) | Rat (*Gpt* delta), kidney | 60, 125, 250, 500 ppm (drinking water) | 13 wks;  in a 2-stage experiments; rats were given KBrO_3_ for 13 wks and after a 2-wks recovery; treated with 1% NTA in the diet for 39 wks | Elevation of Spi^-^ mutant frequency, suggestive of deletion mutations, occurred after 9 wks at 500 ppm | Incidence and multiplicity of renal preneoplastic lesions in rats at 500 ppm KBrO_3_ followed by NTA treatment were significantly higher than in rats treated with NTA alone;  mutagenicity data reanalyzed by (Spassova, Miller et al. 2013) | Conclusion by authors: yes;  Conclusion by (Spassova, Miller et al. 2013): data consistent with low dose linearity | 3 | (Umemura et al. 2006)  National Institute of Health Sciences, Tokyo, Japan |
| In vivo | Mutagenicity (GPT) | Rat (*Gpt* delta), kidney | 500 ppm (drinking water) | KBrO_3_ in drinking water for 9 wks, with 1% of alpha-TP or SAA administered in the diet from 1 wk prior to the KBrO_3_ treatment until the end of the experiment | Gpt mutant frequencies of both sexes were not affected by SAA or alpha-TP. | IHC and WB analyses suggested that induction of cell proliferation observed in males might primarily result from the accumulation of α2U-globulin, independent of oxidative stress | N/A | 3 | (Umemura et al. 2009) National Institute of Health Sciences, Japan |
| In vivo | Other (DNA-protein crosslinking) | Rat (male), intestine | KBrO_3_ (100 mg/kg/d) and taurine (100 mg/kg/d for 5 d) (oral) | Animals were sacrificed 48 h after treatment | Increase in DNA damage and crosslinking after KBrO_3_ treatment; protective effects of taurine on these parameters |  | N/A | 3 | (Ahmad et al. 2015)  Aligarh Muslim University, Aligarh, India |
| In vivo | Other (DNA-protein crosslinks) | Rat (male), intestine | 100 mg/kg bw (single oral dose) | Animals sacrificed after 12, 24, 48, 96, 168 h | Significant increase in DNA damage after 12 h; maximum induction of DNA damage after 48 h; repair of DNA damage was still incomplete after 168 h |  | N/A | 3 | (Ahmad et al. 2013) Aligarh Muslim University, Aligarh, India |
| In vivo | Other (sperm cell abnormality test) | Mouse (male) | 100 – 2150 mg/kg bw (oral gavage) | 14 d observation time | Negative |  | N/A | 3 | (Dongmei et al. 2015) |

1. Indicator tests

| **In vitro / in vivo ?** | **Endpoint (assay)** | **Model system** | **Concen-tration of KBrO_3_** | **Treatment schedule** | **Result** | **Comments** | **Indications for threshold (yes / no)** | **Klimisch category** | **References / institution** |
| --- | --- | --- | --- | --- | --- | --- | --- | --- | --- |
| In vitro (DNA) | 8-oxo-dG (HPLC) | CT-DNA | 1.5 mM | Treatment for 15 min | Significant increase only in the presence of GSH and NACs |  | N/A | 3 | (Parsons und Chipman 2000) University of Birmingham, UK |
| In vitro (DNA) | 8-oxo-dG (HPLC, FPG) | PM2 DNA | 0.1-1 mM | Treatment for 15 min | Increase in FPG sensitive sites at 100 µM | DNA damage only observed in the presence of glutathione | No | 3 | (Ballmaier und Epe 1995) University of Würzburg, Germany |
| In vitro (DNA) | Strand breaks (incision assay) | PM2 DNA | 0.1-1 mM | Treatment for 15 min | Increase at 500 µM (much less than in the presence of FPG) | DNA damage only observed in the presence of glutathione | No | 3 | (Ballmaier und Epe 1995) University of Würzburg, Germany |
| In vitro (DNA) | Strand breaks (SSBs and glycosylase sensitive sites, (alkaline elution) | PM2 DNA | DNA: 0.1-1 mM | Treatment for 15 min (± 2 mM GSH) | PM2 DNA: Increase at 0.1 mM |  | No | 3 | (Ballmaier und Epe 2006) University of Mainz, Germany |
| In vitro  (cells) | 8-oxo-dG (FPG comet / HPLC) | V79 Chinese hamster cells | 1-20 mM | Treatment for 1 h | Increase after 1 mM treatment (FPG comet);  increase after 100 mM (HPLC) |  | No | 3 | (Speit et al. 1999) University of Ulm, Germany |
| In vitro  (cells) | 8-oxo-dG (FPG) | L1210 mouse leukemia cells / LLC-PK1 porcine kidney cells | 7.5 mM in the presence of 2mM GSH | Treatment for 15 min | Strong increase in FPG-sensitive sites / small increase in strand breaks | Higher levels in LLC-PK1 cells than in L1210 cells | N/A | 3 | (Ballmaier und Epe 1995) University of Würzburg, Germany |
| In vitro  (cells) | 8-oxo-dG  (OGG1/FPG comet) | Mouse lymphoma L5178Y | 0.5-7.5 mM | Treatment for 3-44 h | FPG-sensitive sites detectable at 0.5 mM Decline of OGG1-sensitive sites after 21 h, back to basal levels after 44 h (at 4 mM);  damage detection also in alkaline and neutral comet (w/o OGG1/FPG) |  | No | 3 | (Priestley et al. 2010) AstraZeneca, Cheshire, UK |
| In vitro  (cells) | 8-oxo-dG (HPLC) | Rat renal proximal tubules / renal nuclei | 2-5 mM | Incubation up to 4 h | Significant increase after 2 h (first time point) with 2 mM | Conclusion that lipid peroxidation plays a role in the reaction mechanism | No | 3 | (Sai et al. 1994) National Institute of Health Sciences, Japan |
| In vitro  (cells) | 8-oxo-dG (HPLC-ECD) | Human leukemia HL-60 cells; H_2_O_2_-resistant clone, HP100 | 0.5-2 mM | Treatment for 4 h | Significant increase at 0.5 mM in both cell lines; GSH depletion led to slightly lower 8-oxo-dG levels | Also studies with CT-DNA +/- antioxidants and GSH;  conclusion: reduction of KBrO_3_ by intracellular SH compounds yields bromine oxides and bromine radicals, which cause guanine oxidation | No | 3 | (Murata et al. 2001) Kyoto University, Japan |
| In vitro  (cells) | 8-oxo-dG (comet, +/- OGG1/FPG) | Human lymphoblastoid TK6 cells | 1-5 mM | Treatment for 1 h, followed or not by a 23-h recovery period | FPG-sensitive sites at 1 mM Strand breaks and OGG1-sensitive sites at 3 mM | Evidence for a thresholded dose-response; NOGELs should be only defined for the induction of chromosomal aberrations and gene mutations, i.e. for an effect endpoint, but not for primary DNA damage, i.e. for an exposure endpoint | Yes | 3 | (Platel et al. 2011) University Lille Nord de France |
| In vitro  (cells) | Strand breaks (comet) | V79 Chinese hamster cells | 1-20 mM | Treatment for 1 h | Increase after 1 mM treatment (comet) | Increase in regular comet much less compared to FPG comet | No | 3 | (Speit et al. 1999) University of Ulm, Germany |
| In vitro  (cells) | Strand breaks (SSBs and glycosylase sensitive sites, alkaline elution) | AS52 CHO cells | AS52 cells: 10-80 mM | Treatment for 15 min (± 2 mM GSH) | Increase at 10 mM |  | No | 3 | (Ballmaier und Epe 2006) University of Mainz, Germany |
| In vitro  (cells) | Strand breaks (comet)) | Mouse lymphoma L5178Y | 0.5 - 7.5 mM | Treatment for 3-44 h | FPG-sensitive sites detectable at 0.5 mM Decline of OGG1-sensitive sites after 21 h, back to basal levels after 44 h (at 4 mM);  damage detection also in alkaline and neutral comet (w/o OGG1/FPG) |  | No | 3 | (Priestley et al. 2010) AstraZeneca, Cheshire, UK |
| In vitro  (cells) | Strand breaks (comet, +/- OGG1/FPG) | Human lymphoblastoid TK6 cells | 1 - 5 mM | 1-h treatment, followed or not by a 23-h recovery period | FPG-sensitive sites at 1 mM Strand breaks and OGG1-sensitive sites at 3 mM | Authors conclude that there is evidence for a threshold level; no-observed-genotoxic-effect-levels (NOGELs) should be only defined for the induction of chromosomal aberrations and gene mutations, i.e. for an effect endpoint, but not for primary DNA damage, i.e. for an exposure endpoint | Yes | 3 | (Platel et al. 2011) University Lille Nord de France |
| In vitro  (cells) | Comet (alkaline and neutral) | Human lymphoblastoid TK6 cells | 0.5 - 5 mM | Treatment for 4 h | Significant increase at 1 mM Dose-response dependent induction | Data reanalyzed by (Spassova, Miller et al. 2013) | Conclusion by authors: yes;  conclusion by (Spassova, Miller et al. 2013): data consistent with low dose linearity | 3 | (Luan et al. 2007) National Institute of Health Sciences, Tokyo, Japan |
| In vitro  (cells) | Strand breaks (comet) | Human white blood cells, rat kidney epithelial cells | 5 mM (hWBC); 1.5 mM (rKEC) | hWBC: treatment for 15 min (optional pretreatment with DEM); rKEC: treatment for 15 min up to 24 h | Strand breaks were induced in white blood cells at 5 mM and in rat kidney epithelial cells at 1.5 mM; depletion of intracellular GSH by DEM decreased the strand break levels in human lymphocytes; extracellular GSH protected against strand break induction | Conclusion: DNA damage by KBr_3_O is largely dependent on access to intracellular GSH | N/A | 3 | (Parsons und Chipman 2000)  University of Birmingham, UK |
| In vitro  (cells) | Strand breaks (comet) | Primary cultures of human and rat kidney cells | 0.56-1.8 mM | Treatment for 20 h | Significant damage induction at 0.56 mM | More damage in rat than in human cells | No | 2 | (Robbiano et al. 1999) University of Genoa, Italy |
| In vitro  (cells) | Other (g-H2A.X, IF) | Mouse lymphoma L5178Y | 4 mM | Treatment for 3 h | Increase in staining |  | N/A | 3 | (Priestley et al. 2010) AstraZeneca, Cheshire, UK |
| In vitro  (cells) | Other (cell cycle, p53 response) | Normal rat kidney cells HEK293 cells | 2.4 mM | Treatment for 24 h | G2/M arrest in both cell types; Induction of p53 phosphorylation 2 h after treatment in HEK293; Induction of p21 at 0.3 mM after 24 h in HEK293 |  | N/A | 3 | (Zhang et al. 2011) University of Georgia, USA |
| In vitro  (cells) | Other (gene expression) | Human lymphoblastoid AHH1 cells | 0.2 - 0.8 mM |  | 2-fold induction of BRCA1 and APEX1 gene expression |  | N/A | 3 | (Seager et al. 2012) |
| In vivo | 8-oxo-dG (HPLC-ECD), (+ lipid peroxidation and GSH) | Mouse (male), kidney | 70 mg/kg bw (i.p.); also D-R with 20 – 80 mg/kg bw (i.p.) | Analyses 6, 24, 48, 72, 96 h after i.p. injection; D-R: 48 h after injection | Significant increase of 8-oxo-dG after 24 h, thereafter slight decrease until 96 h; significant increases of 8-oxo-dG at a dose of 40 mg/kg bw |  | Yes | 3 | (Sai et al. 1991) National Institute of Hygienic Sciences, Tokyo, Japan |
| In vivo | 8-oxo-dG (HPLC-ECD) | Mouse (WT and *Nrf2* KO) | 750 and 1500 ppm (drinking water) | Treatment for 52 wks | Levels of 8-oxo-dG were significantly increased in a dose-dependent manner in KBrO_3_-treated mice. Levels were slightly, yet significantly, lower in *Nrf2* KO mice | Neoplastic lesions in the small intestine with accumulation of beta-catenin and cyclin D1;  significant increase in combined incidences of preneoplastic and neoplastic lesions in *Nrf2-/-* mice | N/A | 2 | (Yokoo et al. 2016) National Institute of Health, Tokyo, Japan |
| In vivo | 8-oxo-dG (HPLC-ECD) | Mouse (WT and *Ogg1* KO), kidney | 1-2 g/l (drinking water) | 2 g/l for the first 18 wks and then at 1 g/l for another 11 wks; after termination of treatment mice were kept for another 23 wks | 8-oxo-dG levels in *Ogg1* KO mice were 250-fold higher than in wt mice; when mice were sacrificed at 52 wks, no tumor formation was found in kidney or other organs; also, absence of precancerous foci |  | N/A | 3 | (Arai et al. 2006) Tsukuba Research Institute, Tsukuba, Japan |
| In vivo | 8-oxo-dG (HPLC-ECD) and AP lyase assay | Mouse (WT and *Ogg1* KO), kidney | 2 g/l (drinking water) | Treatment for 12 wks | Levels of 8-oxo-dG in kidney DNA tremendously increased in a time-dependent manner in *Ogg1* KO mice; 70-times higher than in wt mice; accumulated 8-oxo-dG did not decrease 4 wks after end of treatment. |  | No | 3 | (Arai et al. 2002) Banyu Tsukuba Research Institute, Tsukuba, Japan |
| In vivo | 8-oxo-dG  +  cell proliferation | Mouse (WT and *Ogg1*), liver | 2 g/l (drinking water) | Treatment for 12 wks | 8-oxo-dG in treated *Ogg1* KO mice increased 26.1 times compared to WT mice;  accumulation in 8-oxo-dG did not decrease 4 wks after end of treatment |  | N/A | 3 | (Arai et al. 2003)  Banyu Tsukuba Research Institute, Tsukuba, Japan |
| In vivo | 8-oxo-dG (HPLC-ECD)  + cumulating replicating fractions (CRFs) | Rat (female), kidney, liver | 100, 200 or 400 mg/kg bw (i.g.);  500 ppm (drinking water) | Analysis 48 h after single dose administration;  cotreatment with 0.05% EHEN orally for the first 2 wks as an initiator with subsequent administration of KBrO_3_ at a dose of 500 ppm in drinking water for 30 wks | 8-oxo-dG levels in the kidneys were significantly increased at doses of 200 and 400 mg/kg, and this correlated with increases of the CRFs of proximal tubules; no significant changes in liver;  in the promotion assay, the number of atypical tubules, atypical hyperplasias and renal cell tumors were significantly higher in rats treated with KBrO_3_ after EHEN initiation compared to rats given EHEN only; no differences in liver tumors after co-treatment | Data suggest that KBrO_3_ is associated with induction of cell proliferation and associated tumor promoting activity | Yes | 3 | (Umemura et al. 1995) National Institute of Health Sciences, Tokyo, Japan |
| In vivo | 8-oxo-dG (HPLC-ECD) | Rat (*Gpt* delta), kidney | 60, 125, 250, 500 ppm (drinking water) | 13 wks;  in a 2-stage experiments, rats were given KBrO_3_ for 13 wks and after a 2-wks recovery, treated with 1%NTA in the diet for 39 wks. | Increases in 8-oxo-dG occurred after 1 wk at 500 ppm and after 13 wks at 250 ppm. | Incidence and multiplicity of renal preneoplastic lesions in rats given KBrO_3_ at 500 ppm followed by NTA treatment were significantly higher than in rats treated with NTA alone | Yes | 3 | (Umemura et al. 2006)  National Institute of Health Sciences, Tokyo, Japan |
| In vivo | 8-oxo-dG (HPLC-ECD) | Rat (Gpt delta), kidney | 500 ppm (drinking water) | KBrO_3_ in drinking water for 9 wks, with 1% of alpha-TP or SAA administered in the diet from 1 week prior to the KBrO_3_ treatment until the end of the experiment | Increases in 8-oxo-dG levels in kidney DNA of both sexes were significantly inhibited by SAA, but not alpha-TP | IHC and WB analyses suggested that induction of cell proliferation observed in males might primarily result from the accumulation of α2U-globulin, independent of oxidative stress | N/A | 3 | (Umemura et al. 2009) National Institute of Health Sciences, Japan |
| In vivo | 8-oxo-dG (HPLC-ECD)  +  cell proliferation | Rat (male + female),  kidney | 500 ppm (drinking water) | Treatment for 1,2,3,4, 13 wks | Increase in 8-oxo-dG levels in treated females 3 wks after the start of the exposure, with cell proliferation only elevated at the 13-wk time point; In males earlier increase in 8-oxo-dG and proliferation |  | N/A | 3 | (Umemura et al. 1998) National Institute of Health Sciences, Tokyo, Japan |
| In vivo | 8-oxo-dG (HPLC-ECD) | Rat (male and female), kidney | 300 mg/kg (i.g.); 80 mg/kg (i.p.);  15, 30, 60, 125, 250, 500 ppm (drinking water) | 48 h after administration (i.g., i.p.)  4 weeks (oral) | Significant increase in 8-oxo-dG after i.g. and i.p. administration  Significant increase in 8-oxo-dG at concentrations at ≥250 ppm in drinking water | α2-macroglobulin accumulation in the kidneys of male rats was increased at 125 ppm and above | Yes | 3 | (Umemura et al. 2004) National Institute of Health Sciences, Tokyo, Japan |
| In vivo | 8-oxo-dG (immunostaining) | Rat (male and female), kidney | 125 mg/L and 400 mg/L (drinking water) | Treatment for 28 d | Increased 8-oxo-dG levels only at 400 mg/L |  | (Yes) | 2 | (Kolisetty et al. 2013)  University of Georgia, Athens, USA |
| In vivo | 8-oxo-dG (immunostaining) +  TUNEL staining | Rat (male and female), kidney | 11.5, 46, 308 mg/L (drinking water) | Treatment for 28 d | Increased 8-oxo-dG levels at doses ≥ 46mg/L;  TUNEL staining in renal proximal tubules increased in a dose-dependent manner at doses ≥ 11.5 mg/L in female and ≥ 46 mg/L in male rats. |  | N/A | 2 | (Kolisetty et al. 2013) University of Georgia, Athens, USA |
| In vivo | 8-oxo-dG (HPLC-ECD) | Rat (male), kidney | 20 and 100 mg/kg bw (i.p.) | Analysis 24 h after injection | Significant increases at 100 mg/kg KBrO_3_ No change at a dose of 20 mg/kg |  | (Yes) | 3 | (Chipman et al. 1998) University of Birmingham, UK |
| In vivo | 8-oxo-dG (HPLC-ECD) | Rats (male),  kidney/liver | 400 mg/kg bw (i.g.) | 3 - 48 h after treatment | Kidney: Increase until 24 h, slight decrease after 48 h; Liver: slight increase until 48 h |  | N/A | 3 | (Kasai et al. 1987)  National Cancer Center Research Institute, Tokyo, Japan |
| In vivo | 8-oxo-dG (HPLC-ECD) | Rats(male), kidney | 80 mg/kg bw (i.p.) | KBrO_3_ was given to rats pre-treated with melatonin, resveratrol, PBN, vitamin E, butylated hydroxytoluene, or 2-mercaptoethylamine; 8-oxo-dG levels were analyzed 6 h after KBrO_3_ treatment | 8-oxo-dG levels in renal genomic DNA significantly increased by more than 100% after the KBrO_3_ treatment. This increase was completely abolished by the treatment with resveratrol and was partially prevented by melatonin, PBN and vitamin E | These results demonstrate that different antioxidants can prevent the oxidative DNA damage induced in the kidney by the carcinogen KBrO_3_ | N/A | 3 | (Cadenas und Barja 1999) Complutense University, Madrid, Spain |
| In vivo | Strand breaks (comet) | Zebrafish (embryo, 72-h-old) | 49.2 mM | Exposure of embryos for 72 h | Moderate, yet statistically significant increase in tail moment |  | N/A | 3 | (Teixidó et al. 2015) |
| In vivo | Strand breaks (comet) | Rat (male) | 160 mg/kg (½ LD50) or 107 mg/kg (1/3 LD50) | 160 mg/kg p.o., single dose 107 mg/kg p.o., 3 successive days | Significant damage induction only after 3 applications |  | N/A | 2 | (Robbiano et al. 1999) University of Genoa, Italy |
| In vivo | Strand breaks (comet) | Rat (male), intestine | 100 mg/kg bw (single oral dose) | Animals sacrificed after 12, 24, 48, 96, 168 h | Significant increase in DNA damage after 12 h; maximum induction of DNA damage after 48 h; repair of DNA damage was still incomplete after 168 h |  | N/A | 3 | (Ahmad et al. 2013) Aligarh Muslim University, Aligarh, India |
| In vivo | Strand breaks [DNA fragmentation (colorimetric diphenylamine assay, comet assay) | Rat (male), intestine | KBrO_3_ (100 mg/kg/d) and taurine (100 mg/kg/d for 5 d) (oral) | Animals were sacrificed 48 h after treatment | Increase in DNA damage and crosslinking after KBrO_3_ treatment; protective effects of taurine on these parameters |  | N/A | 3 | (Ahmad et al. 2015)  Aligarh Muslim University, Aligarh, India |
| In vivo | BrdU labeling indices | Rat (Gpt delta), kidney | 500 ppm (drinking water) | KBrO_3_ in drinking water for 9 wks, with 1% of alpha-TP or SAA administered in the diet from 1 wk prior to the KBrO_3_ treatment until the end of the experiment | BrdU-LIs in the proximal tubules of female rats were significantly reduced by SAA, but not those in males | IHC and WB analyses suggested that induction of cell proliferation observed in males might primarily result from the accumulation of α2U-globulin, independent of oxidative stress | N/A | 3 | (Umemura et al. 2009) National Institute of Health Sciences, Japan |
| In vivo | BrdU labelling indices | Rat (male and female), kidney | 300 mg/kg (i.g.) 80 mg/kg (i.p.)  15, 30, 60, 125, 250, 500 ppm (drinking water) | 48 h after administration (i.g., i.p.)  4 weeks (oral) | Increase in BrdU labeling indices for proximal convoluted tubules at 30 ppm and above in males and at 250 ppm in females | α2u-globulin accumulation in the kidneys of male rats was increased at 125 ppm and above | Yes | 3 | (Umemura et al. 2004) National Institute of Health Sciences, Tokyo, Japan |
| In vivo | Other (DNA-protein crosslinking) | Rat (male), intestine | KBrO_3_ (100 mg/kg/d) and taurine (100 mg/kg/d for 5 d) (oral) | Animals were sacrificed 48 h after treatment | Increase in DNA damage and crosslinking after KBrO_3_ treatment; protective effects of taurine on these parameters |  | N/A | 3 | (Ahmad et al. 2015)  Aligarh Muslim University, Aligarh, India |
| In vivo | Other (DNA-protein crosslinks) | Rat (male), intestine | 100 mg/kg bw (single oral dose) | Animals sacrificed after 12, 24, 48, 96, 168 h | Significant increase in DNA damage after 12 h; maximum induction of DNA damage after 48 h; repair of DNA damage was still incomplete after 168 h |  | N/A | 3 | (Ahmad et al. 2013) Aligarh Muslim University, Aligarh, India |
| In vivo | Other (sperm cell abnormality test) | Mouse (male) | 100 – 2150 mg/kg bw (oral gavage) | 14 d observation time | Negative |  | N/A | 3 | (Dongmei et al. 2015) |

**Tab. S 2** Average oral water ingestion per day due to swimming in swimming pools or freshwater calculated for different assumptions reported in the literature. R=Range, (U)CI= (Upper) Confidence Interval, GM = geometric mean

| User | Ingestion rate^1^  [mL per hour] | Volume per event  [mL] | Duration per event  [min] | Frequency per year  [a^-1^] | Method / remarks^2^ | References |
| --- | --- | --- | --- | --- | --- | --- |
| 0.375 (0.25-0.5) y | 500^3, 5^ |  | 30 | 13 | bodyweight 6.21 kg; quality considered *poor for ingestion rate* (Q=1), good for duration (Q=3), very good for frequency (Q=4) | RIVM 2006 (ingestion  rate based on WHO 2000^4^ based on Beech (1980) based on Datta, US EPA (1979)) |
| 0.5-<2 y | 50^3^ |  | 30 | 48 | bodyweight 10 kg, ingestion rate based on children of 6-18 years | Anses, 2012 (ingestion rate based on Dufour et al. 2006) |
| Children | | | | | | |
| 4.5 (3-6) y | 500^5^ |  | 60 | 104 | bodyweight 16.3 kg; data quality: *poor for ingestion rate* (Q=1), good for duration (Q=3), very good for frequency (Q=4), *high frequency* | RIVM 2006 (ingestion  rate based on WHO 2000^4^ based on Beech (1980) based on Datta, US EPA (1979)) |
| 6-10 y | **GM: 24** (CI95: 17-33) |  |  |  | n=66, geometric means (GM), | Dufour et al. 2017 |
|  |  | 100^6^ |  |  | *ingestion rate*: 95^th^ percentile for children 90 ml/event; mean 37 ml/event ) | World Health Organisation 2006 (citation of Evans et al., 2001 for ingestion volume) |
| 2-15 y | 50 |  | 60 | 48 | Bodyweight 30 kg | Anses, 2012 (ingestion rate based on Dufour et al. 2006) |
| 11-15 years | **GM: 24** (CI95: 19-30) |  |  |  | n=131, geometric means, | Dufour et al. 2017 |
| < 15 y |  | 51 (CI95: 0.62-200) | 81 (CI95: 24-200) | 24 (CI95: 0-91) | Questionnaire, *also never-swimmers*, exemplary measurement of a mouthful of water; mean, Netherlands, swimming pools, n=1644; | Schets et al., 2011 |
| 6-18y | 49 (R: 0-205, UP97: 120)  m: 60, f: 40 |  |  |  | HBM (24h urine, cyanuric acid), n=41, mean | Dufour et al. 2006 |
|  | 26 (SD: 29, R: 1-106) |  |  |  | HBM (24h urin, cyanuric acid, *low recovery rate*), videography, n=16, | Suppes et al. 2014 |
| 2-15y | 200 |  | 90 | 238 | bodyweight 30 kg, 9 years sportive, n=5 | Anses, 2012 (ingestion rate based on Dufour et al. 2006 (161 ml/h) based on Allen et al. 1982 and WHO, 2000 based on Beech (1980) based on Datta, US EPA (1979) ) |
| 9-17y | 161 |  |  |  | HBM (urin, cyanuric acid), n=5 | Dufour et al. 2006 based on Allen et al. 1982 (amount of cyanuric acid ingested) and Briggle et al. 1981 (cyanuric concentrations in pool water (29.9 µg/ml) and 2 hrs swimming) |
| 9-17y | 322 (78-793) |  |  |  | HBM (urin, cyanuric acid), n=5; *ingestion rate was calculated for 2 hrs* | Dufour et al. 2017 based on Allen et al. 1982 and Briggle et al. 1981 |
| Adults | | | | | | |
|  | 21 (R: 0-71)  m: 29, w: 16 |  |  |  | HBM (24h urine, cyanuric acid), n=12, mean, >18 y; | Dufour et al. 2006 |
|  |  | m: 34 (CI95: 0-170)  f: 23 (CI95: 0-110) | m: 68 (CI95: 19-180)  f: 67 (CI95: 19-170) | m: 13^9^ (CI95: 0-54)  f: 16^9^ (CI95: 0-65) | *Questionnaire (also never-swimmers)*, exemplary measurement of a mouthful of water; mean, Netherlands, swimming pools, n=3674 (m/f about 50%); | Schets et al. 2011 |
|  | 4^7^ (SD 12, R: 0-51) |  |  |  | HBM (24h urin, cyanuric acid, *low recovery rate*), videography, n=19 | Suppes et al. 2014 |
|  | **GM: 12** (CI95: 11-14)  m:16, (CI95: 13-20)  f: 9 (C95: 8-11) |  |  |  | HBM (24h urin, cyanuric acid), n=362, geometric means, >15y;  Duration was not taken from Dufour, because it was directed to 1 hour. | Dufour et al. 2017 |
|  |  | 20-50 |  |  | NN | World Health Organisation 2003 (Addendum, 2009 based on Dufour et al. 2006) |
|  |  | 100-200 |  |  | *Secondary literature, no reference given* | (Chorus et al. 2000) |
|  | 25 |  | 60 | 48 | duration: 70 years occasionnels | Anses, 2012 (based on Dufour et al. 2006) |
|  | 50 |  | 120 | 260 | high performing athletes are not considered; poor for ingestion rate (Q=1), good for duration (Q=3), very good for frequency (Q=4), *parameters for competitive swimmers were taken as ‘worst case’ for adults* | RIVM, 2006 (ingestion rate based on different sources) |
|  | 50 |  | 120 | 260 | bodyweight 65 kg; high performing athletes are not considered; data quality considered as poor for ingestion rate (Q=1), good for duration (Q=3), very good for frequency (Q=4) | RIVM, 2006 based as cited on Mennes (1994, uptake 0 ml) based on Vervoorn (1993, personal communication) |
|  | 200 |  | 120 | 143 | 55 years sportive, ingestion rate derived from n=5 (9-17 y) | Anses, 2012 (based on Dufour et al. 2006 (ingestion rate 161 ml/h), based on Allen et al. 1982; and WHO, 2000 based on Beech (1980) based on Datta, US EPA (1979)) |
|  | 2^7^ (SD: 3, R: 0-9) |  |  |  | HBM (24h urin, cyanuric acid, *low recovery rate*), videography, n=9 | Suppes et al. 2014 |
|  | 200 |  | 300 | 238 | 15 years high level sportive | Anses, 2012 (based on Dufour et al. 2006 based on Allen et al. 1982; Briggle et al. 1981 and WHO, 2000 based on Beech (1980) based on Datta, US EPA (1979) ) |
|  | AM: 10, [GM: 6], UCL95: 35 |  |  |  | HBM (urine, cyanuric acid, LC MS/MS) in accordance to self-reported ingestion), n=27, *age > 6 years* | Dorevitch et al. 2010^8^ |
|  | 14^7^ (SD: 24, R: 0-106) |  |  |  | HBM (24h urin, cyanuric acid), videography, n=35 | Suppes et al. 2014 |
|  | 32, [GM: 14], (CI95 13-16), (R: 0-280) |  |  |  | HBM (24h urin, cyanuric acid), n=549, all ages | Dufour et al. 2017 |

^1^Data is given as arithmetic mean.

^2^For data not considered for further calculations, main reasons are marked in italic.

^3^Anses (2012) suggested an uptake rate of 50 mL / h based on the study of Dufour et al. (2006). The ingestion rate for infants and toddlers from the age of 6 months to 2 years given by Anses (2012) is based on the study of Dufour et al. (2006), who derived a mean ingestion rate of 49 ml per hour in a cyanuric acid human-biomonitoring study. Nevertheless, Dufour et al. (2006) only determined the ingestion rate of 41 children and adolescents between 6 and 18 years. Thus, it cannot be ruled out that younger children might ingest in average more than 50 mL pool water per hour.

^4^The WHO guidelines for safe recreational-waters environments (1998 Vol. 1 and 2000 Vol. 2) were cited. Nevertheless, in the current editions (2003 Vol. 1 and 2006 Vol. 2) the assumption of an intake of 500 ml per hour was not made by WHO anymore. Instead a worst case intake of 100 ml for a child is used, based on the upper 95th percentile intake for children of 90 ml during a swimming event reported in a dichloroisocyanurate human-biomonitoring pilot study with children by Evans et al. (2001) as described in World Health Organisation (2006). Probably, this change was due to a calculation error made by WHO (2000) because they calculate from a six-year-old child taking 5 ml of water with each breath while swimming into the mouth and squirt it out again, and the assumption of 1% to be swallowd and 1000 breaths per hour, that up to 0.5 litres would be ingested, but 5 mL x 1000 breaths / hour x 0.01 are 50 mL per hour, thus, 0.05 litres would be ingested.

^5^The assumptions made by RIVM (2006) result in the highest average daily pool water intake of 1,433 µL/kg bw/d. This is mainly due to the high default value taken for the ingestion rate of 500 mL per hour. The 500 mL/h were justified by information given by the WHO^4^ in their guidelines for safe recreational-waters environments (1998 Vol. 1 and 2000 Vol. 2). Nevertheless, in the current editions (2003 Vol. 1 and 2006 Vol. 2) the assumption of an intake of 500 mL per hour was not made by WHO anymore (see point 4 before). Instead a worst case intake of 100 ml for a child is used, based on the upper 95th percentile intake for children of 90 mL during a swimming event reported in a dichloroisocyanurate human-biomonitoring pilot study with children by Evans et al. (2001) as described in World Health Organisation (2006). If instead of 500 mL/h this more recent assumption of 100 mL is taken the average daily pool water intake is 286.8 µL/kg bw/d.

Furthermore, RIVM (2006) assumed a swimming frequency of 104 times swimming per year, while Anses (2012) assumes 48 times per year for children. As in the risk assessment here the group of sportive children was addressed separately assuming a frequency of 238 times per year, it seemed adequately to assume as long term average frequency for children not more than one pool visit per week. That might be different in countries with a hot climate during the whole year.

^6^The assumption made by World Health Organisation (2006) of an ingestion rate of 100 mL per hour is derived from a worst case scenario for a child, based on the upper 95th percentile intake for children of 90 mL during a swimming event reported in a dichloroisocyanurate human-biomonitoring pilot study with children by Evans et al. (2001) as described in World Health Organisation (2006). As the pool water uptake should reflect a mean daily intake for the calculation of risk-dependent maximum bromate levels and not a worst case scenario as it might be interesting for risk assessment of microbial infections, the 95th percentile seems not be appropriate. The mean ingestion rate given by Evans et al. (2001) was 37 mL/h as reported by World Health Organisation.

^7^Ingestion rates determined by Suppes et al. (2014) were also not further considered because the authors give cause for concern that the cyanuric acid concentrations that have been measured appeared to be strongly underestimated in their study. The recovery rate in urine was only 6% compared to about 100% in earlier studies (e.g. by Allen et al. 1982; Dufour et al. 2017).

^8^The study of Dorevitch et al. (2010) was not taken to derive the average daily pool water ingestion for children or adults because all ages from >6 years were included in the target group and inconsistency with the methods used were reported.

^9^With respect to swimming frequencies per year, the means reported by Schets et al. (2011) are lower than other sources suggest. This is probably due to the target population that was chosen to answer the questionnaire. It included an unknown number of people, who do not swim at all (never swimmers) leading to a lower average frequency and making this parameter unsuitable for the derivation of the average daily pool water ingestion for swimmers.

**Tab. S3** Exposure scenarios for oral water uptake from pool water based on grouping and body weight suggested by ECHA (2017) or ConsExpo.

|  | **Ingestion rate^1^**  **(75^th^/95^th^ percentile oder maximum)**  [mL/h] | **Duration per day^2^**    [h] | **Frequency per year^2^**    [d/a^-1^] | **Body weight**    [kg] | **Average daily pool water uptake per kg body weight and day**    [µL/kg bw/d] |
| --- | --- | --- | --- | --- | --- |
| **Infants (<1 y)** | 216 (95th) | 0.5 | 48 | 8 | 1,775 |
| **Toddlers (1 to <2 y)** | 216 (95th) | 0.5 | 48 | 10 | 1,420 |
| **Children (2 to <6 y)** | 216 (95th) | 1 | 48 | 15.6 | 1,821 |
| **Children (2 to <6 y, sport-active)** | 397 (max.) | 1.5 | 238 | 15.6 | 24,891 |
| **Children (6 to <12 years)** | 216 (95th) | 1 | 48 | 23.9 | 1,189 |
| **Children (6 to <12 years, sport-active)** | 397 (max.) | 1.5 | 238 | 23.9 | 16,247 |
| **Adults** | 27 (75th) | 1 | 48 | 60 | 59 |
| **Adults** (all data according to ConsExpo) | 50 | 1 | 52 | 65 | 110 |
| **Adults (sport-active)** | 397 (max.) | 2 | 143 | 60 | 5,185 |
| **Adults (top athletes)** | 397 (max.) | 5 | 238 | 60 | 21,572 |

^1^for infants, toddlers, children (data from children 6-10y, n=66) and adults according to Dufour et al. (2017) and for sport-active groups (data from 9-17y, n=5) calculated based on Briggle et al. (1981) and Allen et al. (1982), selection of indicative exposure values according to ECHA (2007): 75th percentile for moderate, 95th percentile for considerable and maximum for high data uncertainty,

^2^according to Anses (2012) except for adults with data from ConsExpo

**Tab. S4** Derivation of cancer risk-related bromate concentrations in swimming pool water considering the main uptake via oral route (see **Tab. S3**) and a value of 65 and 6.5 ng/kg bw/d for an additional theoretical life-long cancer risk of 1:100,000 and 1:1,000,000 derived from the hBMDL_10_.

|  | **Volume per kg body weight and day^1^**  [µl/kg bw /d] | **Additional theoretical lifelong cancer risk of 1:100,000^2^**  [ng Bromate / kg bw /d] | **Bromate concentration in swimming water for a risk of 1:100,000**  [µg/L] | **Bromate concentration in swimming water for a risk of 1:1,000,000**  [µg/L] |
| --- | --- | --- | --- | --- |
| **Infants (<1 y)** | 1,775 | 65 | 37 | 3.7 |
| **Toddlers (1 to <2 y)** | 1,420 | 65 | 46 | 4.6 |
| **Children (2 to <6 y)** | 1,821 | 65 | 36 | 3.6 |
| **Children (2 to <6 y, sport-active)** | 24,891 | 65 | 3 | 0.3 |
| **Children (6 to <12 years)** | 1,189 | 65 | 55 | 5.5 |
| **Children (6 to <12 years, sport-active)** | 16,247 | 65 | 4 | 0.4 |
| **Adults** | 59 | 65 | 1098 | 110 |
| **Adults** (all data according to ConsExpo) | 110 | 65 | 593 | 59 |
| **Adults (sport-active)** | 5,185 | 65 | 13 | 1.3 |
| **Adults (top athletes)** | 21,572 | 65 | 3 | 0.4 |

^1^as calculated in **Tab. S3**
^2^based on the hBMDL_10_ of 0.65 mg bromate /kg bw/d as calculated in **Tab. 10.** The hBMDL10 represents an additional theoretical life-long cancer risk of 1:10 (10%), lower bound estimate of the confidence interval.

**References (Supplementum)**

Ahmad MK, Khan AA, Ali SN, Mahmood R. 2015. Chemoprotective effect of taurine on potassium bromate-induced DNA damage, DNA-protein cross-linking and oxidative stress in rat intestine. PLoS One. 10:e0119137. Epub 2015 Mar 6. eng. doi:10.1371/journal.pone.0119137.

Ahmad MK, Zubair H, Mahmood R. 2013. DNA damage and DNA-protein cross-linking induced in rat intestine by the water disinfection by-product potassium bromate. Chemosphere. 91:1221–1224. Epub 2013 Feb 4. eng. doi:10.1016/j.chemosphere.2013.01.008.

Allen JW, Collins BW, Lori A, Afshari AJ, George MH, DeAngelo AB, Fuscoe JC. 2000. Erythrocyte and spermatid micronucleus analyses in mice chronically exposed to potassium bromate in drinking water. Environ Mol Mutagen. 36:250–253. eng. doi:10.1002/1098-2280(2000)36:3<250:aid-em9>3.0.co;2-6.

Allen LM, Briggle TV, Pfaffenberger CD. 1982. Absorption and excretion of cyanuric acid in long-distance swimmers. Drug Metab Rev. 13:499–516. eng. doi:10.3109/03602538209029992.

Anderson FA. 1994. Final Report on the Safety Assessment of Sodium Bromate and Potassium Bromate. Journal of the American College of Toxicology. 13:400–414. doi:10.3109/10915819409140615.

Anses. 2012. Évaluation des risques sanitaires liés aux piscines. Partie I: piscines réglementées: Avis de l’Afsset Rapport d’expertise collective.

Arai T, Kelly VP, Komoro K, Minowa O, Noda T, Nishimura S. 2003. Cell proliferation in liver of Mmh/Ogg1-deficient mice enhances mutation frequency because of the presence of 8-hydroxyguanine in DNA. Cancer Res. 63:4287–4292. eng.

Arai T, Kelly VP, Minowa O, Noda T, Nishimura S. 2002. High accumulation of oxidative DNA damage, 8-hydroxyguanine, in Mmh/Ogg1 deficient mice by chronic oxidative stress. Carcinogenesis. 23:2005–2010. eng. doi:10.1093/carcin/23.12.2005.

Arai T, Kelly VP, Minowa O, Noda T, Nishimura S. 2006. The study using wild-type and Ogg1 knockout mice exposed to potassium bromate shows no tumor induction despite an extensive accumulation of 8-hydroxyguanine in kidney DNA. Toxicology. 221:179–186. Epub 2006 Feb 21. eng. doi:10.1016/j.tox.2006.01.004.

Awogi T, Murata K, Uejima M, Kuwahara T, Asanami S, Shimono K, Morita T. 1992. Induction of micronucleated reticulocytes by potassium bromate and potassium chromate in CD-1 male mice. Mutat Res. 278:181–185. eng. doi:10.1016/0165-1218(92)90231-n.

Ballmaier D, Epe B. 1995. Oxidative DNA damage induced by potassium bromate under cell-free conditions and in mammalian cells. Carcinogenesis. 16:335–342. eng. doi:10.1093/carcin/16.2.335.

Ballmaier D, Epe B. 2006. DNA damage by bromate: mechanism and consequences. Toxicology. 221:166–171. Epub 2006 Feb 21. eng. doi:10.1016/j.tox.2006.01.009.

Briggle TV, Allen LM, Duncan RC, Pfaffenberger CD. 1981. High performance liquid chromatographic determination of cyanuric acid in human urine and pool water. J Assoc Off Anal Chem. 64:1222–1226. eng.

Brugger M. 2014. Ozon-Brom-Verfahren zur Aufbereitung von Schwimm- und Badebeckenwasser: Aufnahme des Verfahrens in die DIN 19 643 beantragt. AB Archiv des Badewesen:170–177.

Cadenas S, Barja G. 1999. Resveratrol, melatonin, vitamin E, and PBN protect against renal oxidative DNA damage induced by the kidney carcinogen KBrO3. Free Radic Biol Med. 26:1531–1537. eng. doi:10.1016/s0891-5849(99)00019-2.

Chipman JK, Davies JE, Parsons JL, Nair J, O'Neill G, Fawell JK. 1998. DNA oxidation by potassium bromate; a direct mechanism or linked to lipid peroxidation? Toxicology. 126:93–102. eng. doi:10.1016/s0300-483x(97)00174-1.

Chorus I, Falconer IR, Salas HJ, Bartram J. 2000. Health risks caused by freshwater cyanobacteria in recreational waters. J Toxicol Environ Health B Crit Rev. 3:323–347. eng. doi:10.1080/109374000436364.

DeAngelo AB, George MH, Kilburn SR, Moore TM, Wolf DC. 1998. Carcinogenicity of potassium bromate administered in the drinking water to male B6C3F1 mice and F344/N rats. Toxicol Pathol. 26:587–594. eng. doi:10.1177/019262339802600501.

DIN EN ISO 11206:2013-05. [date unknown]. Wasserbeschaffenheit_- Bestimmung von gelöstem Bromat_- Verfahren mittels Ionenchromatographie (IC) und Nachsäulenreaktion (PCR) (ISO_11206:2011); Deutsche Fassung EN_ISO_11206:2013. Berlin: Beuth Verlag GmbH (11206:2013-05). doi:10.31030/1972886.

Dongmei L, Zhiwei W, Qi Z, Fuyi C, Yujuan S, Xiaodong L. 2015. Drinking water toxicity study of the environmental contaminant--Bromate. Regul Toxicol Pharmacol. 73:802–810. Epub 2015 Oct 22. eng. doi:10.1016/j.yrtph.2015.10.015.

Dorevitch S, Ashbolt NJ, Ferguson CM, Fujioka R, McGee CD, Soller JA, Whitman RL. 2010. Meeting report: knowledge and gaps in developing microbial criteria for inland recreational waters. Environ Health Perspect. 118:871–876. Epub 2010 Jan 25. eng. doi:10.1289/ehp.0901627.

Dufour AP, Behymer TD, Cantú R, Magnuson M, Wymer LJ. 2017. Ingestion of swimming pool water by recreational swimmers. J Water Health. 15:429–437. eng. doi:10.2166/wh.2017.255.

Dufour AP, Evans O, Behymer TD, Cantú R. 2006. Water ingestion during swimming activities in a pool: a pilot study. J Water Health. 4:425–430. eng.

ECHA. 2007. Default human factor values for use in exposure assessments for biocidal products. Recommendation no. 14 of the BPC Ad hoc Working Group on Human Exposure. 8 p.

ECHA. 2017. Human exposure to biocidal products. Technical notes for guidance (TNsG). 102 p.

[EU] European Union. 2006. Regulation (EC) No 1907/2006 of the European Parliament and of the Council.

Fawell J, Walker M. 2006. Approaches to determining regulatory values for carcinogens with particular reference to bromate. Toxicology. 221:149–153. Epub 2006 Feb 8. eng. doi:10.1016/j.tox.2005.12.019.

Harrington-Brock K, Collard DD, Chen T. 2003. Bromate induces loss of heterozygosity in the thymidine kinase gene of L5178Y/Tk(+/-)-3.7.2C mouse lymphoma cells. Mutat Res. 537:21–28. eng. doi:10.1016/s1383-5718(03)00044-5.

Health Canada. 2019. Guidelines for Canadian drinking water quality: Guideline technical document: manganese. Ottawa, ON: Health Canada = Santé Canada. 107 p. ISBN: 9780660074962. www.canada.ca/content/dam/hc-sc/migration/hc-sc/ewh-semt/alt_formats/pdf/pubs/water-eau/sum_guide-res_recom/sum_guide-res_recom-eng.pd.

Ishidate M, Sofuni T, Yoshikawa K, Hayashi M, Nohmi T, Sawada M, Matsuoka A. 1984. Primary mutagenicity screening of food additives currently used in Japan. Food Chem Toxicol. 22:623–636. eng. doi:10.1016/0278-6915(84)90271-0.

Kasai H, Nishimura S, Kurokawa Y, Hayashi Y. 1987. Oral administration of the renal carcinogen, potassium bromate, specifically produces 8-hydroxydeoxyguanosine in rat target organ DNA. Carcinogenesis. 8:1959–1961. eng. doi:10.1093/carcin/8.12.1959.

Kaya FF, Topaktaş M. 2007. Genotoxic effects of potassium bromate on human peripheral lymphocytes in vitro. Mutat Res. 626:48–52. Epub 2006 Nov 22. eng. doi:10.1016/j.mrgentox.2006.08.006.

Kolisetty N, Bull RJ, Muralidhara S, Costyn LJ, Delker DA, Guo Z, Cotruvo JA, Fisher JW, Cummings BS. 2013. Association of brominated proteins and changes in protein expression in the rat kidney with subcarcinogenic to carcinogenic doses of bromate. Toxicol Appl Pharmacol. 272:391–398. Epub 2013 Jun 26. eng. doi:10.1016/j.taap.2013.06.018.

Luan Y, Suzuki T, Palanisamy R, Takashima Y, Sakamoto H, Sakuraba M, Koizumi T, Saito M, Matsufuji H, Yamagata K, et al. 2007. Potassium bromate treatment predominantly causes large deletions, but not GCTA transversion in human cells. Mutat Res. 619:113–123. Epub 2007 Mar 4. eng. doi:10.1016/j.mrfmmm.2007.02.029.

Michalski R, Mathews B. 2007. Occurrence of chlorite, chlorate and bromate in disinfected swimming pool water. [place unknown]: [publisher unknown].

Murata M, Bansho Y, Inoue S, Ito K, Ohnishi S, Midorikawa K, Kawanishi S. 2001. Requirement of glutathione and cysteine in guanine-specific oxidation of DNA by carcinogenic potassium bromate. Chem Res Toxicol. 14:678–685. eng. doi:10.1021/tx000209q.

[OEHHA] Office of Environmental Health Hazard Assessment. 2009. Public Health Goal for Bromate in Drinking Water.

Okada E, Fujiishi Y, Narumi K, Kado S, Wako Y, Kawasako K, Kaneko K, Ohyama W. 2015. Evaluation of repeated dose micronucleus assays of the liver and gastrointestinal tract using potassium bromate: a report of the collaborative study by CSGMT/JEMS.MMS. Mutat Res Genet Toxicol Environ Mutagen. 780-781:94–99. Epub 2014 Mar 14. eng. doi:10.1016/j.mrgentox.2014.03.002.

Parsons JL, Chipman JK. 2000. The role of glutathione in DNA damage by potassium bromate in vitro. Mutagenesis. 15:311–316. eng. doi:10.1093/mutage/15.4.311.

Paweloszek R, Briançon S, Chevalier Y, Gilon-Delepine N, Pelletier J, Bolzinger M-A. 2016. Skin Absorption of Anions: Part Two. Skin Absorption of Halide Ions. Pharm Res. 33:1576–1586. Epub 2016 Mar 21. eng. doi:10.1007/s11095-016-1898-0.

Platel A, Nesslany F, Gervais V, Claude N, Marzin D. 2011. Study of oxidative DNA damage in TK6 human lymphoblastoid cells by use of the thymidine kinase gene-mutation assay and the in vitro modified comet assay: determination of No-Observed-Genotoxic-Effect-Levels. Mutat Res. 726:151–159. Epub 2011 Sep 13. eng. doi:10.1016/j.mrgentox.2011.09.003.

Platel A, Nesslany F, Gervais V, Marzin D. 2009. Study of oxidative DNA damage in TK6 human lymphoblastoid cells by use of the in vitro micronucleus test: Determination of No-Observed-Effect Levels. Mutat Res. 678:30–37. Epub 2009 Jun 24. eng. doi:10.1016/j.mrgentox.2009.06.006.

Priestley CC, Green RM, Fellows MD, Doherty AT, Hodges NJ, O'Donovan MR. 2010. Anomalous genotoxic responses induced in mouse lymphoma L5178Y cells by potassium bromate. Toxicology. 267:45–53. Epub 2009 Oct 22. eng. doi:10.1016/j.tox.2009.10.012.

RIVM. 2006. Disinfectant products fact sheet: To assess the risks for the consumer [RIVM rapport 320005003].

Robbiano L, Carrozzino R, Puglia CP, Corbu C, Brambilla G. 1999. Correlation between induction of DNA fragmentation and micronuclei formation in kidney cells from rats and humans and tissue-specific carcinogenic activity. Toxicol Appl Pharmacol. 161:153–159. eng. doi:10.1006/taap.1999.8796.

Sai K, Takagi A, Umemura T, Hasegawa R, Kurokawa Y. 1991. Relation of 8-hydroxydeoxyguanosine formation in rat kidney to lipid peroxidation, glutathione level and relative organ weight after a single administration of potassium bromate. Jpn J Cancer Res. 82:165–169. eng. doi:10.1111/j.1349-7006.1991.tb01824.x.

Sai K, Tyson CA, Thomas DW, Dabbs JE, Hasegawa R, Kurokawa Y. 1994. Oxidative DNA damage induced by potassium bromate in isolated rat renal proximal tubules and renal nuclei. Cancer Lett. 87:1–7. eng. doi:10.1016/0304-3835(94)90402-2.

Schets FM, Schijven JF, Roda Husman AM de. 2011. Exposure assessment for swimmers in bathing waters and swimming pools. Water Research. 45:2392–2400. Epub 2011 Mar 1. eng. doi:10.1016/j.watres.2011.01.025.

Seager AL, Shah U-K, Mikhail JM, Nelson BC, Marquis BJ, Doak SH, Johnson GE, Griffiths SM, Carmichael PL, Scott SJ, et al. 2012. Pro-oxidant induced DNA damage in human lymphoblastoid cells: homeostatic mechanisms of genotoxic tolerance. Toxicol Sci. 128:387–397. Epub 2012 Apr 26. eng. doi:10.1093/toxsci/kfs152.

Siddiqui MS, Amy GL. 1993. Factors Affecting DBP Formation During Ozone-Bromide Reactions. Journal - American Water Works Association. 85:63–72. doi:10.1002/j.1551-8833.1993.tb05922.x.

Song R, Donohoe C, Minear R, Westerhoff P, Ozekin K, Amy G. 1996. Empirical modeling of bromate formation during ozonation of bromide-containing waters. Water Research. 30:1161–1168. doi:10.1016/0043-1354(95)00302-9.

Speit G, Haupter S, Schütz P, Kreis P. 1999. Comparative evaluation of the genotoxic properties of potassium bromate and potassium superoxide in V79 Chinese hamster cells. Mutation Research/Genetic Toxicology and Environmental Mutagenesis. 439:213–221. doi:10.1016/s1383-5718(98)00200-9.

Suppes LM, Abrell L, Dufour AP, Reynolds KA. 2014. Assessment of swimmer behaviors on pool water ingestion. J Water Health. 12:269–279. eng. doi:10.2166/wh.2013.123.

Teixidó E, Piqué E, Gonzalez-Linares J, Llobet JM, Gómez-Catalán J. 2015. Developmental effects and genotoxicity of 10 water disinfection by-products in zebrafish. J Water Health. 13:54–66. eng. doi:10.2166/wh.2014.006.

Tregear RT. 1966. The permeability of mammalian skin to ions. J Invest Dermatol. 46:16–23. eng. doi:10.1038/jid.1966.4.

Tsuchiya T, Kijima A, Ishii Y, Takasu S, Yokoo Y, Nishikawa A, Yanai T, Umemura T. 2018. Mechanisms of oxidative stress-induced in vivo mutagenicity by potassium bromate and nitrofurantoin. J Toxicol Pathol. 31:179–188. Epub 2018 Jun 2. eng. doi:10.1293/tox.2018-0024.

Umemura T, Kanki K, Kuroiwa Y, Ishii Y, Okano K, Nohmi T, Nishikawa A, Hirose M. 2006. In vivo mutagenicity and initiation following oxidative DNA lesion in the kidneys of rats given potassium bromate. Cancer Sci. 97:829–835. Epub 2006 Jun 29. eng. doi:10.1111/j.1349-7006.2006.00248.x.

Umemura T, Kitamura Y, Kanki K, Maruyama S, Okazaki K, Imazawa T, Nishimura T, Hasegawa R, Nishikawa A, Hirose M. 2004. Dose-related changes of oxidative stress and cell proliferation in kidneys of male and female F344 rats exposed to potassium bromate. Cancer Sci. 95:393–398. eng. doi:10.1111/j.1349-7006.2004.tb03221.x.

Umemura T, Sai K, Takagi A, Hasegawa R, Kurokawa Y. 1995. A possible role for oxidative stress in potassium bromate (KBrO3) carcinogenesis. Carcinogenesis. 16:593–597. eng. doi:10.1093/carcin/16.3.593.

Umemura T, Takagi A, Sai K, Hasegawa R, Kurokawa Y. 1998. Oxidative DNA damage and cell proliferation in kidneys of male and female rats during 13-weeks exposure to potassium bromate (KBrO3). Arch Toxicol. 72:264–269. eng. doi:10.1007/s002040050500.

Umemura T, Tasaki M, Kijima A, Okamura T, Inoue T, Ishii Y, Suzuki Y, Masui N, Nohmi T, Nishikawa A. 2009. Possible participation of oxidative stress in causation of cell proliferation and in vivo mutagenicity in kidneys of gpt delta rats treated with potassium bromate. Toxicology. 257:46–52. Epub 2008 Dec 14. eng. doi:10.1016/j.tox.2008.12.007.

US EPA. 2001. Toxicological Review of Bromate (CAS No. 15541-45-4). Integrated Risk Information System (IRIS).

US EPA. 2021. IRIS Assessment Bromate. [place unknown]: [publisher unknown]; [accessed 2021 May 8]. https://​cfpub.epa.gov​/​ncea/​iris2/​chemicalLanding.cfm​?​substance_nmbr=​1002.

Wasserbeschaffenheit_- Bestimmung von gelöstem Bromat_- Verfahren mittels Ionenchromatographie (ISO_15061:2001); Deutsche Fassung EN_ISO_15061:2001. [date unknown]. Berlin: Beuth Verlag GmbH (15061:2001-12). doi:10.31030/9184167.

World Health Organisation. 2003. Guidelines for safe recreational water environments: Coastal and fresh waters.

World Health Organisation. 2006. Swimming pools and similar environments. Geneva: [publisher unknown]. 118 p. (Guidelines for safe recreational water environments; / World Health Organization ; Vol. 2). ISBN: 9241546808. eng.

World Health Organization. 2017. Guidelines for Drinking-Water Quality: Fourth Edition Incorporating the First Addendum. Geneva: [publisher unknown]. ISBN: 9789241549950.

Yamaguchi T, Wei M, Hagihara N, Omori M, Wanibuchi H, Fukushima S. 2008. Lack of mutagenic and toxic effects of low dose potassium bromate on kidneys in the Big Blue rat. Mutat Res. 652:1–11. Epub 2007 Nov 26. eng. doi:10.1016/j.mrgentox.2007.11.004.

Yokoo Y, Kijima A, Ishii Y, Takasu S, Tsuchiya T, Umemura T. 2016. Effects of Nrf2 silencing on oxidative stress-associated intestinal carcinogenesis in mice. Cancer Med. 5:1228–1238. Epub 2016 Feb 21. eng. doi:10.1002/cam4.672.

Zhang Y, Jiang L, Jiang L, Geng C, Li L, Shao J, Zhong L. 2011. Possible involvement of oxidative stress in potassium bromate-induced genotoxicity in human HepG2 cells. Chem Biol Interact. 189:186–191. Epub 2010 Dec 21. eng. doi:10.1016/j.cbi.2010.12.011.

Zimm BH, Mayer JE. 1944. Vapor Pressures, Heats of Vaporization, and Entropies of Some Alkali Halides. The Journal of Chemical Physics. 12:362–369. doi:10.1063/1.1723958.
