# Supplementary material for: New aspects in deriving health-based guidance values for bromate in swimming pool water
Source: Arch Toxicol. 2022 Apr 6;96(6):1623–59. doi: 10.1007/s00204-022-03255-9 (PMC9095538; doi:10.1007/s00204-022-03255-9)

# Data Description

Kurokawa et al 1983, female animals

The endpoint to be analyzed is: incidence.

Data used for analysis:

| Dose in ppm | incidence | Animal number per group |
| --- | --- | --- |
| 0 | 0 | 47 |
| 250 | 28 | 50 |
| 500 | 39 | 49 |

# Selection of the BMR

The BMR (benchmark response) used is an extra risk of 10% compared to the controls.

The BMD (benchmark dose) is the dose corresponding with the BMR of interest.

A 90% confidence interval around the BMD will be estimated, the lower bound is reported by BMDL and the upper bound by BMDU.

# Software Used

Results are obtained using the EFSA web-tool for BMD analysis, which uses the R-package [PROAST](http://www.rivm.nl/en/Documents_and_publications/Scientific/Models/PROAST), version 66.40, for the underlying calculations.

|  |  |  |
| --- | --- | --- |
|  |  |  |

# Results

## Response variable: incidence

### Fitted Models

| model | No.par | loglik | AIC | accepted | BMDL | BMDU | BMD | conv |
| --- | --- | --- | --- | --- | --- | --- | --- | --- |
| null | 1 | -100.71 | 203.42 |  | NA | NA | NA | NA |
| full | 3 | -59.09 | 124.18 |  | NA | NA | NA | NA |
| two.stage | 3 | -59.10 | 124.20 | yes | 26.5000 | 40.8 | 32.7 | yes |
| log.logist | 3 | -59.09 | 124.18 | yes | 1.7800 | 116.0 | 55.3 | yes |
| Weibull | 3 | -59.09 | 124.18 | yes | 0.2650 | 81.7 | 29.0 | yes |
| log.prob | 3 | -59.09 | 124.18 | yes | 2.0000 | 119.0 | 57.6 | yes |
| gamma | 3 | -59.09 | 124.18 | yes | 0.0145 | 95.1 | 27.9 | yes |
| logistic | 2 | -65.65 | 135.30 | no | NA | NA | 104.0 | yes |
| probit | 2 | -65.14 | 134.28 | no | NA | NA | 100.0 | yes |
| LVM: Expon. m3- | 3 | -59.10 | 124.20 | yes | 4.6900 | 101.0 | 34.8 | yes |
| LVM: Hill m3- | 3 | -59.09 | 124.18 | yes | 2.7700 | 111.0 | 50.4 | yes |

###

### Estimated Model Parameters

**two.stage**

estimate for a- : 1e-06

estimate for BMD- : 32.66

estimate for c : 1e-06

**log.logist**

estimate for a- : 1e-06

estimate for BMD- : 55.27

estimate for c : 1.616

**Weibull**

estimate for a- : 1e-06

estimate for BMD- : 28.99

estimate for c : 0.9529

**log.prob**

estimate for a- : 1e-06

estimate for BMD- : 57.57

estimate for c : 0.9755

**gamma**

estimate for a- : 1e-06

estimate for BMD- : 27.88

estimate for cc : 0.9171

**logistic**

estimate for a- : -2.521

estimate for BMD- : 103.9

**probit**

estimate for a- : -1.547

estimate for BMD- : 100.1

**EXP**

estimate for a- : 2.409

estimate for CED- : 34.83

estimate for d- : 0.25

estimate for th(fixed) : 0

estimate for sigma(fixed) : 0.25

**HILL**

estimate for a- : 4.213

estimate for CED- : 50.4

estimate for d- : 0.3089

estimate for th(fixed) : 0

estimate for sigma(fixed) : 0.25

###

### Weights for Model Averaging

| two.stage | log.logist | Weibull | log.prob | gamma | logistic | probit | EXP | HILL |
| --- | --- | --- | --- | --- | --- | --- | --- | --- |
| 0.14 | 0.14 | 0.14 | 0.14 | 0.14 | 0 | 0 | 0.14 | 0.14 |

### Final BMD Values

| subgroup | BMDL | BMDU |
| --- | --- | --- |
|  | 1.86 | 105 |

Confidence intervals for the BMD are based on 200 bootstrap data sets.

### Visualization
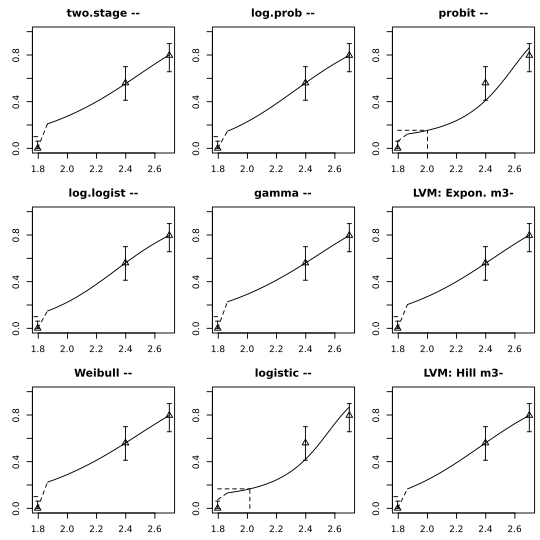

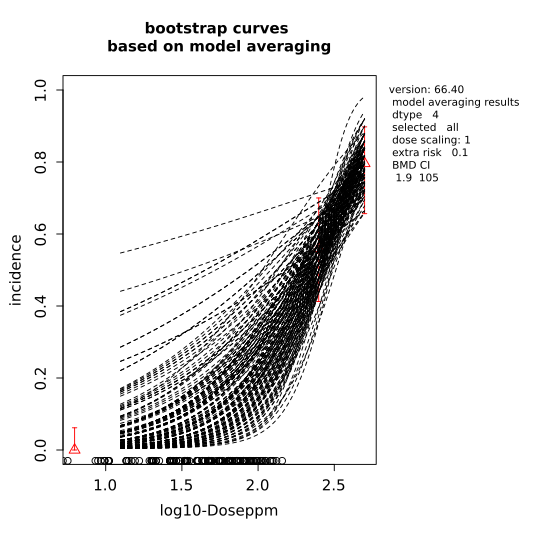

Supplement: Supplementary file 14 — Supplementary file14 (DOCX 159 KB) [file 204_2022_3255_MOESM14_ESM.docx]
