# Supplementary material for: New aspects in deriving health-based guidance values for bromate in swimming pool water
Source: Arch Toxicol. 2022 Apr 6;96(6):1623–59. doi: 10.1007/s00204-022-03255-9 (PMC9095538; doi:10.1007/s00204-022-03255-9)

# Data Description

deAngelo 1998, male animals, renal tumours

The endpoint to be analyzed is: incidence.

Data used for analysis:

| Dose in ppm | incidence | Animal number per group |
| --- | --- | --- |
| 0 | 1 | 45 |
| 20 | 1 | 43 |
| 100 | 6 | 47 |
| 200 | 3 | 39 |
| 400 | 12 | 32 |

# Selection of the BMR

The BMR (benchmark response) used is an extra risk of 10% compared to the controls.

*When the specified BMR deviates from the default value, the rationale behind the choice made should be described.*

The BMD (benchmark dose) is the dose corresponding with the BMR of interest.

A 90% confidence interval around the BMD will be estimated, the lower bound is reported by BMDL and the upper bound by BMDU.

# Results

## Response variable: incidence

### Fitted Models

| model | No.par | loglik | AIC | accepted | BMDL | BMDU | BMD | conv |
| --- | --- | --- | --- | --- | --- | --- | --- | --- |
| null | 1 | -72.09 | 146.18 |  | NA | NA | NA | NA |
| full | 5 | -59.24 | 128.48 |  | NA | NA | NA | NA |
| two.stage | 3 | -61.10 | 128.20 | yes | 88.8 | 256 | 158 | yes |
| log.logist | 3 | -61.33 | 128.66 | no | NA | NA | 150 | yes |
| Weibull | 3 | -61.23 | 128.46 | no | NA | NA | 153 | yes |
| log.prob | 3 | -61.48 | 128.96 | no | NA | NA | 141 | yes |
| gamma | 3 | -61.27 | 128.54 | no | NA | NA | 148 | yes |
| logistic | 2 | -61.13 | 126.26 | yes | 169.0 | 261 | 207 | yes |
| probit | 2 | -61.12 | 126.24 | yes | 155.0 | 251 | 193 | yes |
| LVM: Expon. m3- | 3 | -61.05 | 128.10 | yes | 79.1 | 336 | 168 | yes |
| LVM: Hill m3- | 3 | -61.11 | 128.22 | yes | 78.0 | 333 | 165 | yes |

###

### Estimated Model Parameters

**two.stage**

estimate for a- : 0.02262

estimate for BMD- : 157.6

estimate for c : 6.229

**log.logist**

estimate for a- : 0.02343

estimate for BMD- : 149.9

estimate for c : 1.434

**Weibull**

estimate for a- : 0.02329

estimate for BMD- : 152.7

estimate for c : 1.345

**log.prob**

estimate for a- : 0.02341

estimate for BMD- : 140.8

estimate for c : 0.7264

**gamma**

estimate for a- : 0.02261

estimate for BMD- : 148.3

estimate for cc : 1.38

**logistic**

estimate for a- : -3.336

estimate for BMD- : 206.9

**probit**

estimate for a- : -1.862

estimate for BMD- : 193.1

**EXP**

estimate for a- : 1.639

estimate for CED- : 168

estimate for d- : 0.7776

estimate for th(fixed) : 0

estimate for sigma(fixed) : 0.25

**HILL**

estimate for a- : 1.637

estimate for CED- : 165

estimate for d- : 0.8692

estimate for th(fixed) : 0

estimate for sigma(fixed) : 0.25

###

### Weights for Model Averaging

| two.stage | log.logist | Weibull | log.prob | gamma | logistic | probit | EXP | HILL |
| --- | --- | --- | --- | --- | --- | --- | --- | --- |
| 0.09 | 0.07 | 0.08 | 0.06 | 0.07 | 0.23 | 0.23 | 0.09 | 0.09 |

### Final BMD Values

| subgroup | BMDL | BMDU |
| --- | --- | --- |
|  | 89.4 | 293 |

Confidence intervals for the BMD are based on 200 bootstrap data sets.

### Visualization
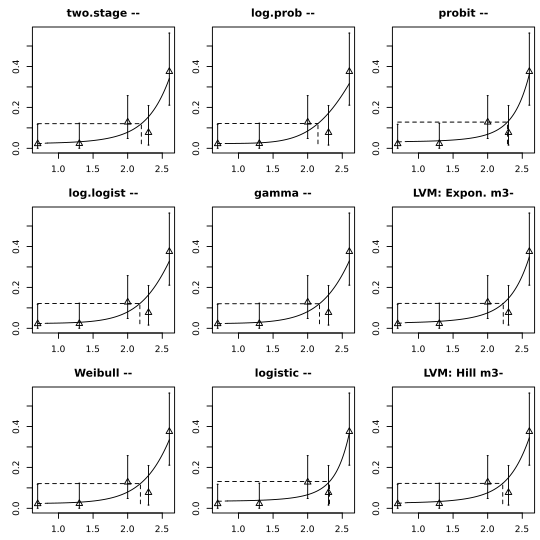

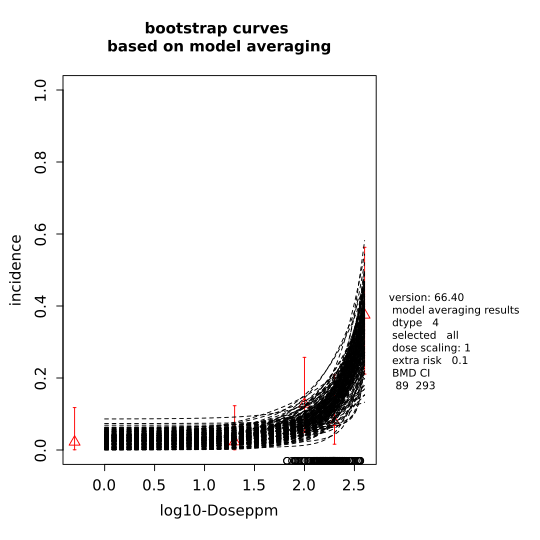

Supplement: Supplementary file 17 — Supplementary file17 (DOCX 110 KB) [file 204_2022_3255_MOESM17_ESM.docx]
