# Supplementary material for: New aspects in deriving health-based guidance values for bromate in swimming pool water
Source: Arch Toxicol. 2022 Apr 6;96(6):1623–59. doi: 10.1007/s00204-022-03255-9 (PMC9095538; doi:10.1007/s00204-022-03255-9)

# Data Description

De Angelo 1998, male animals, renal urothelial hyperplasia

The endpoint to be analyzed is: incidence.

Data used for analysis:

| Dose in ppm | incidence | Animal number per group |
| --- | --- | --- |
| 0 | 7 | 44 |
| 20 | 6 | 41 |
| 100 | 25 | 47 |
| 200 | 32 | 39 |
| 400 | 30 | 32 |

# Selection of the BMR

The BMR (benchmark response) used is an extra risk of 10% compared to the controls.

The BMD (benchmark dose) is the dose corresponding with the BMR of interest.

A 90% confidence interval around the BMD will be estimated, the lower bound is reported by BMDL and the upper bound by BMDU.

# Software Used

Results are obtained using the EFSA web-tool for BMD analysis, which uses the R-package [PROAST](http://www.rivm.nl/en/Documents_and_publications/Scientific/Models/PROAST), version 66.40, for the underlying calculations.

# Results

## Response variable: incidence

### Fitted Models

| model | No.par | loglik | AIC | accepted | BMDL | BMDU | BMD | conv |
| --- | --- | --- | --- | --- | --- | --- | --- | --- |
| null | 1 | -140.69 | 283.38 |  | NA | NA | NA | NA |
| full | 5 | -94.67 | 199.34 |  | NA | NA | NA | NA |
| two.stage | 3 | -96.07 | 198.14 | no | NA | NA | 17.9 | yes |
| log.logist | 3 | -94.81 | 195.62 | yes | 19.6 | 61.3 | 38.9 | yes |
| Weibull | 3 | -95.70 | 197.40 | yes | 11.0 | 42.8 | 23.8 | yes |
| log.prob | 3 | -94.84 | 195.68 | yes | 19.6 | 60.7 | 38.1 | yes |
| gamma | 3 | -95.48 | 196.96 | yes | 11.6 | 51.7 | 28.2 | yes |
| logistic | 2 | -97.25 | 198.50 | no | NA | NA | 35.2 | yes |
| probit | 2 | -98.41 | 200.82 | no | NA | NA | 35.7 | yes |
| LVM: Expon. m3- | 3 | -96.44 | 198.88 | no | NA | NA | 16.2 | yes |
| LVM: Hill m3- | 3 | -95.96 | 197.92 | no | NA | NA | 20.3 | yes |

###

### Estimated Model Parameters

**two.stage**

estimate for a- : 0.125

estimate for BMD- : 17.86

estimate for c : 0.1221

**log.logist**

estimate for a- : 0.1439

estimate for BMD- : 38.93

estimate for c : 2.115

**Weibull**

estimate for a- : 0.132

estimate for BMD- : 23.83

estimate for c : 1.203

**log.prob**

estimate for a- : 0.1462

estimate for BMD- : 38.14

estimate for c : 1.213

**gamma**

estimate for a- : 0.1366

estimate for BMD- : 28.2

estimate for cc : 1.491

**logistic**

estimate for a- : -1.573

estimate for BMD- : 35.16

**probit**

estimate for a- : -0.9006

estimate for BMD- : 35.67

**EXP**

estimate for a- : 1.333

estimate for CED- : 16.15

estimate for d- : 0.6643

estimate for th(fixed) : 0

estimate for sigma(fixed) : 0.25

**HILL**

estimate for a- : 1.329

estimate for CED- : 20.27

estimate for d- : 0.8307

estimate for th(fixed) : 0

estimate for sigma(fixed) : 0.25

###

### Weights for Model Averaging

| two.stage | log.logist | Weibull | log.prob | gamma | logistic | probit | EXP | HILL |
| --- | --- | --- | --- | --- | --- | --- | --- | --- |
| 0.07 | 0.25 | 0.1 | 0.24 | 0.13 | 0.06 | 0.02 | 0.05 | 0.08 |

### Final BMD Values

| subgroup | BMDL | BMDU |
| --- | --- | --- |
|  | 13.1 | 52 |

Confidence intervals for the BMD are based on 200 bootstrap data sets.

### Visualization
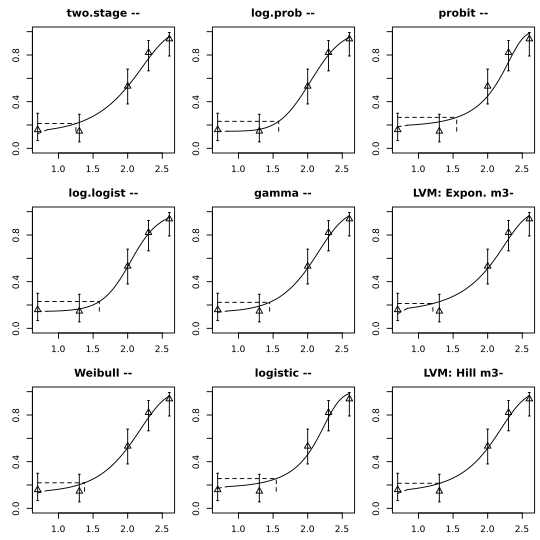

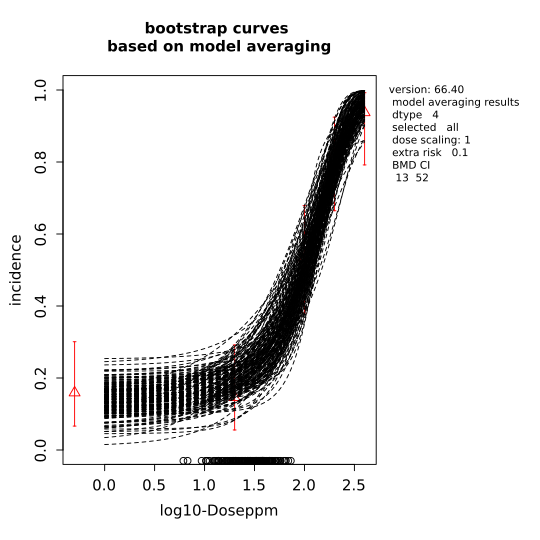

Supplement: Supplementary file 18 — Supplementary file18 (DOCX 141 KB) [file 204_2022_3255_MOESM18_ESM.docx]
