# Supplementary material for: New aspects in deriving health-based guidance values for bromate in swimming pool water
Source: Arch Toxicol. 2022 Apr 6;96(6):1623–59. doi: 10.1007/s00204-022-03255-9 (PMC9095538; doi:10.1007/s00204-022-03255-9)
Supplement: Supplementary file 23 — Supplementary file23 (DOCX 155 KB) [file 204_2022_3255_MOESM23_ESM.docx]

# Data Description

Kurokawa et al. 1983, male animals, renal tumours

The endpoint to be analyzed is: incidence.

Data used for analysis:

| Dose in ppm | incidence | Animal number per group |
| --- | --- | --- |
| 0 | 3 | 53 |
| 250 | 32 | 53 |
| 500 | 46 | 52 |

# Selection of the BMR

The BMR (benchmark response) used is an extra risk of 10% compared to the controls.

The BMD (benchmark dose) is the dose corresponding with the BMR of interest.

A 90% confidence interval around the BMD will be estimated, the lower bound is reported by BMDL and the upper bound by BMDU.

# Software Used

Results are obtained using the EFSA web-tool for BMD analysis, which uses the R-package [PROAST](http://www.rivm.nl/en/Documents_and_publications/Scientific/Models/PROAST), version 66.40, for the underlying calculations.

# Results

## Response variable: incidence

### Fitted Models

| model | No.par | loglik | AIC | accepted | BMDL | BMDU | BMD | conv |
| --- | --- | --- | --- | --- | --- | --- | --- | --- |
| null | 1 | -109.47 | 220.94 |  | NA | NA | NA | NA |
| full | 3 | -65.71 | 137.42 |  | NA | NA | NA | NA |
| two.stage | 3 | -65.71 | 137.42 | yes | 22.70 | 93.2 | 37.0 | yes |
| log.logist | 3 | -65.71 | 137.42 | yes | 22.70 | 138.0 | 86.6 | yes |
| Weibull | 3 | -65.71 | 137.42 | yes | 6.87 | 97.8 | 47.9 | yes |
| log.prob | 3 | -65.71 | 137.42 | yes | 22.30 | 138.0 | 85.8 | yes |
| gamma | 3 | -65.71 | 137.42 | yes | 3.14 | 118.0 | 55.9 | yes |
| logistic | 2 | -67.31 | 138.62 | yes | 64.40 | 105.0 | 81.9 | yes |
| probit | 2 | -67.40 | 138.80 | yes | 61.90 | 97.9 | 77.1 | yes |
| LVM: Expon. m3- | 3 | -65.71 | 137.42 | yes | 3.09 | 76.4 | 32.3 | yes |
| LVM: Hill m3- | 3 | -65.71 | 137.42 | yes | 5.35 | 89.3 | 42.3 | yes |

###

### Estimated Model Parameters

**two.stage**

estimate for a- : 0.0566

estimate for BMD- : 37.02

estimate for c : 0.3909

**log.logist**

estimate for a- : 0.0566

estimate for BMD- : 86.62

estimate for c : 2.378

**Weibull**

estimate for a- : 0.0566

estimate for BMD- : 47.92

estimate for c : 1.276

**log.prob**

estimate for a- : 0.0566

estimate for BMD- : 85.81

estimate for c : 1.387

**gamma**

estimate for a- : 0.0566

estimate for BMD- : 55.9

estimate for cc : 1.613

**logistic**

estimate for a- : -2.261

estimate for BMD- : 81.93

**probit**

estimate for a- : -1.327

estimate for BMD- : 77.13

**EXP**

estimate for a- : 1.486

estimate for CED- : 32.35

estimate for d- : 0.5911

estimate for th(fixed) : 0

estimate for sigma(fixed) : 0.25

**HILL**

estimate for a- : 1.486

estimate for CED- : 42.29

estimate for d- : 0.7759

estimate for th(fixed) : 0

estimate for sigma(fixed) : 0.25

###

### Weights for Model Averaging

| two.stage | log.logist | Weibull | log.prob | gamma | logistic | probit | EXP | HILL |
| --- | --- | --- | --- | --- | --- | --- | --- | --- |
| 0.12 | 0.12 | 0.12 | 0.12 | 0.12 | 0.07 | 0.06 | 0.12 | 0.12 |

### Final BMD Values

| subgroup | BMDL | BMDU |
| --- | --- | --- |
|  | 16.6 | 108 |

Confidence intervals for the BMD are based on 200 bootstrap data sets.

### Visualization
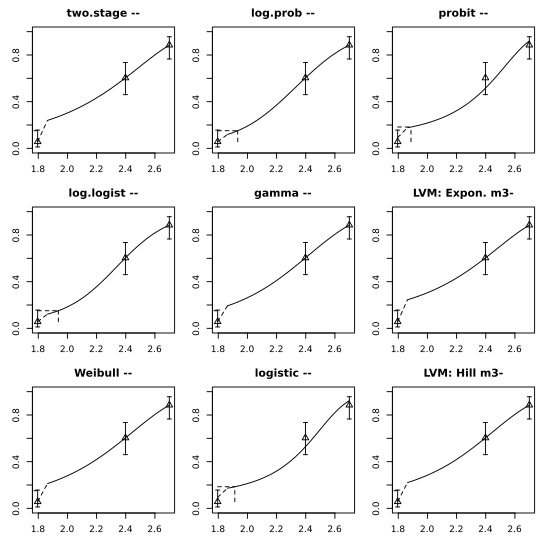

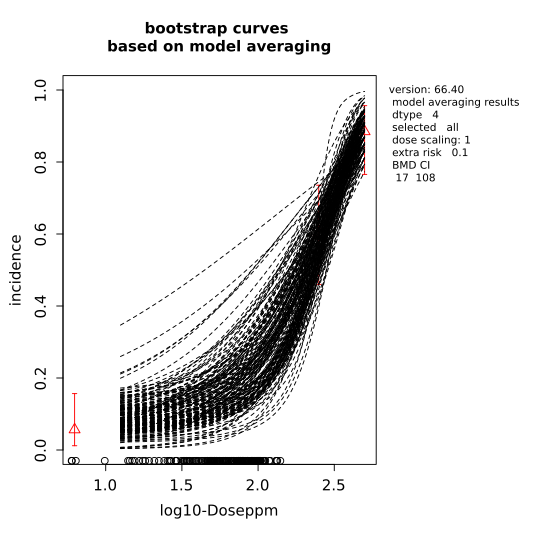


# 
