# Supplementary material for: New aspects in deriving health-based guidance values for bromate in swimming pool water
Source: Arch Toxicol. 2022 Apr 6;96(6):1623–59. doi: 10.1007/s00204-022-03255-9 (PMC9095538; doi:10.1007/s00204-022-03255-9)
Supplement: Supplementary file 26 — Supplementary file26 (DOCX 121 KB) [file 204_2022_3255_MOESM26_ESM.docx]

# Data Description

Kurokawa et al. 1986, male animals, renal tumours

The endpoint to be analyzed is: incidence.

Data used for analysis:

| Dose in ppm | incidence | Animal number per group |
| --- | --- | --- |
| 0 | 0 | 19 |
| 15 | 0 | 19 |
| 30 | 0 | 20 |
| 60 | 1 | 24 |
| 125 | 5 | 24 |
| 250 | 5 | 20 |
| 500 | 9 | 20 |

# Selection of the BMR

The BMR (benchmark response) used is an extra risk of 10% compared to the controls.

The BMD (benchmark dose) is the dose corresponding with the BMR of interest.

A 90% confidence interval around the BMD will be estimated, the lower bound is reported by BMDL and the upper bound by BMDU.

# Software Used

Results are obtained using the EFSA web-tool for BMD analysis, which uses the R-package [PROAST](http://www.rivm.nl/en/Documents_and_publications/Scientific/Models/PROAST), version 66.40, for the underlying calculations.

# Results

## Response variable: incidence

### Fitted Models

| model | No.par | loglik | AIC | accepted | BMDL | BMDU | BMD | conv |
| --- | --- | --- | --- | --- | --- | --- | --- | --- |
| null | 1 | -58.32 | 118.64 |  | NA | NA | NA | NA |
| full | 7 | -41.45 | 96.90 |  | NA | NA | NA | NA |
| two.stage | 3 | -43.08 | 92.16 | yes | 62.7 | 159 | 94.3 | yes |
| log.logist | 3 | -42.74 | 91.48 | yes | 56.5 | 152 | 101.0 | yes |
| Weibull | 3 | -42.93 | 91.86 | yes | 55.2 | 157 | 102.0 | yes |
| log.prob | 3 | -42.39 | 90.78 | yes | 57.9 | 143 | 98.0 | yes |
| gamma | 3 | -42.88 | 91.76 | yes | 56.6 | 155 | 104.0 | yes |
| logistic | 2 | -46.37 | 96.74 | no | NA | NA | 204.0 | yes |
| probit | 2 | -45.85 | 95.70 | no | NA | NA | 187.0 | yes |
| LVM: Expon. m3- | 3 | -42.93 | 91.86 | yes | 59.3 | 156 | 105.0 | yes |
| LVM: Hill m3- | 3 | -42.54 | 91.08 | yes | 59.6 | 147 | 99.4 | yes |

###

### Estimated Model Parameters

**two.stage**

estimate for a- : 1e-06

estimate for BMD- : 94.29

estimate for c : 0.271

**log.logist**

estimate for a- : 1e-06

estimate for BMD- : 100.9

estimate for c : 1.328

**Weibull**

estimate for a- : 1e-06

estimate for BMD- : 102.5

estimate for c : 1.16

**log.prob**

estimate for a- : 1e-06

estimate for BMD- : 98.01

estimate for c : 0.7487

**gamma**

estimate for a- : 1e-06

estimate for BMD- : 103.9

estimate for cc : 1.244

**logistic**

estimate for a- : -3.149

estimate for BMD- : 203.6

**probit**

estimate for a- : -1.829

estimate for BMD- : 187

**EXP**

estimate for a- : 2.677

estimate for CED- : 105.3

estimate for d- : 0.25

estimate for th(fixed) : 0

estimate for sigma(fixed) : 0.25

**HILL**

estimate for a- : 4.482

estimate for CED- : 99.36

estimate for d- : 0.2587

estimate for th(fixed) : 0

estimate for sigma(fixed) : 0.25

###

### Weights for Model Averaging

| two.stage | log.logist | Weibull | log.prob | gamma | logistic | probit | EXP | HILL |
| --- | --- | --- | --- | --- | --- | --- | --- | --- |
| 0.1 | 0.14 | 0.12 | 0.2 | 0.12 | 0.01 | 0.02 | 0.12 | 0.17 |

### Final BMD Values

| subgroup | BMDL | BMDU |
| --- | --- | --- |
|  | 68.9 | 192 |

Confidence intervals for the BMD are based on 200 bootstrap data sets.

### Visualization
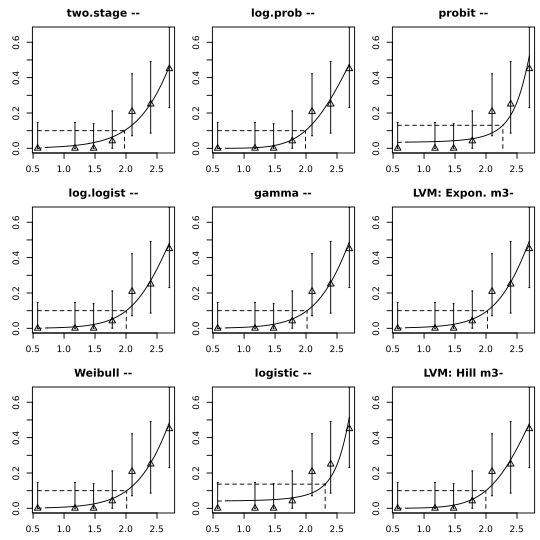

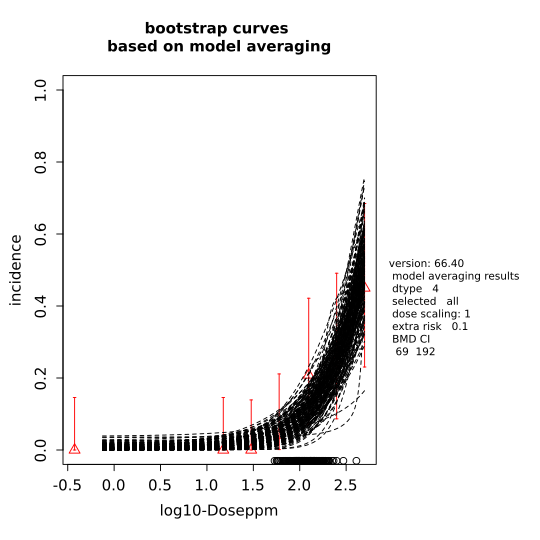


# 
