# Supplementary material for: New aspects in deriving health-based guidance values for bromate in swimming pool water
Source: Arch Toxicol. 2022 Apr 6;96(6):1623–59. doi: 10.1007/s00204-022-03255-9 (PMC9095538; doi:10.1007/s00204-022-03255-9)

# Data Description

The endpoint to be analyzed is: incidence.

Data used for analysis:

| Doseppm | incidence | animalno |
| --- | --- | --- |
| 0 | 0 | 36 |
| 20 | 5 | 39 |
| 100 | 4 | 43 |
| 200 | 6 | 35 |
| 400 | 16 | 30 |

*Information pertaining to this endpoint.*

# Selection of the BMR

The BMR (benchmark response) used is an extra risk of 10% compared to the controls.

*When the specified BMR deviates from the default value, the rationale behind the choice made should be described.*

The BMD (benchmark dose) is the dose corresponding with the BMR of interest.

A 90% confidence interval around the BMD will be estimated, the lower bound is reported by BMDL and the upper bound by BMDU.

# Software Used

Results are obtained using the EFSA web-tool for BMD analysis, which uses the R-package [PROAST](http://www.rivm.nl/en/Documents_and_publications/Scientific/Models/PROAST), version 69.0, for the underlying calculations.

Flowchart for selection of BMDL

# Results

## Response variable: incidence

### Fitted Models

| model | No.par | loglik | AIC | accepted | BMDL | BMDU | BMD | conv |
| --- | --- | --- | --- | --- | --- | --- | --- | --- |
| null | 1 | -83.25 | 168.50 |  | NA | NA | NA | NA |
| full | 5 | -65.01 | 140.02 |  | NA | NA | NA | NA |
| two.stage | 3 | -68.53 | 143.06 | no | NA | NA | 155.0 | yes |
| log.logist | 3 | -69.78 | 145.56 | no | NA | NA | 39.3 | yes |
| Weibull | 3 | -69.42 | 144.84 | no | NA | NA | 42.2 | yes |
| log.prob | 3 | -70.20 | 146.40 | no | NA | NA | 34.0 | yes |
| gamma | 3 | -69.25 | 144.50 | no | NA | NA | 41.1 | yes |
| logistic | 2 | -68.28 | 140.56 | yes | 125 | 189 | 154.0 | yes |
| probit | 2 | -68.37 | 140.74 | yes | 115 | 176 | 142.0 | no |
| LVM: Expon. m3- | 3 | -68.32 | 142.64 | no | NA | NA | 168.0 | yes |
| LVM: Hill m3- | 3 | -68.36 | 142.72 | no | NA | NA | 176.0 | yes |

###

### Estimated Model Parameters

**two.stage**

estimate for a- : 0.05749

estimate for BMD- : 154.8

estimate for c : 512.9

**log.logist**

estimate for a- : 1e-06

estimate for BMD- : 39.32

estimate for c : 0.7342

**Weibull**

estimate for a- : 1e-06

estimate for BMD- : 42.19

estimate for c : 0.7002

**log.prob**

estimate for a- : 1e-06

estimate for BMD- : 34

estimate for c : 0.3759

**gamma**

estimate for a- : 1e-06

estimate for BMD- : 41.1

estimate for c : 0.6458

**logistic**

estimate for a- : -2.919

estimate for BMD- : 153.7

**probit**

estimate for a- : -1.669

estimate for BMD- : 142.5

**EXP**

estimate for a- : 1.482

estimate for BMD- : 167.9

estimate for d- : 1.267

estimate for th(fixed) : 0

estimate for sigma(fixed) : 0.25

**HILL**

estimate for a- : 1.47

estimate for BMD- : 176

estimate for d- : 1.532

estimate for th(fixed) : 0

estimate for sigma(fixed) : 0.25

### Weights for Model Averaging

| two.stage | log.logist | Weibull | log.prob | gamma | logistic | probit | EXP | HILL |
| --- | --- | --- | --- | --- | --- | --- | --- | --- |
| 0.09 | 0.02 | 0.04 | 0.02 | 0.04 | 0.3 | 0.28 | 0.11 | 0.1 |

### Final BMD Values

| subgroup | BMDL | BMDU |
| --- | --- | --- |
| all | 69.5 | 221 |

Confidence intervals for the BMD are based on 200 bootstrap data sets.

### Visualization
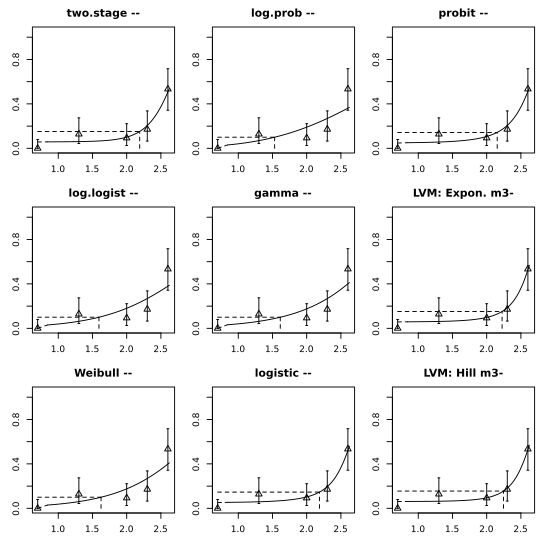

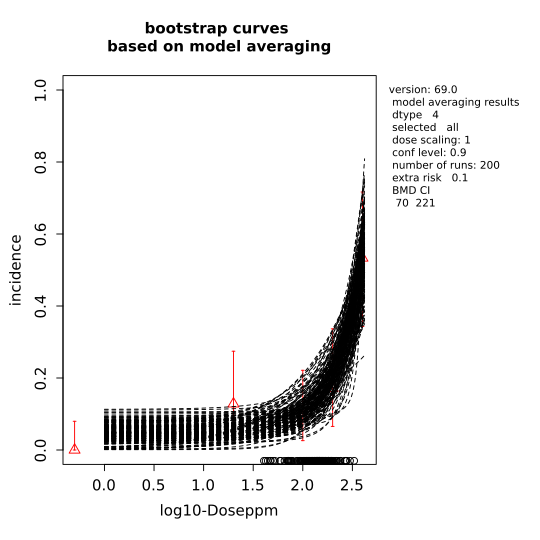

Supplement: Supplementary file 27 — Supplementary file27 (DOCX 118 KB) [file 204_2022_3255_MOESM27_ESM.docx]
