# Supplementary material for: New aspects in deriving health-based guidance values for bromate in swimming pool water
Source: Arch Toxicol. 2022 Apr 6;96(6):1623–59. doi: 10.1007/s00204-022-03255-9 (PMC9095538; doi:10.1007/s00204-022-03255-9)

# Data Description

The endpoint to be analyzed is: incidence.

Data used for analysis:

| Doseppm | incidence | animalno |
| --- | --- | --- |
| 0 | 0 | 36 |
| 20 | 4 | 39 |
| 100 | 1 | 43 |
| 200 | 4 | 35 |
| 400 | 14 | 30 |

*Information pertaining to this endpoint.*

# Selection of the BMR

The BMR (benchmark response) used is an extra risk of 10% compared to the controls.

*When the specified BMR deviates from the default value, the rationale behind the choice made should be described.*

The BMD (benchmark dose) is the dose corresponding with the BMR of interest.

A 90% confidence interval around the BMD will be estimated, the lower bound is reported by BMDL and the upper bound by BMDU.

# Software Used

Results are obtained using the EFSA web-tool for BMD analysis, which uses the R-package [PROAST](http://www.rivm.nl/en/Documents_and_publications/Scientific/Models/PROAST), version 67.0, for the underlying calculations.

| Exp model 5 | 4 | $y=a\cdot(c-(c-1)\exp(-bx^{d}))$ |
| --- | --- | --- |

#

# Results

## Response variable: incidence

### Fitted Models

| model | No.par | loglik | AIC | accepted | BMDL | BMDU | BMD | conv |
| --- | --- | --- | --- | --- | --- | --- | --- | --- |
| null | 1 | -69.19 | 140.38 |  | NA | NA | NA | NA |
| full | 5 | -50.81 | 111.62 |  | NA | NA | NA | NA |
| two.stage | 3 | -54.90 | 115.80 | no | NA | NA | 183 | no |
| log.logist | 3 | -54.01 | 114.02 | yes | 161 | 372 | 230 | yes |
| Weibull | 3 | -54.04 | 114.08 | yes | 160 | 381 | 235 | yes |
| log.prob | 3 | -53.92 | 113.84 | yes | 162 | 369 | 222 | yes |
| gamma | 3 | -53.97 | 113.94 | yes | 162 | 358 | 228 | yes |
| logistic | 2 | -54.56 | 113.12 | yes | 157 | 234 | 192 | yes |
| probit | 2 | -54.86 | 113.72 | yes | 144 | 224 | 177 | yes |
| LVM: Expon. m3- | 3 | -54.15 | 114.30 | yes | 142 | 329 | 246 | yes |
| LVM: Hill m3- | 3 | -54.12 | 114.24 | yes | 149 | 322 | 243 | yes |

### Estimated Model Parameters

**two.stage**

estimate for a- : 0.03602

estimate for BMD- : 183.3

estimate for c : 1e+12

**log.logist**

estimate for a- : 0.04272

estimate for BMD- : 230.1

estimate for c : 3.59

**Weibull**

estimate for a- : 0.04286

estimate for BMD- : 234.9

estimate for c : 3.243

**log.prob**

estimate for a- : 0.04264

estimate for BMD- : 222.4

estimate for c : 1.953

**gamma**

estimate for a- : 0.04282

estimate for BMD- : 227.8

estimate for cc : 5.435

**logistic**

estimate for a- : -3.547

estimate for BMD- : 192.4

**probit**

estimate for a- : -1.947

estimate for BMD- : 176.9

**EXP**

estimate for a- : 1.538

estimate for CED- : 246

estimate for d- : 1.963

estimate for th(fixed) : 0

estimate for sigma(fixed) : 0.25

**HILL**

estimate for a- : 1.537

estimate for CED- : 242.9

estimate for d- : 2.181

estimate for th(fixed) : 0

estimate for sigma(fixed) : 0.25

### Weights for Model Averaging

| two.stage | log.logist | Weibull | log.prob | gamma | logistic | probit | EXP | HILL |
| --- | --- | --- | --- | --- | --- | --- | --- | --- |
| 0.05 | 0.11 | 0.11 | 0.12 | 0.12 | 0.17 | 0.13 | 0.1 | 0.1 |

### Final BMD Values

| subgroup | BMDL | BMDU |
| --- | --- | --- |
|  | 128 | 316 |

Confidence intervals for the BMD are based on 200 bootstrap data sets.

### Visualization
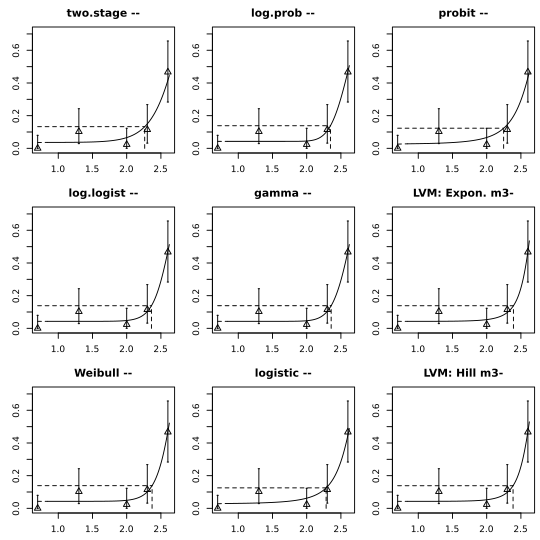

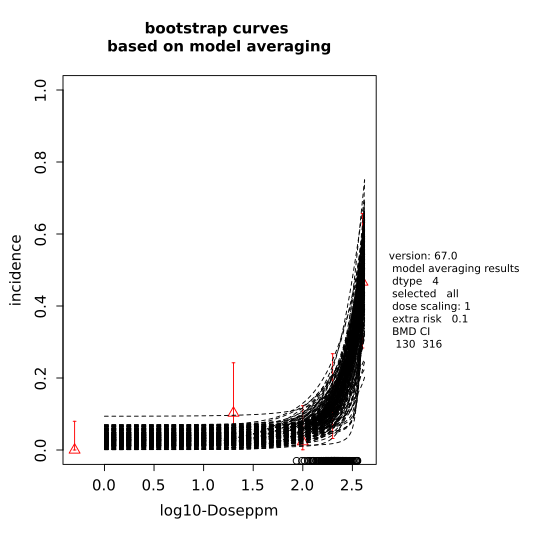

Supplement: Supplementary file 30 — Supplementary file30 (DOCX 107 KB) [file 204_2022_3255_MOESM30_ESM.docx]
