# Supplementary material for: New aspects in deriving health-based guidance values for bromate in swimming pool water
Source: Arch Toxicol. 2022 Apr 6;96(6):1623–59. doi: 10.1007/s00204-022-03255-9 (PMC9095538; doi:10.1007/s00204-022-03255-9)

# Data Description

The endpoint to be analyzed is: incidence.

Data used for analysis:

| Doseppm | incidence | animalno |
| --- | --- | --- |
| 0 | 2 | 53 |
| 250 | 2 | 53 |
| 500 | 7 | 52 |

*Information pertaining to this endpoint.*

# Selection of the BMR

The BMR (benchmark response) used is an extra risk of 10% compared to the controls.

*When the specified BMR deviates from the default value, the rationale behind the choice made should be described.*

The BMD (benchmark dose) is the dose corresponding with the BMR of interest.

A 90% confidence interval around the BMD will be estimated, the lower bound is reported by BMDL and the upper bound by BMDU.

# Software Used

Results are obtained using the EFSA web-tool for BMD analysis, which uses the R-package [PROAST](http://www.rivm.nl/en/Documents_and_publications/Scientific/Models/PROAST), version 67.0, for the underlying calculations.

# Specification of Deviations from Default Assumptions

**General assumptions**

# Results

## Response variable: incidence

### Fitted Models

| model | No.par | loglik | AIC | accepted | BMDL | BMDU | BMD | conv |
| --- | --- | --- | --- | --- | --- | --- | --- | --- |
| null | 1 | -39.92 | 81.84 |  | NA | NA | NA | NA |
| full | 3 | -37.58 | 81.16 |  | NA | NA | NA | NA |
| two.stage | 3 | -37.81 | 81.62 | no | NA | NA | NA | yes |
| log.logist | 3 | -37.58 | 81.16 | no | NA | NA | NA | yes |
| Weibull | 3 | -37.58 | 81.16 | no | NA | NA | NA | yes |
| log.prob | 3 | -37.58 | 81.16 | no | NA | NA | NA | yes |
| gamma | 3 | -37.58 | 81.16 | no | NA | NA | NA | yes |
| logistic | 2 | -39.88 | 83.76 | no | NA | NA | NA | yes |
| probit | 2 | -38.01 | 80.02 | no | NA | NA | NA | yes |
| LVM: Expon. m3- | 3 | -37.58 | 81.16 | no | NA | NA | NA | yes |
| LVM: Hill m3- | 3 | -37.58 | 81.16 | no | NA | NA | NA | yes |

None of the fitted models is better than the null model: All fitted models’ AIC values are larger than null model’s AIC - 2

### Estimated Model Parameters

**two.stage**

estimate for a- : 0.03055

estimate for BMD- : 507.9

estimate for c : 1e+12

**log.logist**

estimate for a- : 0.03773

estimate for BMD- : 499.7

estimate for c : 14.94

**Weibull**

estimate for a- : 0.03773

estimate for BMD- : 499.8

estimate for c : 14.65

**log.prob**

estimate for a- : 0.03773

estimate for BMD- : 499.6

estimate for c : 4.651

**gamma**

estimate for a- : 0.03773

estimate for BMD- : 499.6

estimate for cc : 30.83

**logistic**

estimate for a- : -2.602

estimate for BMD- : 28420

**probit**

estimate for a- : -1.922

estimate for BMD- : 516.3

**EXP**

estimate for a- : 1.568

estimate for CED- : 498.3

estimate for d- : 4

estimate for th(fixed) : 0

estimate for sigma(fixed) : 0.25

**HILL**

estimate for a- : 1.569

estimate for CED- : 498

estimate for d- : 4

estimate for th(fixed) : 0

estimate for sigma(fixed) : 0.25

### Weights for Model Averaging

| two.stage | log.logist | Weibull | log.prob | gamma | logistic | probit | EXP | HILL |
| --- | --- | --- | --- | --- | --- | --- | --- | --- |
| 0.09 | 0.11 | 0.11 | 0.11 | 0.11 | 0.03 | 0.2 | 0.11 | 0.11 |

### Final BMD Values

| subgroup | BMDL | BMDU |
| --- | --- | --- |
|  | 369 | 1790 |

Confidence intervals for the BMD are based on 200 bootstrap data sets.

### Visualization
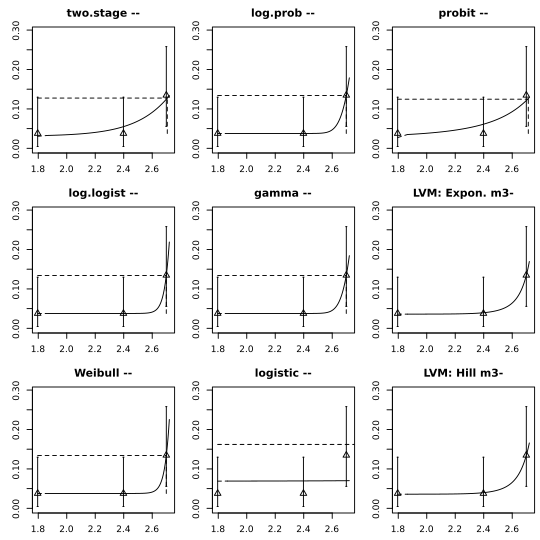

Supplement: Supplementary file 33 — Supplementary file33 (DOCX 56 KB) [file 204_2022_3255_MOESM33_ESM.docx]
