# Supplementary material for: New aspects in deriving health-based guidance values for bromate in swimming pool water
Source: Arch Toxicol. 2022 Apr 6;96(6):1623–59. doi: 10.1007/s00204-022-03255-9 (PMC9095538; doi:10.1007/s00204-022-03255-9)

# Data Description

The endpoint to be analyzed is: incidence.

Data used for analysis:

| Doseppm | incidence | animalno |
| --- | --- | --- |
| 0 | 0 | 19 |
| 15 | 0 | 19 |
| 30 | 0 | 20 |
| 60 | 1 | 24 |
| 125 | 0 | 24 |
| 250 | 3 | 20 |
| 500 | 7 | 20 |

*Information pertaining to this endpoint.*

# Selection of the BMR

The BMR (benchmark response) used is an extra risk of 10% compared to the controls.

*When the specified BMR deviates from the default value, the rationale behind the choice made should be described.*

The BMD (benchmark dose) is the dose corresponding with the BMR of interest.

A 90% confidence interval around the BMD will be estimated, the lower bound is reported by BMDL and the upper bound by BMDU.

# Software Used

Results are obtained using the EFSA web-tool for BMD analysis, which uses the R-package [PROAST](http://www.rivm.nl/en/Documents_and_publications/Scientific/Models/PROAST), version 67.0, for the underlying calculations.

# Results

## Response variable: incidence

### Fitted Models

| model | No.par | loglik | AIC | accepted | BMDL | BMDU | BMD | conv |
| --- | --- | --- | --- | --- | --- | --- | --- | --- |
| null | 1 | -39.02 | 80.04 |  | NA | NA | NA | NA |
| full | 7 | -25.56 | 65.12 |  | NA | NA | NA | NA |
| two.stage | 3 | -27.23 | 60.46 | yes | 137 | 318 | 222 | yes |
| log.logist | 3 | -27.27 | 60.54 | yes | 131 | 337 | 207 | yes |
| Weibull | 3 | -27.25 | 60.50 | yes | 134 | 339 | 213 | yes |
| log.prob | 3 | -27.44 | 60.88 | yes | 121 | 337 | 190 | yes |
| gamma | 3 | -27.27 | 60.54 | yes | 133 | 335 | 207 | yes |
| logistic | 2 | -27.95 | 59.90 | yes | 238 | 372 | 300 | yes |
| probit | 2 | -27.67 | 59.34 | yes | 217 | 355 | 276 | yes |
| LVM: Expon. m3- | 3 | -27.24 | 60.48 | yes | 136 | 349 | 220 | yes |
| LVM: Hill m3- | 3 | -27.25 | 60.50 | yes | 129 | 346 | 218 | yes |

###

### Estimated Model Parameters

**two.stage**

estimate for a- : 1e-06

estimate for BMD- : 222.5

estimate for c : 65.73

**log.logist**

estimate for a- : 1e-06

estimate for BMD- : 207.4

estimate for c : 1.767

**Weibull**

estimate for a- : 1e-06

estimate for BMD- : 212.7

estimate for c : 1.646

**log.prob**

estimate for a- : 1e-06

estimate for BMD- : 189.8

estimate for c : 0.8564

**gamma**

estimate for a- : 1e-06

estimate for BMD- : 206.9

estimate for cc : 1.828

**logistic**

estimate for a- : -4.49

estimate for BMD- : 299.8

**probit**

estimate for a- : -2.415

estimate for BMD- : 276.1

**EXP**

estimate for a- : 2.443

estimate for CED- : 219.7

estimate for d- : 0.4052

estimate for th(fixed) : 0

estimate for sigma(fixed) : 0.25

**HILL**

estimate for a- : 2.28

estimate for CED- : 218

estimate for d- : 0.5941

estimate for th(fixed) : 0

estimate for sigma(fixed) : 0.25

### Weights for Model Averaging

| two.stage | log.logist | Weibull | log.prob | gamma | logistic | probit | EXP | HILL |
| --- | --- | --- | --- | --- | --- | --- | --- | --- |
| 0.1 | 0.1 | 0.1 | 0.08 | 0.1 | 0.14 | 0.18 | 0.1 | 0.1 |

### Final BMD Values

| subgroup | BMDL | BMDU |
| --- | --- | --- |
|  | 162 | 364 |

Confidence intervals for the BMD are based on 200 bootstrap data sets.

### Visualization
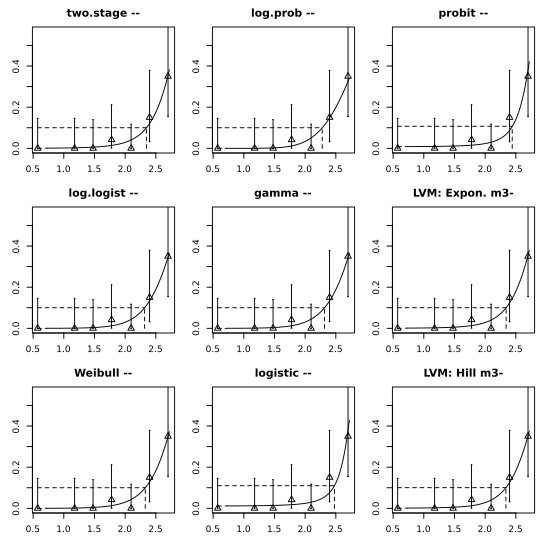

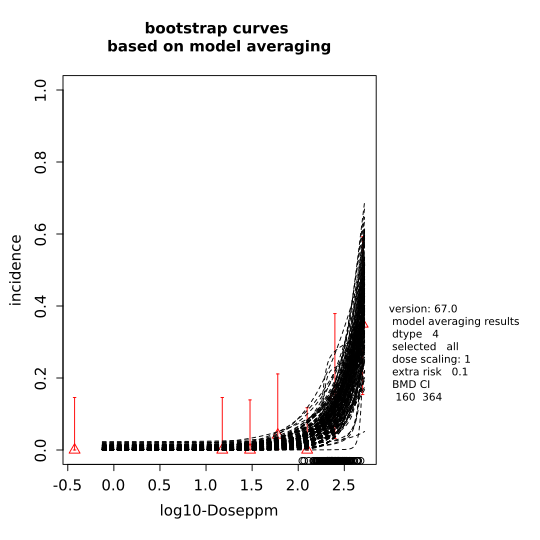

Supplement: Supplementary file 36 — Supplementary file36 (DOCX 108 KB) [file 204_2022_3255_MOESM36_ESM.docx]
